# Autoencoder-based phenotyping of ophthalmic images highlights genetic loci influencing retinal morphology and provides informative biomarkers

**DOI:** 10.1093/bioinformatics/btae732

**Published:** 2024-12-09

**Authors:** Panagiotis I Sergouniotis, Adam Diakite, Kumar Gaurav, UK Biobank Eye and Vision Consortium, Naomi Allen, Tariq Aslam, Denize Atan, Sarah Barman, Jenny Barrett, Paul Bishop, Graeme Black, Tasanee Braithwaite, Roxana Carare, Usha Chakravarthy, Michelle Chan, Sharon Chua, Alexander Day, Parul Desai, Bal Dhillon, Andrew Dick, Alexander Doney, Cathy Egan, Sarah Ennis, Paul Foster, Marcus Fruttiger, John Gallacher, David Garway-Heath, Jane Gibson, Jeremy Guggenheim, Chris Hammond, Alison Hardcastle, Simon Harding, Ruth Hogg, Pirro Hysi, Pearse Keane, Peng Tee Khaw, Anthony Khawaja, Gerassimos Lascaratos, Thomas Littlejohns, Andrew Lotery, Robert Luben, Phil Luthert, Tom Macgillivray, Sarah Mackie, Savita Madhusudhan, Bernadette Mcguinness, Gareth Mckay, Martin Mckibbin, Tony Moore, James Morgan, Eoin O’Sullivan, Richard Oram, Chris Owen, Praveen Patel, Euan Paterson, Tunde Peto, Axel Petzold, Nikolas Pontikos, Jugnoo Rahi, Alicja Rudnicka, Naveed Sattar, Jay Self, Panagiotis Sergouniotis, Sobha Sivaprasad, David Steel, Irene Stratton, Nicholas Strouthidis, Cathie Sudlow, Zihan Sun, Robyn Tapp, Dhanes Thomas, Emanuele Trucco, Adnan Tufail, Ananth Viswanathan, Veronique Vitart, Mike Weedon, Cathy Williams, Katie Williams, Jayne Woodside, Max Yates, Jennifer Yip, Yalin Zheng, Ewan Birney, Tomas Fitzgerald

**Affiliations:** European Molecular Biology Laboratory-European Bioinformatics Institute (EMBL-EBI), Wellcome Genome Campus, Cambridge CB10 1SD, United Kingdom; Division of Evolution, Infection and Genomics, School of Biological Sciences, Faculty of Biology, Medicine and Health, University of Manchester, Manchester M13 9NT, United Kingdom; Manchester Centre for Genomic Medicine, Saint Mary’s Hospital, Manchester University NHS Foundation Trust, Manchester M13 9WL, United Kingdom; Manchester Royal Eye Hospital, Manchester University NHS Foundation Trust, Manchester M13 9WL, United Kingdom; European Molecular Biology Laboratory-European Bioinformatics Institute (EMBL-EBI), Wellcome Genome Campus, Cambridge CB10 1SD, United Kingdom; European Molecular Biology Laboratory-European Bioinformatics Institute (EMBL-EBI), Wellcome Genome Campus, Cambridge CB10 1SD, United Kingdom; European Molecular Biology Laboratory-European Bioinformatics Institute (EMBL-EBI), Wellcome Genome Campus, Cambridge CB10 1SD, United Kingdom; European Molecular Biology Laboratory-European Bioinformatics Institute (EMBL-EBI), Wellcome Genome Campus, Cambridge CB10 1SD, United Kingdom

## Abstract

**Motivation:**

Genome-wide association studies (GWAS) have been remarkably successful in identifying associations between genetic variants and imaging-derived phenotypes. To date, the main focus of these analyses has been on established, clinically-used imaging features. We sought to investigate if deep learning approaches can detect more nuanced patterns of image variability.

**Results:**

We used an autoencoder to represent retinal optical coherence tomography (OCT) images from 31 135 UK Biobank participants. For each subject, we obtained a 64-dimensional vector representing features of retinal structure. GWAS of these autoencoder-derived imaging parameters identified 118 statistically significant loci; 41 of these associations were also significant in a replication study. These loci encompassed variants previously linked with retinal thickness measurements, ophthalmic disorders, and/or neurodegenerative conditions. Notably, the generated retinal phenotypes were found to contribute to predictive models for glaucoma and cardiovascular disorders. Overall, we demonstrate that self-supervised phenotyping of OCT images enhances the discoverability of genetic factors influencing retinal morphology and provides epidemiologically informative biomarkers.

**Availability and implementation:**

Code and data links available at https://github.com/tf2/autoencoder-oct.

## 1 Introduction

Imaging technologies have greatly enhanced the scope and precision of phenotype discovery. A wide range of imaging-derived phenotypes are easily amenable to human identification and are routinely used in biomedical contexts, including in clinical practice (Oren *et al.* 2020). However, to capture the complexity of human biology, there is a need to go beyond traditional clinically-focused and/or expert-curated imaging features (Gong *et al.* 2022).

Artificial neural networks (ANNs) are machine learning models inspired by information processing in biological neural networks (LeCun *et al.* 2015, Hinton 2018, Hasson *et al.* 2020). ANNs can be used to extract granular information from images without introducing certain biases associated with human curation. An autoencoder is a type of ANN that is designed to transform an input set of data into a lower-dimensional code (*i.e*. a set of latent space variables or “embeddings”) and then to recreate the input from the encoded representation (Hinton and Salakhutdinov 2006, Michelucci 2022). Broadly, autoencoders can be used to efficiently compress an image by identifying the key features that lead to optimal reconstruction performance.

The most optically accessible part of the central nervous system is the retina, the multilayered tissue that lines the back of the eyes. The retina is particularly vulnerable to disease, and disruption of its normal architecture (e.g. in conditions like age-related macular degeneration or glaucoma) can lead to visual disability (Sheffield and Stone 2011, Zhao *et al.* 2024). Examination of the retina relies, to a great extent, on imaging, especially the use of optical coherence tomography (OCT). OCT is a noninvasive, non-contact method for cross-sectional imaging that has a resolution approaching that of histopathology (Bouma *et al.* 2022). Application of ANN-based algorithms in OCT image processing is attracting increasing attention with key advantages including the rapid speed, high consistency and quantitative nature of the analyses (De Fauw *et al.* 2018, Yim *et al.* 2020, Keenan *et al.* 2021).

To date, genetic studies of imaging phenotypes have mostly focused on features associated with long-established clinical diagnostic processes (Elliott *et al*. 2018, Xie *et al.* 2024). In our own previous work, we used standardized OCT-derived thickness measurements of the inner (Currant *et al.* 2021) and outer (Currant *et al.* 2023) retinal layers to good effect, discovering previously unreported genetic associations and exploring relationships with disease. Here, we performed genomic analyses on OCT imaging phenotypes extracted using a self-supervised, autoencoder-based approach. We highlight the autoencoder’s ability to derive biologically meaningful phenotypes (with association to genetic variants not seen in previous studies) and to contribute to predictive models for health outcomes such as glaucoma and cardiovascular conditions.

## 2 Materials and methods

### 2.1 Cohort characteristics

We used data from the UK Biobank, a biomedical resource containing genomic and health information from >500 000 individuals from across the United Kingdom (Bycroft *et al.* 2018). UK Biobank participants were recruited between 2006 and 2010 and were, at enrollment, between 40 and 69 years of age. At the initial assessment, UK Biobank volunteers provided consent, answered questions on socio-demographic, lifestyle and health-related factors, completed a range of physical measures, and provided biological samples. DNA was extracted from the donated blood samples and was used to generate genotyping array data. The baseline information has been extended in several ways. For example, repeat assessments were conducted in subsets of the cohort every few years (Bycroft *et al.* 2018). Notably, thousands of UK Biobank participants underwent ophthalmic phenotyping including imaging of the central retina using OCT (>84 000 individuals) (Patel *et al.* 2016, Chua *et al.* 2019). A total of 67 664 individuals were imaged at the time of their baseline visit (Instance 0, “Initial assessment visit (2006–2010)”); this cohort was the focus of the primary analysis. A further 17 090 different participants were imaged for the first time during their first repeat assessment (Instance 1, “First repeat assessment visit (2012–2013)”); these were included in the replication study.

We performed quality control considering genetic and imaging parameters. First, to reduce the impact of population stratification and to increase the validity of the conducted genetic association studies, we focused on individuals within a genetically well-mixed, European-like subset of the UK Biobank. This was achieved by applying principal component analysis (PCA) to UK Biobank genotypic data using standard, previously-implemented methods (Currant *et al.* 2023). Additional participants were excluded as their OCT scans failed to meet a set of previously-described, rigorous quality control criteria (Patel *et al.* 2016, Currant *et al.* 2021, 2023). Finally, participants were removed on the basis of being recommended for exclusion from genetic studies by the UK Biobank or because they were related to third degree or more. The final dataset for the primary analysis included 31 135 study subjects (Supplementary Fig. S1). Similar criteria were used for the replication study with the exception of the imaging quality control parameters which were identical to those described by Zekavat *et al.* (2024).

### 2.2 Generation of thickness maps from OCT volume scans

All the UK Biobank volunteers that were included in our analysis were imaged using the 3D OCT-1000 Mark II device (Topcon, Japan). OCT imaging was carried out in a dark room without pupil dilation using the 3D 6 × 6 mm^2^ macular volume scan mode (128 horizontal B-scans in a 6 × 6 mm raster pattern). The right eye was imaged first (Patel *et al.* 2016, Chua *et al.* 2019). Our analysis focused on left eye images as we assumed that familiarity with the test would have led to scans that, on average, had higher overall quality. A total of 128 PNG images were generated from each tested eye with the dimensions of each PNG image being 650 × 512 × 1 grayscale pixels. After cropping the top (superior) and bottom (inferior) edge of the image area, PNG images with dimensions of 512 × 512 × 1 pixels were obtained.

The 128 images of each OCT scan were used to create a “thickness map”, *i.e*. a single image displaying the retinal thickness throughout the imaged area. To achieve this, segmentation of all the scans in the dataset was performed using a U-Net-based approach. The utilized U-Net method was first described in 2015 (Ronneberger *et al.* 2015) and involves a fully convolutional network that consists of a contracting path (that extracts features) and an expansive path (that localizes objects).

**Figure 1.**
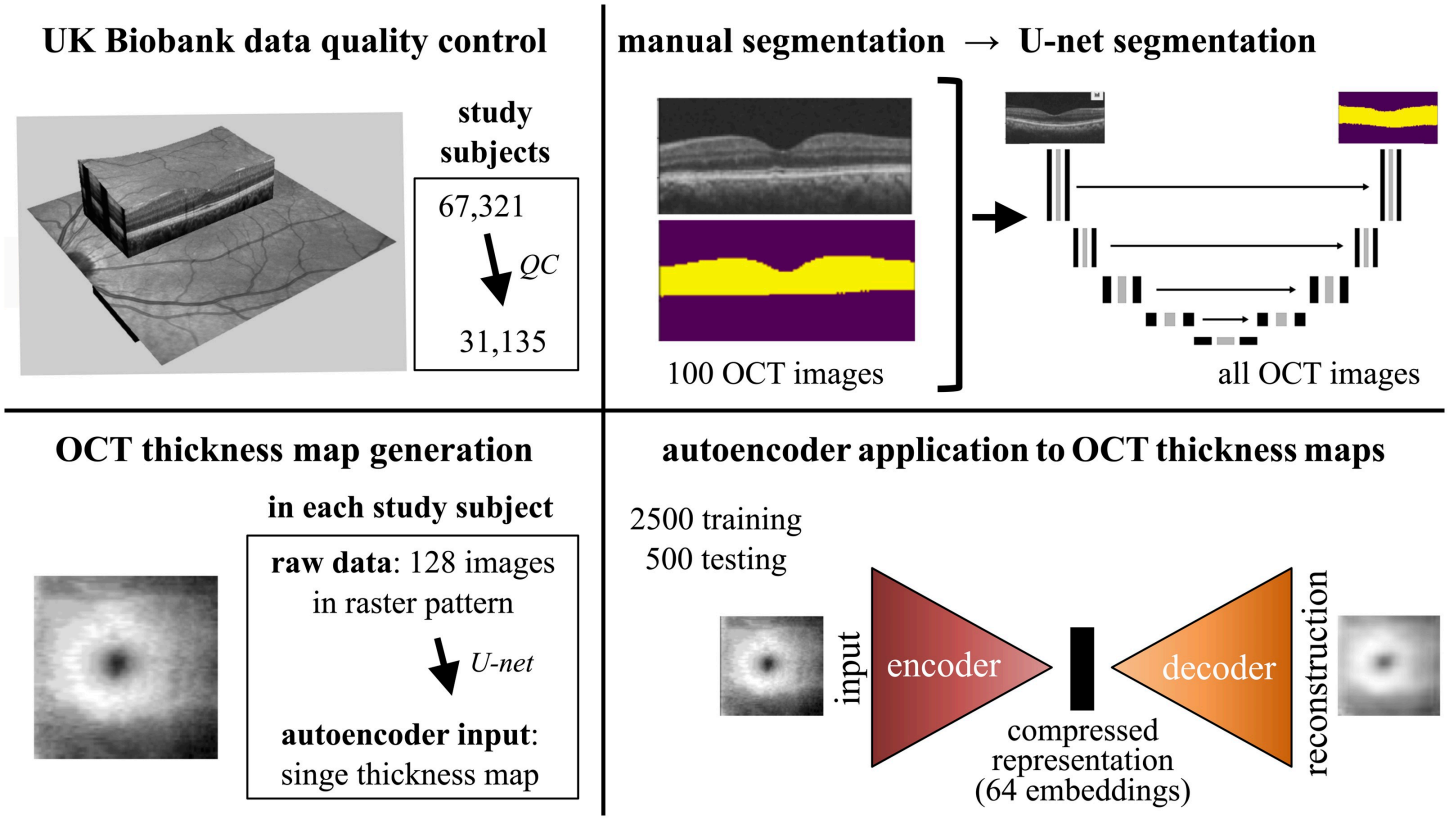
Outline of the experimental approach. OCT images from the central retinae of 67 321 UK Biobank participants were analyzed. After applying quality control (QC) filters considering genetic information and image quality, a cohort of 31 135 study subjects was identified. Aiming to generate retinal “thickness maps” for these individuals, OCT image segmentation was performed using an artificial neural network (U-Net) approach. In brief, 100 OCT images were manually segmented and the generated segmentation masks (examples shown in yellow) were used as input to the U-Net which subsequently segmented all other images. This allowed conversion of the 128 cross-sectional images obtained from each tested eye into a single thickness map image. The thickness maps of the left eyes were then used as input to an autoencoder. This was trained utilizing 2500 training and 500 test images. The output of the embedding network was designed to be a 64-dimensional vector (*i.e.* 64 variables were obtained for each study subject). These 64 autoencoder-derived embeddings were then used for genetic association studies, correlation analyses, and predictive modeling.

Initially, the inner- and outer-most limits of the retina (corresponding to the inner limiting membrane and the Bruch’s membrane, respectively) were manually identified in 100 randomly selected OCT images using the https://www.makesense.ai tool. The original images and the generated “ground truth” segmentation masks were subsequently utilized to train the U-Net. Adaptive moment estimation (Adam) was used to optimize the algorithm for training the network parameters, and training was performed for 50 epochs. The output of the U-Net consisted of segmented OCT images (analogous to the provided masks). These were used to calculate retinal thickness (*i.e.* the vertical distance between the top and bottom edge of the mask in each of the 512 points of the horizontal axis). The obtained measurements were compared to those acquired through the purpose-built Topcon Advanced Boundary Segmentation (TABS) software (the latter are available in the UK Biobank dataset). Good correlation was observed in retinae both with and without pathology, increasing confidence in the utilized approach (Supplementary Fig. S2). Finally, the thickness measurements from the 128 images (“slices”) that were obtained in each tested left eye were combined and used to generate a thickness map for each UK Biobank participant that met the inclusion criteria of this study (Fig. 1).

### 2.3 Autoencoder set-up

An autoencoder was used for self-supervised feature extraction from the 31 135 left eye OCT-derived thickness maps. A conventional autoencoder architecture was utilized (Hinton and Salakhutdinov 2006, Michelucci 2022): the encoder network projected the input images to a low-dimensional space (“latent space”) with 64 variables (“embeddings”), and a function was used to try to reconstruct the original images from these 64 latent space representations. A mean squared error (MSE) loss function was employed to measure the deviation between reconstructed and input data (but otherwise the reconstructed images were not used in the primary analysis). It is noted that the autoencoder was trained end-to-end for 150 epochs utilizing 2500 training and 500 test images. We trialed different autoencoder layouts with bottleneck layers of the following sizes: 128, 64, 32, and 16. For 128 and 64, we obtained very similar reconstruction loss curves during training over 300 epochs. In contrast, for both 32 and 16, the image reconstruction loss could not be dropped below 0.006, suggesting that these models were unable to generalize as well as the larger bottleneck sizes (Supplementary Fig. S3). We then selected a bottleneck size of 64 since this was the smallest size with the best image reconstruction accuracy among the layouts that we tested.

To extract further information from the latent space, PCA (*i.e*. linear dimensionality reduction) was performed using the 64 embeddings as input; the first 25 principal components were then considered for further analyses.

### 2.4 Genome-wide association studies: primary analysis

Genome-wide association studies (GWAS) analyses of the autoencoder-derived embedded features (64 embeddings and 25 embedding-related principal components) were performed using an additive linear model implemented in REGENIE v3.1.1 (https://rgcgithub.github.io/regenie/) (Mbatchou *et al.* 2021). All embedded variables were inverse rank normalized prior to modeling with REGENIE to avoid any potential bias that could be introduced by outlier values (and in an attempt to prevent embeddings with a broader range of variation overshadowing those with a narrower range). The following quality control filters were applied on the imputed genotype data (UK Biobank data-field 22828) during the creation of the whole-genome regression model (REGENIE step 1): a minor allele frequency (MAF)  ≥  5%; Hardy–Weinberg equilibrium test not exceeding *P*  >  1 × 10^−15^; a genotyping rate above 99%; not present in a low-complexity region, a region of long-range linkage disequilibrium or a sex chromosome (Mbatchou *et al.* 2021). This resulted in up to 7 114 193 genotyped variants that were tested for association using a Firth logistic regression model (REGENIE step 2). Correction for the following covariates was undertaken: age at recruitment (data-field 21022), sex (data-field 31), height (data-field 50), weight (data-field 21002), refractive error (calculated as spherical error + 0.5 × cylindrical error; data-fields 5085 and 5086), and genetic principal components 1 to 20 (data-field 22009).

A degree of correlation was expected among autoencoder-derived embeddings so the summary statistics obtained from the GWAS analyses were used to perform a multi-trait meta-analysis. First, embeddings with a high genetic correlation (*i.e*. with Pearson correlation coefficient *R* > 0.9) were identified. Then, the MTAG v1.0.8 tool (https://github.com/JonJala/mtag) (Turley *et al.* 2018) was used to conduct a single meta-analysis for every individual inverse rank normalized embedding, leveraging the findings from correlated embedded features and producing an updated set of GWAS summary statistics for each of these 64 variables. Under certain assumptions, the generated estimates would be expected to be more precise than those obtained from the input GWAS (Turley *et al.* 2018).

To refine the obtained association signals, further analyses were performed using the GCTA-COJO tool (https://yanglab.westlake.edu.cn/software/gcta/#COJO) (Yang *et al.* 2012). These analyses were conducted utilizing linkage disequilibrium estimates from a reference sample (Currant *et al.* 2023) and summary statistics from: (i) the 64 embedding GWAS, (ii) the 25 embedding-related principal component GWAS, (iii) the 64 embedding MTAG-GWAS. Genetic variants in loci that were on different chromosomes or more than 10 Mb distant from each other were assumed to be uncorrelated.

Genetic changes in the main variant set were annotated using Ensembl (Cunningham *et al.* 2022), Open Targets (Ochoa *et al.* 2021), and GWAS Catalog (Sollis *et al.* 2023) data. To accurately summarize the strongest signals, the linkage disequilibrium metrics of the changes that were highlighted as lead variants by GCTA-COJO analysis and were within 1 Mb of one another were manually inspected using the LDlink tool (Myers *et al.* 2020).

### 2.5 Genome-wide association studies: replication

We sought to replicate the genetic associations detected in the primary analysis in a different set of OCT images. As the number of open resources that have sufficiently large human cohorts with combined genomic and OCT imaging data is small, we made use of the UK Biobank “Instance 1” left eye scans (data-field: 21017_1_0). This included images from 17 090 participants that were not part of the discovery/primary cohort and were not used for training of either the U-net segmentation or the autoencoder. It is noted that these additional OCT images were obtained at a different time (2012–2013) compared to the scans in the discovery/primary cohort (2006–2010). Due to the inconsistent capture of certain OCT-related metrics in the replication cohort scans, we used a different set of image QC exclusion criteria. Following the removal of poor quality and outlier images (using the approach described by Zekavat *et al.* (2024)), the replication cohort included 10 439 high-quality scans from unrelated UK Biobank participants of predominantly European-like genetic ancestries (as determined by PCA of genotypes). A replication GWAS was then performed using exactly the same parameters as in the discovery/primary study (outlined above). To gain insights into the extent to which the findings of the primary and the replication study were in agreement, we assessed the degree of correlation between the detected effect size estimates. The relevant beta–beta plots are shown in Supplementary Fig. S4.

### 2.6 Correlation and logistic regression analyses

Direct pairwise comparisons between the 64 embeddings were performed and the relevant Pearson correlation coefficients (*R*) were calculated. Genetic correlation was also estimated, again using Pearson correlation coefficients but this time utilizing the effect size estimates from across the significant associations for all 64 embeddings.

The correlation structure of the embedding space was further studied using hierarchical clustering of the distance matrix between the 64 embeddings. The canonical correlation values for every pairwise test and every embedding were subsequently calculated (Supplementary Fig. S5).

In addition to evaluating the relationship between pairs of the studied embedded features, correlation analyses were performed to look for links between each of these 64 features and four ophthalmic traits (Supplementary Fig. S6). Furthermore, a logistic regression approach was used to look for relationships between embeddings and a set of diseases (high-level ICD10 codes); only the 454 disease-related codes for which there were >1000 cases in the UK Biobank cohort were considered (when factoring in only data obtained after the date of OCT image acquisition (2012)). Age, sex, height, and weight were used as covariates and the statistical significance threshold was determined using Bonferroni correction.

### 2.7 Predictive modeling

Survival analysis was performed using penalized Cox proportional hazard regression; a mixture of L1 and L2 regularization was utilized (often referred to as the Cox elastic net). We focused on two main outcomes—glaucoma and cardiovascular disorders (essential hypertension, angina pectoris, and chronic ischemic heart disease). These included ICD10 codes that were highlighted as significant by the logistic regression analyses described in the previous section and were chosen as predicting them was deemed to be of clinical significance. Only diagnoses assigned after the date of OCT image acquisition were considered. To evaluate discriminative performance, we used Harrell’s C-index as a measure of the concordance between predicted and actual risk. The hyperparameter of L1/L2 penalization strength was set to 0.1, and 20 repetitions of five-fold cross-validation were used to evaluate model performance. Survival curves were estimated using the Kaplan–Meier estimator.

### 2.8 Ethics approval

The UK Biobank has received approval from the National Information Governance Board for Health and Social Care and the National Health Service North West Centre for Research Ethics Committee (Ref: 11/NW/0382). This research was conducted using the UK Biobank Resource under projects 49978, 53144, and 2112. All investigations were conducted in accordance with the tenets of the Declaration of Helsinki.

## 3 Results

### 3.1 Obtaining autoencoder-derived phenotypes from OCT images

After applying standard genetic and OCT quality control filters (Patel *et al.* 2016, Currant *et al.* 2021), we defined a subset of the UK Biobank population that (i) can be considered genetically well-mixed (*i.e*. includes participants that were assigned by genotype PCA to a cluster with subjects of mostly European-like ancestries) and (ii) only contains individuals with high-quality OCT images (Supplementary Fig. S1). This cohort included 31 135 individuals and had a similar sex and age profile to the overall UK Biobank population (Currant *et al.* 2023). Most study subjects were female (54%) and self-identified as White British (91%). The mean age at OCT imaging was 56 years (standard deviation: 8 years).

Study subjects had an OCT “volume scan” of the central retina in each eye. Each volume scan contained 128 cross-sectional images and was generated using a horizontal raster scanning protocol. To extract thickness information and to compress these 128 images into a single retinal “thickness map” we utilized an ANN algorithm involving a U-Net architecture (Ronneberger *et al.* 2015) (Fig. 1; Materials and methods).

The 31 135 left eye retinal thickness maps that we generated were then used as input to an autoencoder. This was trained end-to-end for 150 epochs utilizing 2500 training and 500 test images. We explored various embedding dimensionalities and opted for a 64-dimensional vector (*i.e*. the latent space or “bottleneck layer” contained 64 features) (Fig. 1; Materials and methods). It has been previously shown that this autoencoder architecture can sufficiently represent datasets of similar complexity (Schroff *et al.* 2015, Song *et al.* 2015). A reconstruction error of 0.0037 was obtained (Supplementary Fig. S3).

The univariate distributions of the 64 embeddings are shown in Supplementary Fig. S7. Mostly unimodal or bimodal distributions were observed.

To create an alternative representation allowing information to be combined across different variables within the latent space, we used the 64 embeddings as input to a PCA. The first 25 principal components, representing 98.5% of the variance within the embeddings, were studied further and used for genetic association tests.

### 3.2 Genetic association studies of autoencoder-derived OCT phenotypes

To look for genetic factors associated with the obtained autoencoder-derived embedded features (*i.e.* the 64 embeddings and the first 25 embedding-related principal components), we performed common-variant GWAS. We used REGENIE (Mbatchou *et al.* 2021) and incorporated the following set of covariates into the model: age at recruitment, sex, height, weight, refractive error, and genetic principal components 1 to 20. Notably, each embedded feature was inverse rank normalized prior to performing genetic association testing. As we anticipated a degree of correlation between autoencoder-derived phenotypes, we also conducted a multi-trait meta-analysis using MTAG (Turley *et al.* 2018). This involved identifying genetically correlated embeddings and leveraging these relationships to obtain adjusted GWAS results for each of the 64 embeddings (Materials and methods).

**Figure 2.**
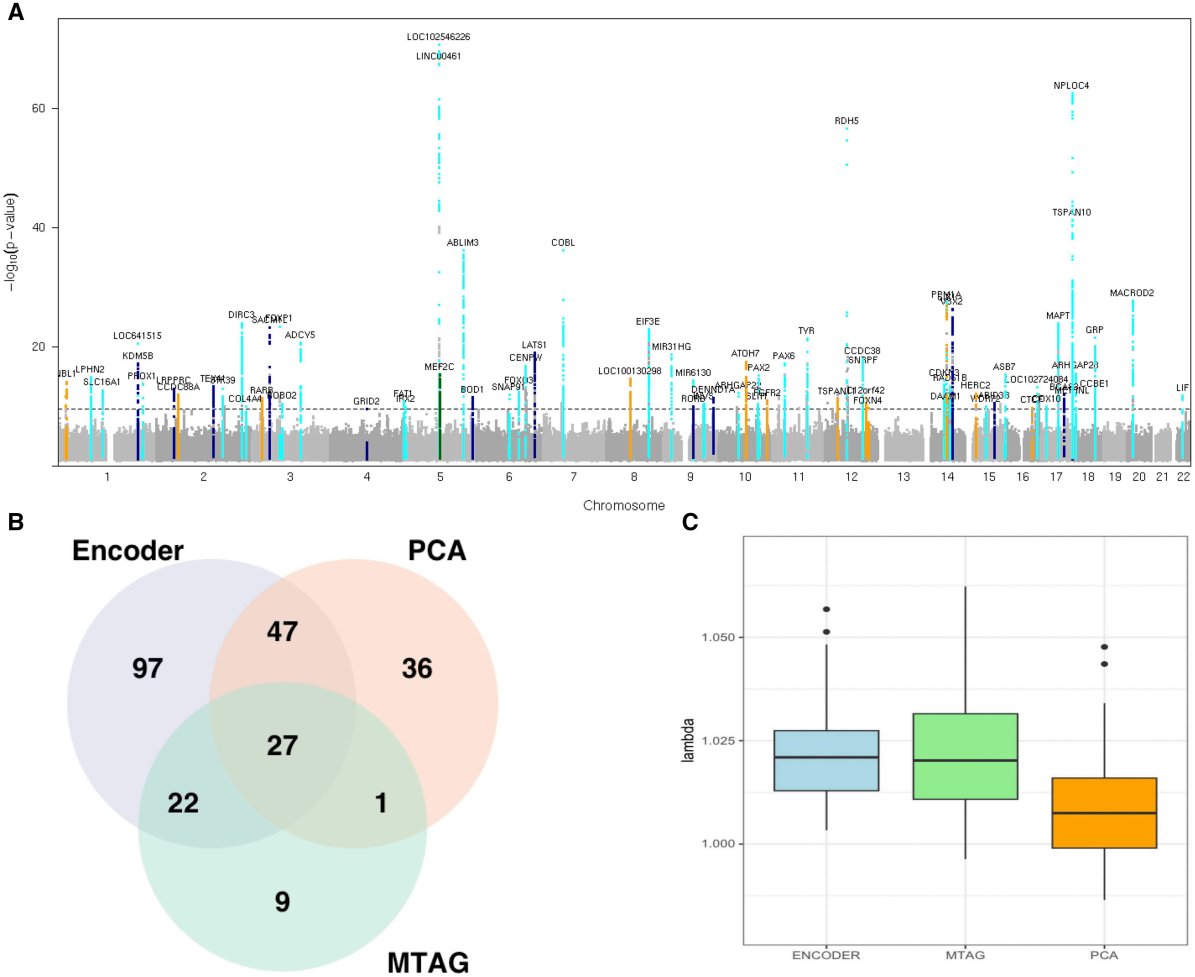
Genome-wide association studies of autoencoder-derived retinal OCT phenotypes (primary analysis). (A) Manhattan plot showing the *P*-values obtained from common-variant GWAS of embedded features (64 embeddings and first 25 embedding-related principal components). Signals that reached genome-wide significance (*P* < 5 × 10^−8^) only in embedding variable analyses are highlighted with dark blue. Signals that reached genome-wide significance only in analyses of embedding-related principal components are highlighted with orange. Signals that reach genome-wide significance only in MTAG of embedding variables are highlighted with green. All other genome-wide significant signals are highlighted with cyan. (B) Venn diagram shows the overlap of lead signals among: conventional GWAS of the 64 embeddings (“encoder” group in light blue); MTAG of the 64 embeddings (“MTAG” group in light green) and conventional GWAS of the first 25 embedding-related principal components (“PCA” group in light orange). (C) Genomic inflation factor lamda (λ) for 64 embedding-, 64 MTAG- and 25 PCA-GWAS (median λGC = 1.016).

Overall, 418 312 association signals from 17 022 common variants reached the genome-wide significance threshold (*P* < 5 × 10^−8^) (Table 1; Fig. 2). These merged into 239 lead loci following analysis with GCTA-COJO (conditional and joint multiple-variant analysis) (Yang *et al.* 2012) (Supplementary Table S1); 118 of these remained significant when a conservative/higher (“study-wide”) threshold was used to account for all the different association routes that were utilized (*P* < 3.2 × 10^−10^ following Bonferroni correction for 153 tests).

**Table 1.** Comparative analyses of conventional and MTAG GWAS results (primary analysis)

	GWAS	MTAG	GWAS
	64 embeddings	64 embeddings	25 PCAs
Total genetic variants (*P* < 5 × 10^−8^)	14 885	9 520	11 075
Lead genetic variants (*P* < 5 × 10^−8^)	443	99	157

GWAS, genome-wide association study; MTAG, multi-trait analysis of GWAS; PCA, principal component analysis.

A replication study was conducted using OCT scans from 10 409 UK Biobank participants that were not included in the primary analysis. There was a high level of concordance in the findings of the two association studies (Supplementary Fig. S4). A total of 41 loci passed both the conservative study-wide threshold (*P* < 3.2 × 10^−10^) in the primary analysis and a Bonferroni correction based threshold (*P* < 8.5 × 10^−5^ following Bonferroni correction for 118 tests) in the replication study. Most of these loci encompass variants previously linked to retinal layer thickness parameters (including around *LINC00461*, *TSPAN10*, and *COBL*) (Gao *et al.* 2019, Currant *et al.* 2021, 2023) while a subset of them has also been linked to monogenic retinal disorders [including *RDH5* (retinal dystrophy), *TYR* (albinism), and *GNB3* (congenital stationary night blindness)] (Table 2; Supplementary Table S1).

**Table 2.** Summary of the 10 top-ranking loci associated with autoencoder-derived retinal OCT phenotypes.^a^

Top-ranking common variant in locus	**Chr: position** (grch37)	Key gene(s)	**Allele freq** (ukb)	Minimum *P*-value	Embeddings with significant result for the locus	**Selected previous association(s) with the detected significant variants in the locus** GWAS catalog; (Panelapp)
rs17421627	5:87847586	*LINC00461*	0.07	4 × 10^−68^	83	Retinal thickness measurements, retinal vascular fractal density
rs62075722	17:79611271	*TSPAN10/NPLOC4/PDE6G*	0.65	1 × 10^−62^	83	Retinal thickness measurements, refractive error, eye color, hair color
rs3138142	12:56115585	*RDH5/CD63*	0.24	1 × 10^−56^	91	Retinal thickness measurements, refractive error, retinal vascular fractal density; (retinal dystrophy)
rs13171669	5:148601243	*AFAP1L1/ABLIM3*	0.43	1 × 10^−36^	84	Retinal thickness measurements, height, waste-hip ratio, lung function
rs12719025	7:51100190	*COBL*	0.46	1 × 10^−36^	113	Retinal thickness measurements, refractive error
rs33912345	14:60976537	*SIX6/C14orf39/PPPM1A*	0.61	4 × 10^−28^	6	Retinal thickness measurements, glaucoma, height; (ocular malformations)
rs887595	14:74666641	*VSX2/LIN52*	0.82	6 × 10^−27^	85	Retinal thickness measurements; (microphthalmia)
rs17279437	3:45814094	*SLC6A20*	0.11	8 × 10^−24^	33	Retinal thickness measurements, macular telangiectasia, brain measurements, metabolite measurements; (hyperglicynuria)
rs1042602	11:88911696	*TYR*	0.37	5 × 10^−22^	29	Retinal thickness measurements, brain measurements, skin color, hair color; (albinism)
rs62175360	2:218520035	*DIRC3*	0.07	9 × 10^−22^	21	Retinal thickness measurements, optic disc measurements, brain measurements, metabolite measurements, height, cancer

a The above loci were identified after selecting fine mapped variants that had a *P* < 3.2 × 10^−10^ in the primary analysis and a *P* < 5 × 10^−8^ in the replication study. Manual inspection of linkage disequilibrium patterns was subsequently performed to further refine the signals and the 10 loci with the lowest *P*-value were selected. UKB, UK Biobank.

For each of the 118 lead loci that were found to be significant in the primary analysis (*P* < 3.2 × 10^−10^), we compared the retinal thickness maps of heterozygotes for the key variant to that of homozygotes. Interestingly, some genetic alterations appeared to have recessive effects (e.g. rs62075722) while others appeared to have dominant effects (e.g. rs11051131); topographical variation was also noted (Supplementary File S1).

**Figure 3.**
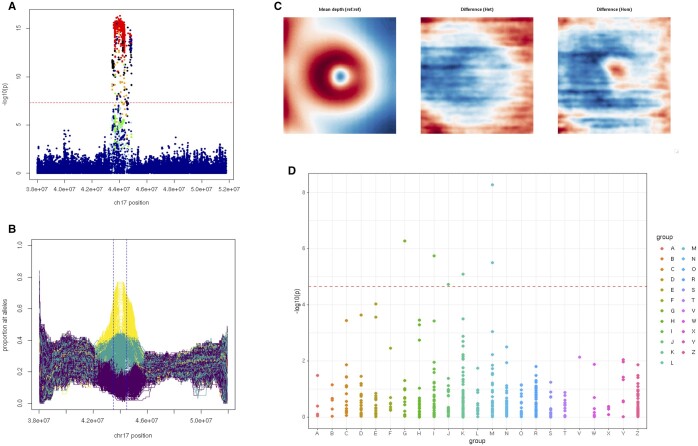
Analysis of the chromosome 17q21.31 inversion association signal. (A) Genetic association study result highlighting a group of 2,936 common variants that passed the genome-wide significance threshold for MTAG of embedding no.21. The genetic alterations are colored based on their linkage disequilibrium (LD; R^2^) relationship to the inversion genotype. (B) Classification of the inversion status based on the pattern of alternative alleles across the 17q21.31 region for 487 409 UK Biobank participants. (C) Left eye retinal thickness maps showing the difference in retinal structure between individuals with different inversion-related alleles. Left: mean depth (thickness) representation for reference:reference (no inversion) alleles. Middle: difference between image mean for reference:reference and image mean for reference:inversion (heterozygous inversion) genotypes. Right: difference between image mean for reference:reference and image mean for inversion:inversion (homozygous inversion) genotypes. A paracentral area of differential retinal thickness can only be visualized in the reference-to-homozygous difference map (in keeping with a recessive effect). (D) Phenome-wide associations for the inversion genotype against 454 ICD10 disease codes for which there were >1000 cases in the UK Biobank cohort (when only data obtained after the date of OCT image acquisition were considered); six codes (M16, G20, I84, M20, K60, J84) remained significant after Bonferroni correction; −log_10_*P*-values are shown grouped by high-level ICD10 category.

Our primary analysis identified notable associations between multiple embeddings and a locus encompassing the *MAPT* (microtubule-associated protein tau) gene. The detected signal appears to be driven by a common ancestral genomic inversion at 17q21.31 (Fig. 3A) (Stefansson *et al.* 2005, Espinosa *et al.* 2023). Using the pattern of alternative alleles across this genomic region, we were able to classify 487 409 UK Biobank participants as either reference:reference (no inversion), reference:inversion (heterozygous inversion), or inversion:inversion (homozygous inversion) (Fig. 3B). In accordance with previous studies (Steinberg *et al.* 2012), we found that the 17q21.31 inversion is common in individuals of European-like ancestries, rare in individuals of African-like ancestries and very rare in Asian-like populations (allele frequency of 0.22, 0.01, and 0.004 respectively). When we compared the retinal thickness profiles between study subjects that carry heterozygous and homozygous inversion genotypes, we found that the 17q21.31 inversion appears to affect retinal thickness in an apparently recessive pattern (Fig. 3C). We then performed a phenome-wide association study (PheWAS) of the 17q21.31 inversion using disease-related ICD10 codes. After Bonferroni correction, we found six statistically significant signals for ICD10 codes, including one for Parkinson disease (G20; *P* = 5.3 × 10^−7^; β −0.61) (Fig. 3D). When we re-run this analysis, this time under a recessive model, we observed a marginal increase in signal strength (median increase of 0.74 on −log_10_*P*) and found statistically significant signal for one additional ICD10 code: E66 (obesity).

### 3.3 Investigating how autoencoder-derived OCT phenotypes are related between them and with other retinal traits and diseases

**Figure 4.**
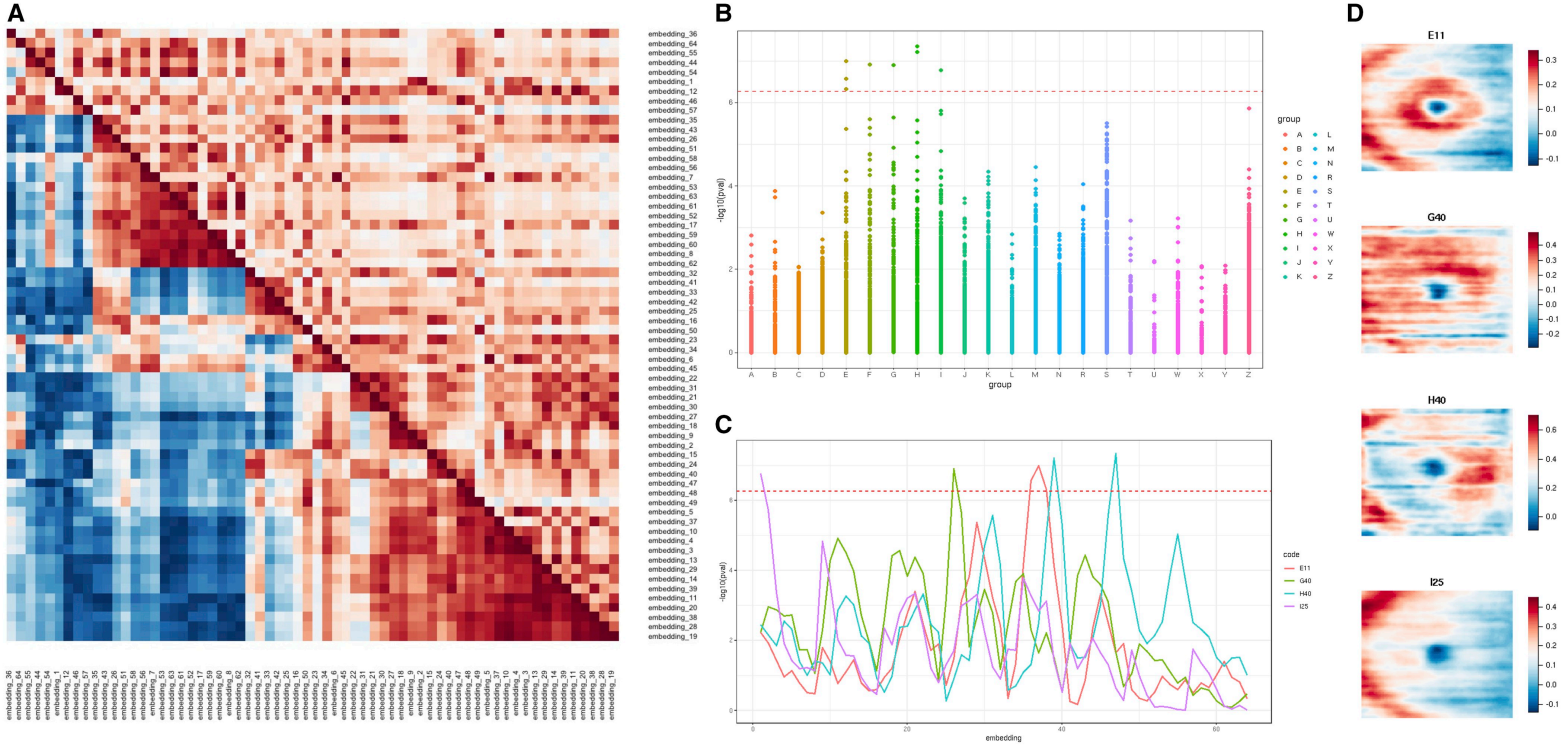
Correlation and logistic regression analyses of autoencoder-derived retinal OCT phenotypes. (A) Direct (upper triangle) and genetic (lower triangle) correlations among embedded features (64 embeddings). The two correlation matrices are displayed using a heatmap where rows and columns were ordered by the distances obtained via hierarchical clustering (on the embedding value correlation matrix only). (B) Logistic regression analysis of the 64 embeddings against high-level ICD10 disease codes; only data obtained after the date of OCT image acquisition were included and only ICD10 codes for which there were >1000 cases in the UK Biobank cohort were considered; sex, age, height, and weight were factored in as covariates. A total of eight signals for five distinct ICD10 codes remained significant after Bonferroni correction: E11 (3), G40 (1), H40 (2), I25 (1), F10 (1). (C) Graph showing which specific embeddings were significantly correlated with the lead signals of the logistic regression analysis, *i.e.* non-insulin-dependent diabetes (E11), epilepsy (G40), glaucoma (H40) and chronic ischemic heart disease (I25); −log_10_*P*-values are shown for all 64 embedded features. (D) Left eye retinal thickness maps showing the difference in retinal structure between UK Biobank participants who were diagnosed with non-insulin-dependent diabetes (E11; first row), epilepsy (G40; second row), glaucoma (H40; third row), and chronic ischemic heart disease (I25; fourth row) after having an OCT scan against the groups of individuals that have not been assigned the relevant ICD10 codes.

To gain insights into the nature of the autoencoder-derived embedded features, we performed correlation and logistic regression analyses. First, we examined the direct pairwise correlation between the 64 embeddings; a few prominent clusters were noted (Fig.4A —upper triangle; Supplementary Fig. S5). Then we looked at genetic correlation (Fig. 4A—lower triangle); a notable observation was the discrepancy between the degree of direct and genetic correlation for many groups of embeddings. This suggests that although the latent space is complex and includes (linearly) correlated features, the different embeddings are able to represent discrete factors related to different aspects of retinal morphology genetics.

We subsequently investigated the relationship between the 64 embedded features and a set of traits and disease codes (ICD10) that are available in the UK Biobank dataset. Unsurprisingly, most embeddings correlated with retinal layer thickness parameters (Supplementary Fig. S6). We then used a logistic regression approach (with sex, age, height, and weight as covariates) and detected significant associations between specific embeddings and the following conditions: non-insulin-dependent diabetes, epilepsy, glaucoma, and chronic ischemic heart disease (Fig. 4B). Two of these lead signals (epilespy and chronic ischemic heart disease) are associated very specifically to only one embedding each (embedding no. 1 and no. 26, respectively). In contrast, glaucoma is associated with two different embeddings (no. 39 and no. 47) and diabetes to three sequential embeddings (nos. 36–38) (Fig. 4C). Reassuringly, GWAS analysis of embeddings no. 36–38 revealed statistically significant signals linked to *ADCY5* (Supplementary Table S1), a gene that influences glucose metabolism and has been previously linked to non-insulin-dependent diabetes by multiple association studies (Roman *et al.* 2017).

To understand which aspects of retinal morphology drove the association between the embedded features and the lead disease codes (non-insulin-dependent diabetes, epilepsy, glaucoma, and chronic ischemic heart disease), we inspected a set of retinal thickness difference maps. These compared retinal thickness in UK Biobank participants that had been assigned the relevant ICD10 code (after OCT imaging) to those that have not (Fig. 4D). In keeping with previous observations: (i) the main areas of difference for diabetes were the paracentral region and the areas temporal to the optic disc (corresponding to the major retinal vessels) (Li *et al.* 2021); (ii) the main area of difference for glaucoma corresponded to what is described in the glaucoma literature as the “macular vulnerability zone” (Hood 2017).

### 3.4 Using autoencoder-derived OCT phenotypes to gain insights into disease risk

**Figure 5.**
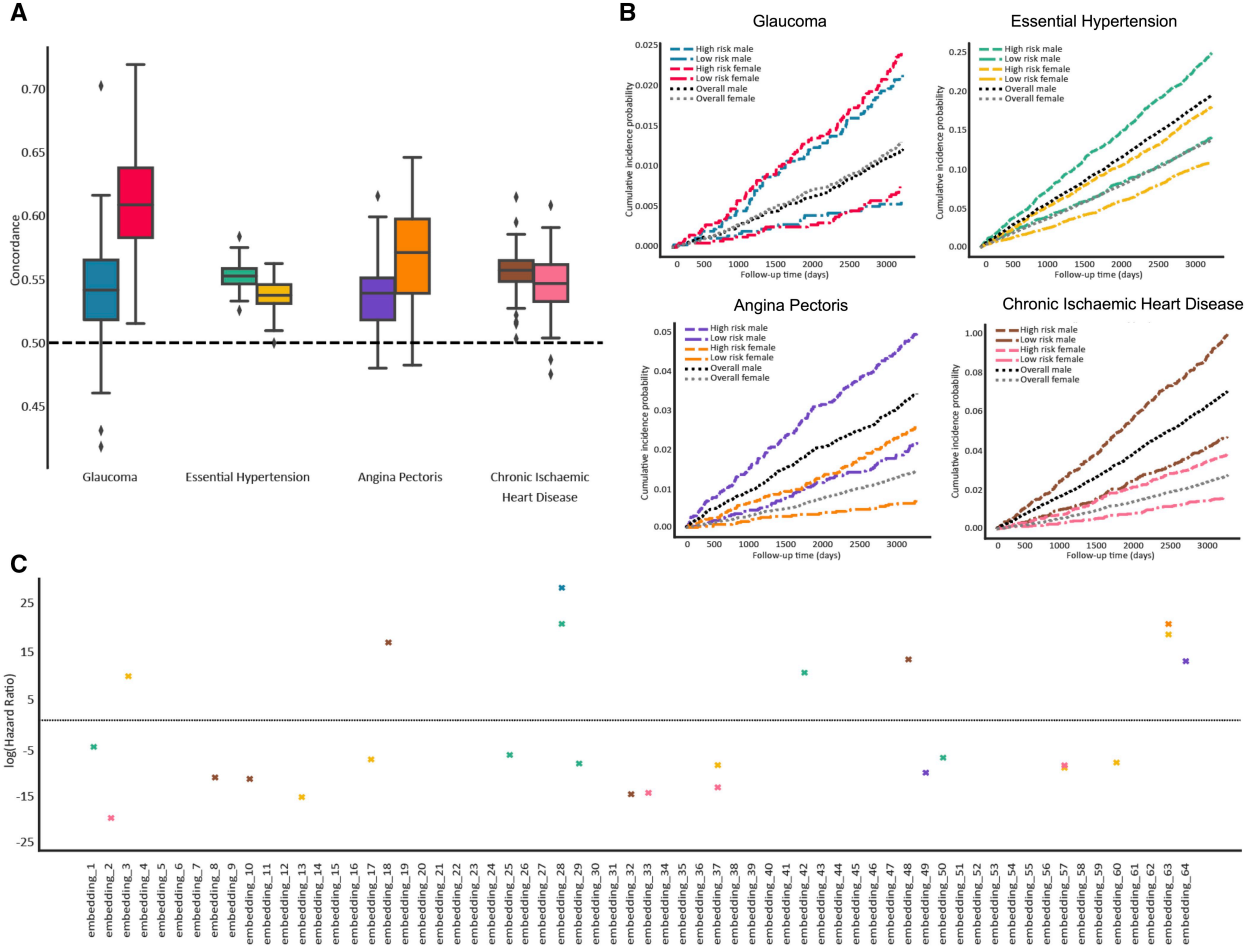
Survival analysis investigating the contribution of embedded features upon the time-to-diagnosis for four ICD10 disease codes. (A) Concordance index evaluating the embedding-incorporating model’s ability to discriminate sex-stratified disease occurrence; the distribution across 20 repetitions of five-fold cross-validation is shown (*n* = 100 for each box plot); all box plots demarcate quartiles and median values, while whiskers extend to 1.5× of the interquartile range. (B) Kaplan–Meier plots showing sex-stratified risk of disease occurrence for the overall population as well as for high-risk cohorts determined by the embedding-incorporating model (top 25% based on Cox regression). (C) Graph highlighting which embedded features have a significant relationship with the selected diseases in male and female cohorts; −log_10_ hazard ratios are shown.

We investigated if autoencoder-derived embedded features from an individual’s OCT scan can help predict the occurrence of certain diseases, including glaucoma and cardiovascular disorders. We used survival analysis (Cox proportional hazard regression) and found significant links between specific embeddings and the occurrence of disease (after the OCT scan date) (Fig. 5A). High-risk cohorts identified based on the embedded features showed a higher chance of being affected by glaucoma or cardiovascular conditions compared to the sex-stratified baseline rate of disease occurrence. In other words, the embedded features could help identify high-risk cohorts (Fig. 5B). It is highlighted that a few embeddings appear to be linked to multiple diseases (e.g. no. 28), while others have no effect on any disease or are specific to single disease codes (e.g. embedding no. 18 for chronic ischemic heart disease). A notable observation is the link between multiple embeddings and essential hypertension. This is often in the presence of signals from other cardiovascular disease codes, suggesting that changes in blood pressure can lead to alterations in OCT-evaluated retinal structure which may in turn be a marker for the development of cardiovascular complications (Fig. 5C).

## 4 Discussion

Phenotypes are abstract entities that can be thought of as simplified maps carved from higher dimension spaces (Cortese *et al.* 2021). These maps are generally influenced by a combination of genetic, environmental, and stochastic factors. Discovering phenotypes that represent distinct biological pathways and/or have pragmatic medical significance is of particular interest (Dahl and Zaitlen 2020). Here, we show that a computational, autoencoder-based approach can be used to efficiently extract informative phenotypes from retinal OCT images.

Analysis of the genetic basis of autoencoder-derived embedded features revealed 118 statistically significant (*P* < 3.2 × 10^−10^) association signals. Notably, three recent studies that used a similar analytical approach but focused on different imaging modalities—fundus photography (Kirchler *et al.* 2022, Xie *et al.* 2024) and cardiac magnetic resonance images (Bonazzola *et al.* 2024)—identified a slightly smaller number of genetic associations (Supplementary Table S2). While many of the loci detected here have prior links to retinal phenotypes, at least 20 of them have no such prior association. One example is the locus around *LPHN2/ADGRL2*, a gene encoding a synaptic adhesion molecule implicated in guiding neural circuit connectivity (Donohue *et al.* 2021) (lead marker: rs1492258; association with seven autoencoder-derived embedded features; minimum *P* 1.4 × 10^−15^). Although this gene is expressed in the retina, especially in the bipolar cells (Karlsson *et al.* 2021), it has not been previously associated with a retinal phenotype.

Reassuringly, there was a significant overlap between the findings of the present study and the results of previous analyses that investigated the genetic architecture of traditional OCT-derived retinal phenotypes. These include three UK Biobank studies: (i) one that looked at macular (*i.e.* total central retinal) thickness and reported 139 loci (Gao *et al.* 2019), and (ii) two from our group that investigated OCT-derived measurements of inner and outer retinal layers, and reported 46 and 111 loci, respectively (Currant *et al.* 2021, 2023). Overall, 36% (98/273) of the combined lead loci from these studies also reached genome-wide significance in the present analysis (58%, 33%, and 41% for Currant *et al.* 2021, 2023 and Gao *et al.* 2019, respectively). Interestingly, the two signals with the highest statistical significance in the macular thickness GWAS conducted by Gao *et al.* (2019) were also the most significant hits in this study (Fig. 2). The marker with the highest statistical significance was within the *LINC00461* locus. *LINC00461* is a long noncoding RNA that is the primary transcript of miR-9-2. *LINC00461* is highly expressed in neural stem cells and a decrease in its expression has been shown to alter the timing of retinal neurogenesis (Thomas *et al.* 2022). The locus with the second highest statistical significance encompassed the *TSPAN10* gene. In the eye, *TSPAN10* is predominantly expressed in melanin-containing cells (retinal pigment epithelia (RPE) and uveal melanocytes), and the corresponding protein is thought to have a role in regulating retinal cell fate and development (Dornier *et al.* 2012, Haining *et al.* 2012, Orozco *et al.* 2020). Further functional genomic analyses of these two key loci are expected to provide important insights into developmental processes shaping human retinal morphology.

An intriguing association that we detected was that between certain autoencoder-derived retinal phenotypes and a common 17q21.31 inversion encompassing the *MAPT* gene. *MAPT* is primarily expressed in brain neurons, and genetic alterations impacting the *MAPT* locus have been linked to several neurodegenerative disorders including Alzheimer disease, frontotemporal dementia and parkinsonism (Wang and Mandelkow 2016, Shi *et al.* 2021). Recently, inner retinal layer thickness parameters and glaucoma have been added to the growing list of phenotypes associated with the *MAPT* locus (Gharahkhani *et al.* 2021, Diaz-Torres *et al.* 2023). Further work is required to pinpoint which (and how many) genes within the *MAPT* region are causally associated with retinal and brain phenotypes (Diaz-Torres *et al.* 2023). More broadly, the extent to which the overlap between neurodegenerative disorders, retinal morphology, and glaucoma reflects pleiotropy rather than causal relationships remains to be determined. Of note, causal genetic effects in both directions have been previously suggested between retinal imaging traits and Alzheimer disease (Zhao *et al.* 2024) while little support has been found for a causal relationship between glaucoma and Alzheimer disease (Budu-Aggrey *et al.* 2020).

Deep learning approaches have been shown to be able to detect imaging patterns that are not amenable to human identification and which can assist with prediction tasks (Patel *et al.* 2024, Radhakrishnan *et al.* 2023, Zhou *et al.* 2024, Yun *et al.* 2024). For example, neural networks can predict sex and age with good accuracy from retinal OCT images (Chueh *et al.* 2022, Le Goallec *et al.* 2022), whereas human experts find these tasks impossible. Here, we investigated if autoencoders can identify OCT parameters that can be used to predict health outcomes (glaucoma and cardiovascular disease). Although the overall predictive ability of the generated models was moderate, the autoencoder-derived features were shown to enhance risk stratification. These observations suggest that it is not inconceivable that purpose-built autoencoders will play a role in improving the efficiency of medical screening programs in the future.

This study has a number of limitations. First, the autoencoder input was retinal thickness maps generated using a U-Net approach which made our framework semi-automated (as a small amount of manual labeling was required). Using three-dimensional autoencoders to extract features directly from OCT volume scans could fully automate the pipeline, minimizing any subjective aspects and reducing the burden of data curation (Diaz-Pinto *et al.* 2022). Second, an empirical approach was used to determine the number of embedded features that were analyzed; this was guided by observations regarding the information-content and variance captured. Third, we only performed common-variant genetic association analyses of the obtained embedded features. The increasing availability of genome sequencing data in UK Biobank participants will allow us to more comprehensively look for genetic associations, including with rare variants and with copy number alterations in the future. Third, the fact that relationships were detected between embeddings and certain health outcomes does not necessarily imply causation. The main aim of this study was to assess if autoencoders can be utilized to produce biologically and clinically relevant phenotypes. In-depth confounder adjustment and causal inference studies were therefore not performed. Furthermore, the predictive models described here have a proof-of-concept nature and are not intended for implementation (especially as the data used for training and evaluation were highly homogeneous and focused on individuals with predominantly European-like ancestries).

In summary, this study proposes a framework for retinal phenotyping based on a self-supervised deep learning approach. Our findings highlight that autoencoder-based techniques can be used to extract knowledge about the genetic factors determining retinal morphology. The outlined approach is flexible and can be adapted and extended to other organs and imaging modalities.

## Supplementary Material

btae732_Supplementary_Data

## Data Availability

The UK Biobank dataset is available under restricted access through a procedure described at http://www.ukbiobank.ac.uk/using-the-resource/. All other data supporting the findings of this study are available in the article and in its online supplementary material.
