# Supplementary material for: Autoencoder-based phenotyping of ophthalmic images highlights genetic loci influencing retinal morphology and provides informative biomarkers
Source: Bioinformatics. 2024 Dec 9;41(1):btae732. doi: 10.1093/bioinformatics/btae732 (PMC11751639; doi:10.1093/bioinformatics/btae732)
Supplement: btae732_Supplementary_Data [file btae732_supplementary_data.zip › BIOINF-2024-1380_suppl-file1.pdf]

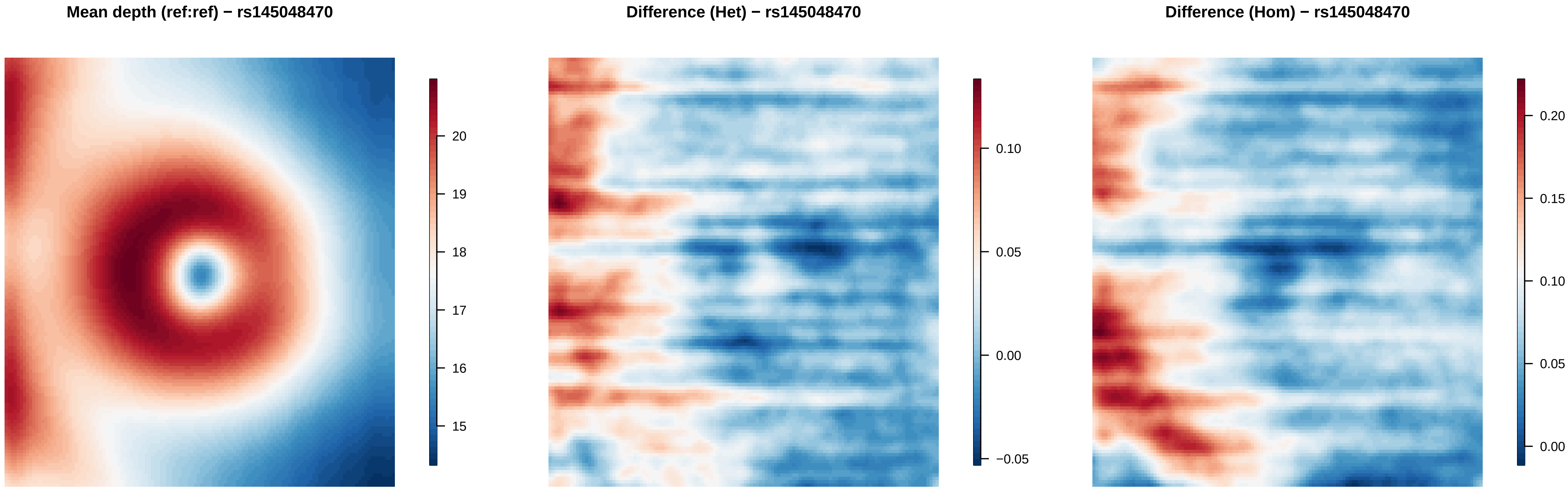
Mean depth (ref:ref) − rs145048470
20
19
18
17
16
15
Difference (Het) − rs145048470
0.10
0.05
0.00
−0.05
Difference (Hom) − rs145048470
0.20
0.15
0.10
0.05
0.00

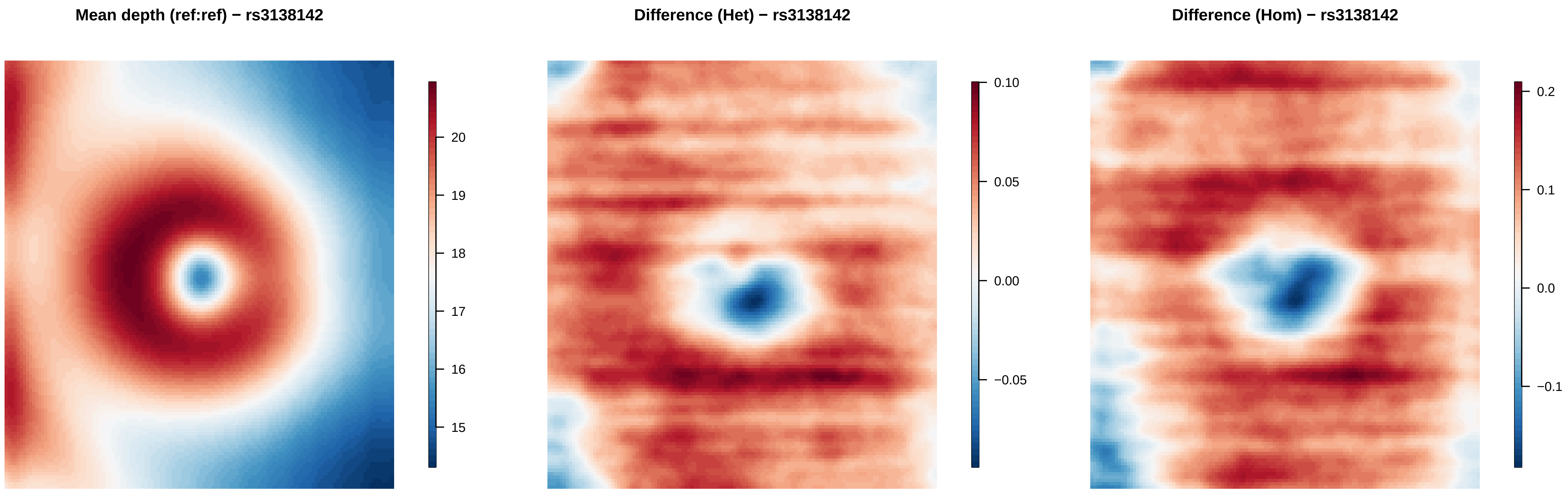
Mean depth (ref:ref) − rs3138142
20
19
18
17
16
15
Difference (Het) − rs3138142
0.10
0.05
0.00
−0.05
Difference (Hom) − rs3138142
0.2
0.1
0.0
−0.1

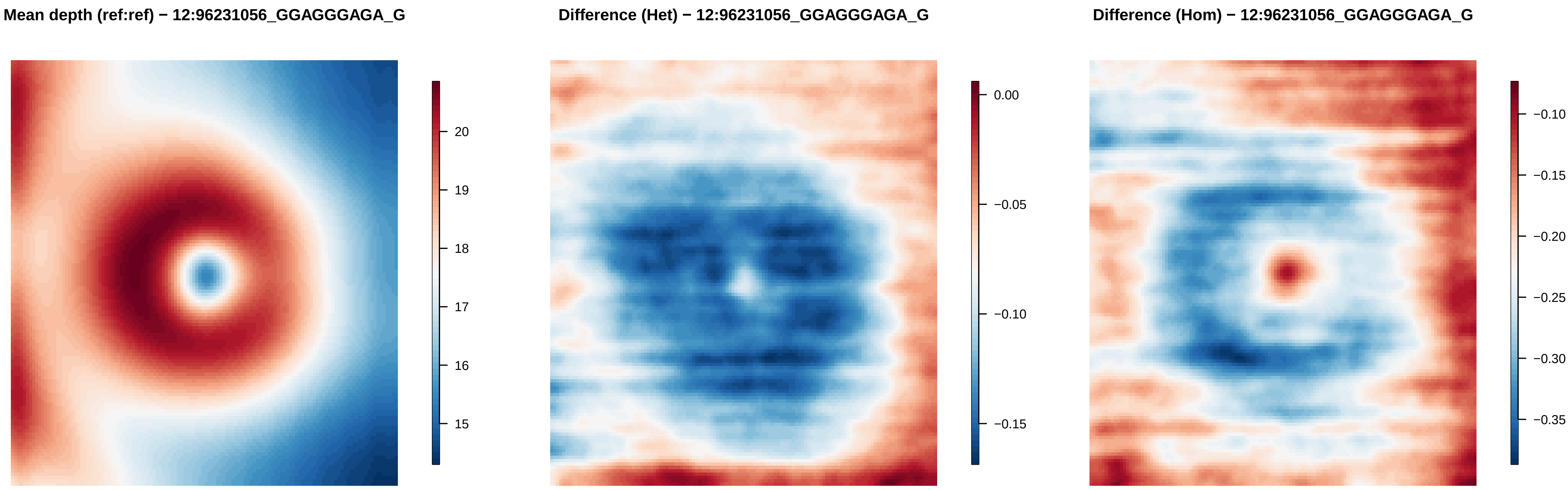
Mean depth (ref:ref) − 12:96231056_GGAGGGAGA_G
20
19
18
17
16
15
Difference (Het) − 12:96231056_GGAGGGAGA_G
0.00
−0.05
−0.10
−0.15
Difference (Hom) − 12:96231056_GGAGGGAGA_G
−0.10
−0.15
−0.20
−0.25
−0.30
−0.35

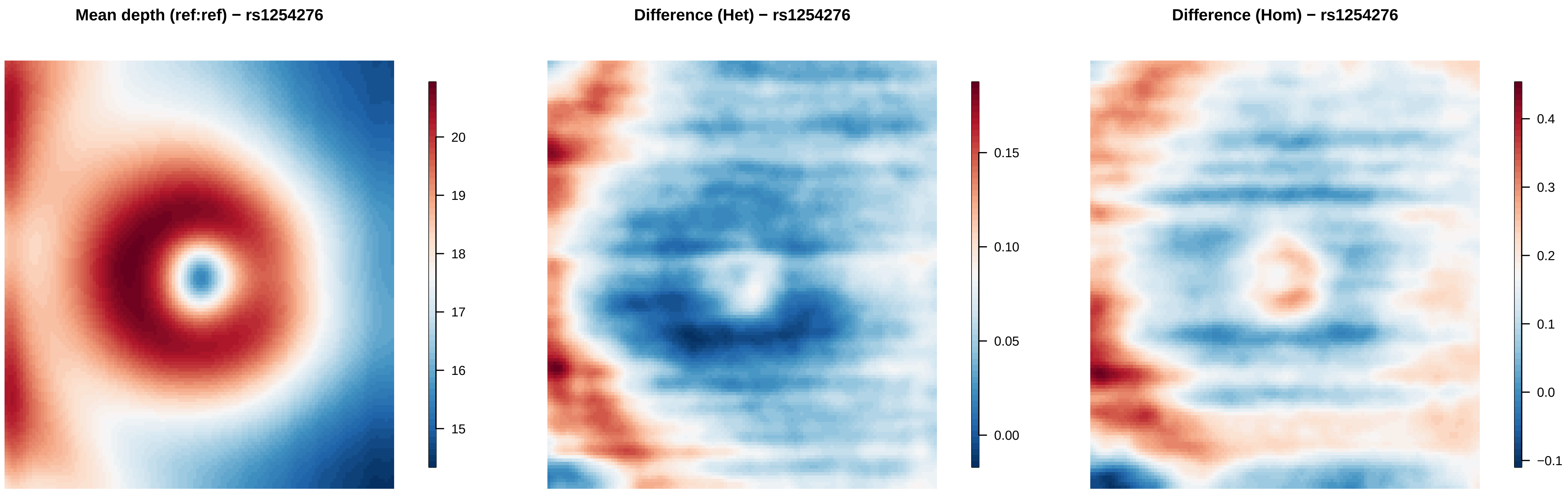
Mean depth (ref:ref) − rs1254276
20
19
18
17
16
15
Difference (Het) − rs1254276
0.15
0.10
0.05
0.00
Difference (Hom) − rs1254276
0.4
0.3
0.2
0.1
0.0
−0.1

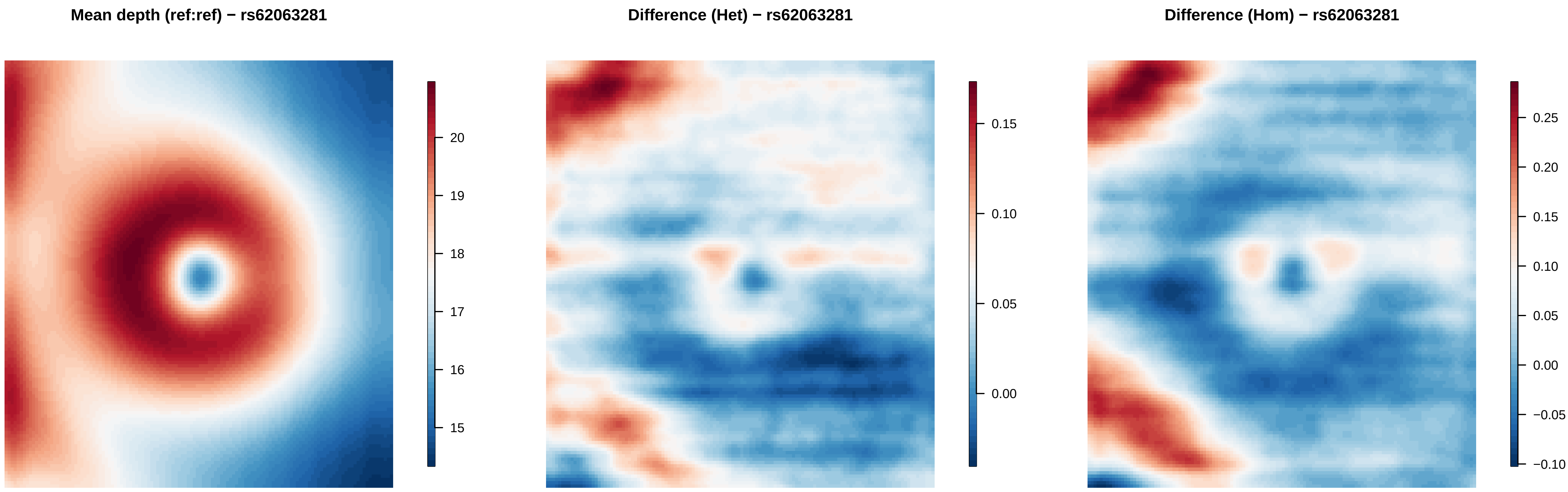
Mean depth (ref:ref) − rs62063281
20
19
18
17
16
15
Difference (Het) − rs62063281
0.15
0.10
0.05
0.00
Difference (Hom) − rs62063281
0.25
0.20
0.15
0.10
0.05
0.00
−0.05
−0.10

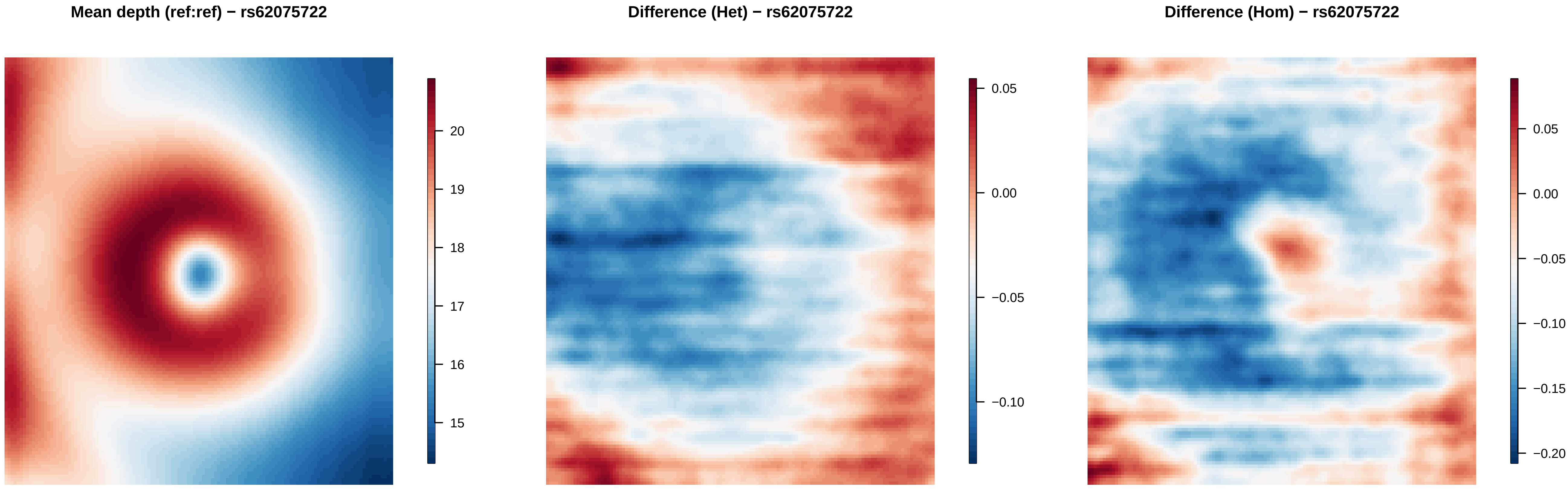

Mean depth (ref:ref) − rs62075722
20
19
18
17
16
15
Difference (Het) − rs62075722
0.05
0.00
−0.05
−0.10
Difference (Hom) − rs62075722
0.05
0.00
−0.05
−0.10
−0.15
−0.20

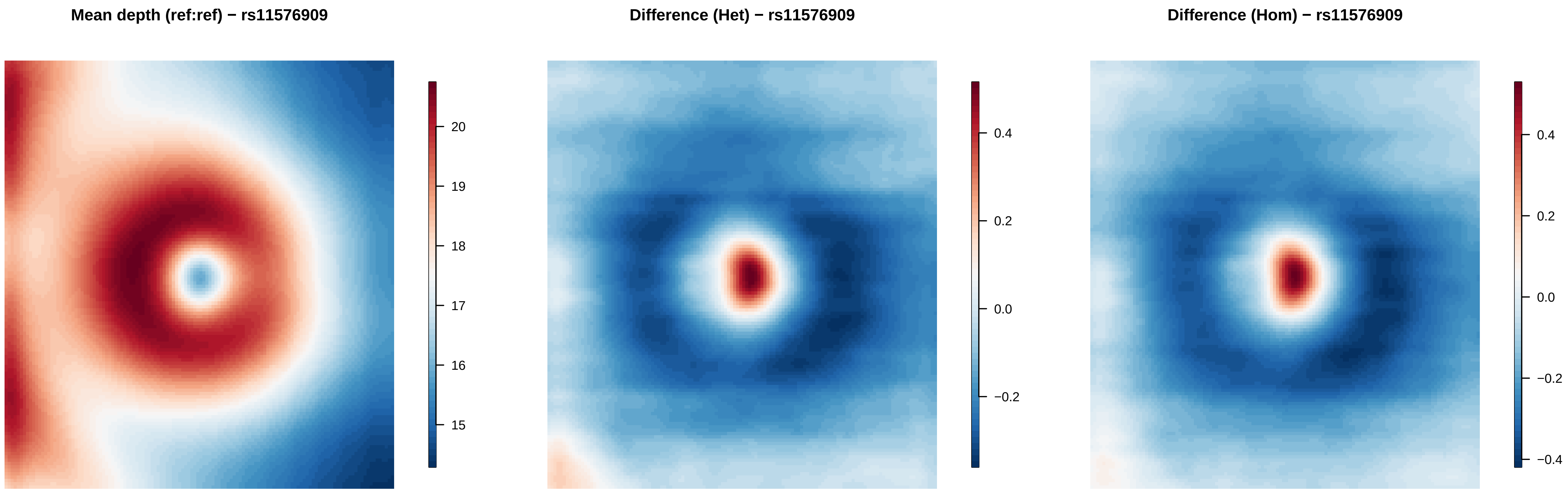
Mean depth (ref:ref) − rs11576909
20
19
18
17
16
15
Difference (Het) − rs11576909
0.4
0.2
0.0
−0.2
Difference (Hom) − rs11576909
0.4
0.2
0.0
−0.2
−0.4

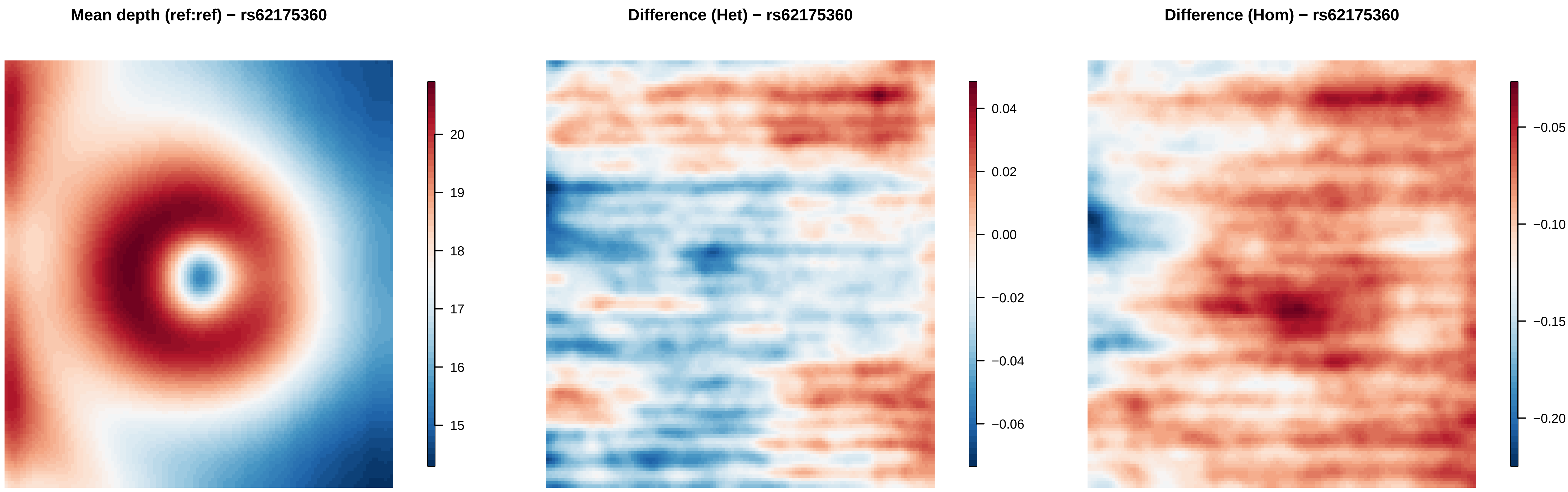
Mean depth (ref:ref) − rs62175360
20
19
18
17
16
15
Difference (Het) − rs62175360
0.04
0.02
0.00
−0.02
−0.04
−0.06
Difference (Hom) − rs62175360
−0.05
−0.10
−0.15
−0.20

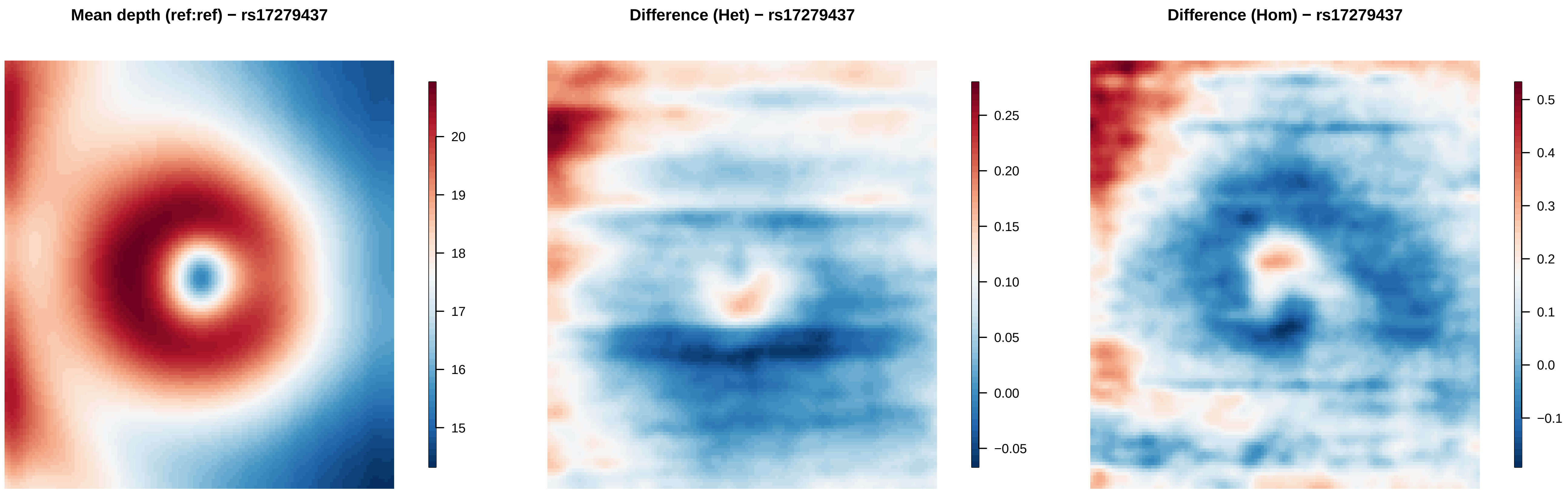
Mean depth (ref:ref) − rs17279437
20
19
18
17
16
15
Difference (Het) − rs17279437
0.25
0.20
0.15
0.10
0.05
0.00
−0.05
Difference (Hom) − rs17279437
0.5
0.4
0.3
0.2
0.1
0.0
−0.1

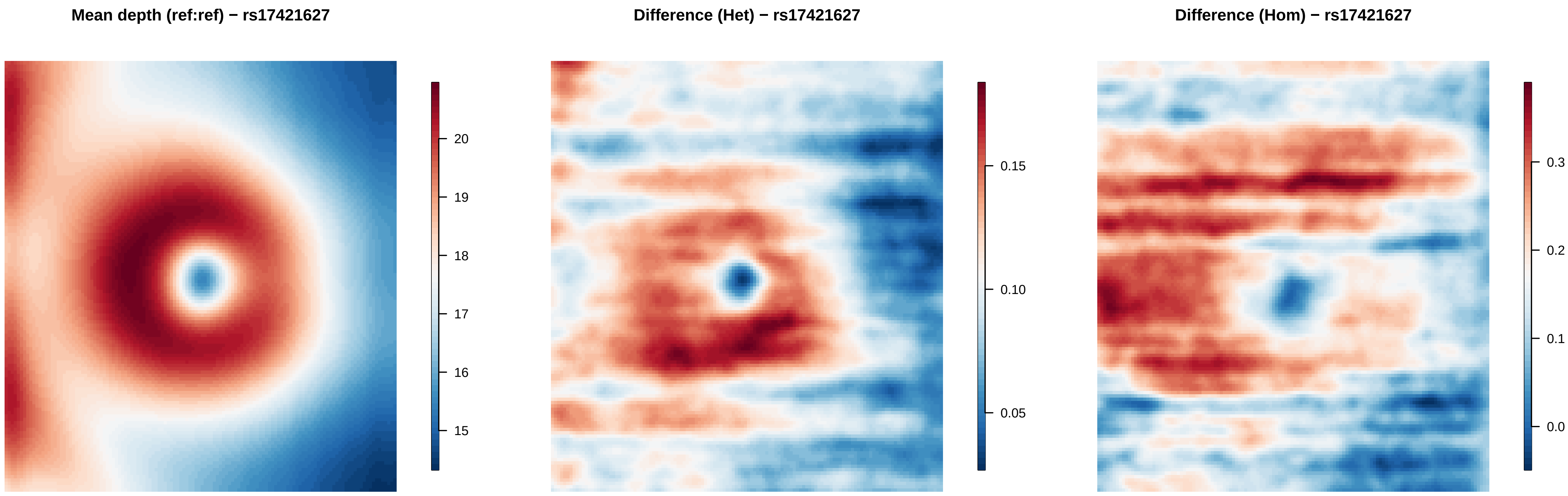
Mean depth (ref:ref) − rs17421627
20
19
18
17
16
15
Difference (Het) − rs17421627
0.15
0.10
0.05
Difference (Hom) − rs17421627
0.3
0.2
0.1
0.0

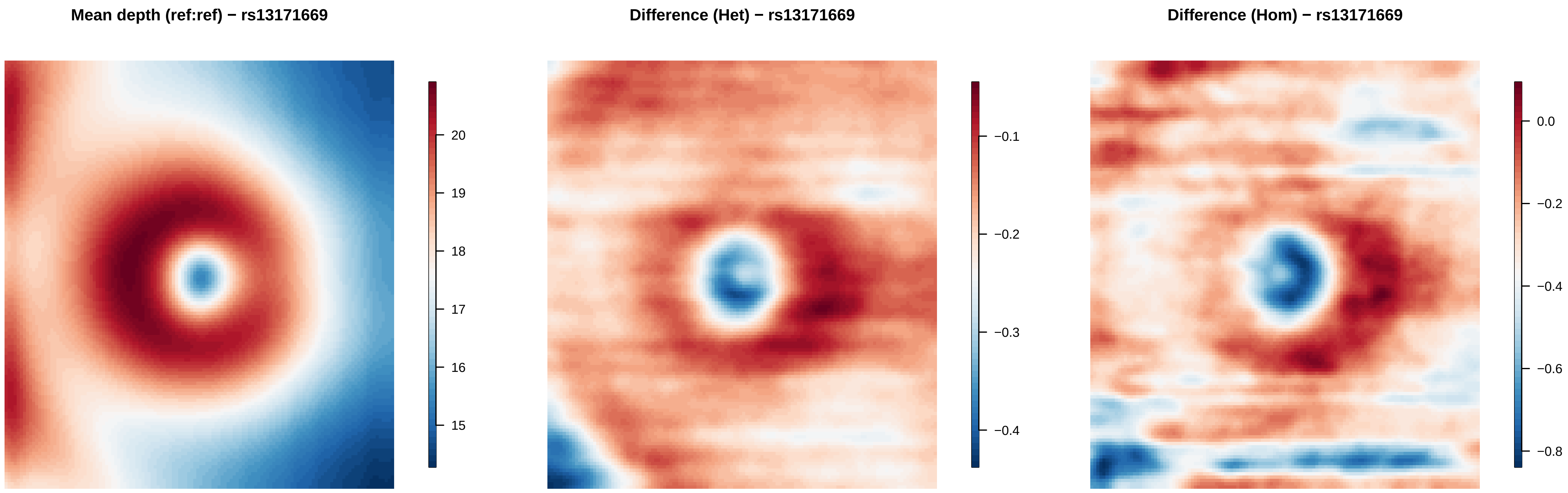
Mean depth (ref:ref) − rs13171669
20
19
18
17
16
15
Difference (Het) − rs13171669
−0.1
−0.2
−0.3
−0.4
Difference (Hom) − rs13171669
0.0
−0.2
−0.4
−0.6
−0.8

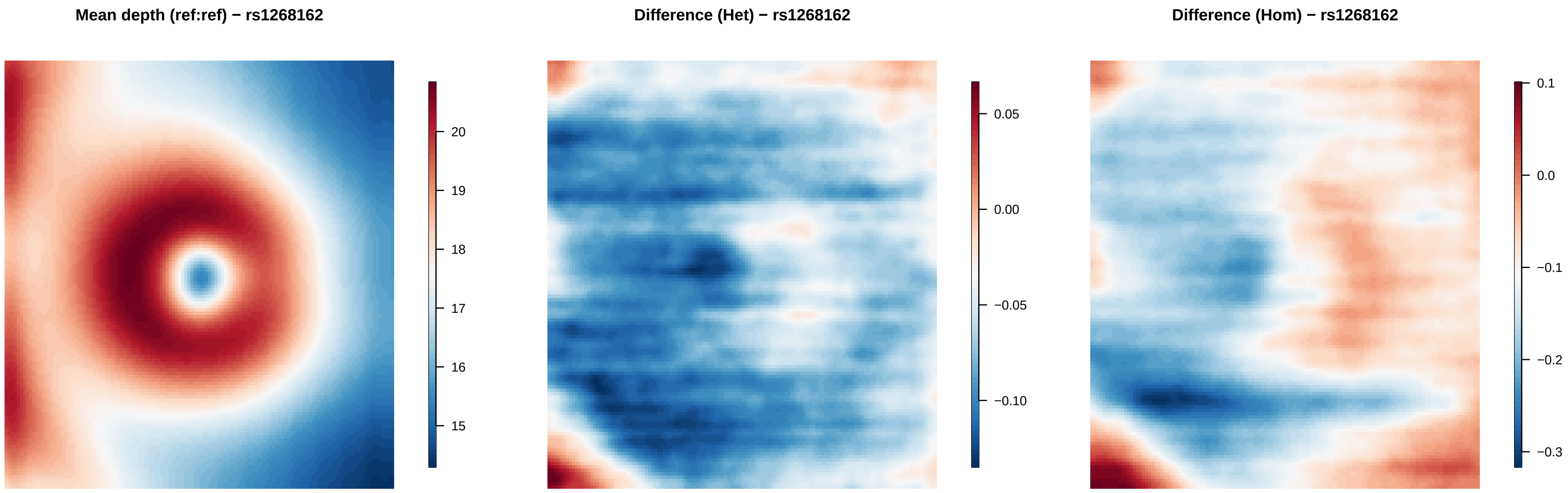

Mean depth (ref:ref) − rs1268162
20
19
18
17
16
15
Difference (Het) − rs1268162
0.05
0.00
−0.05
−0.10
Difference (Hom) − rs1268162
0.1
0.0
−0.1
−0.2
−0.3

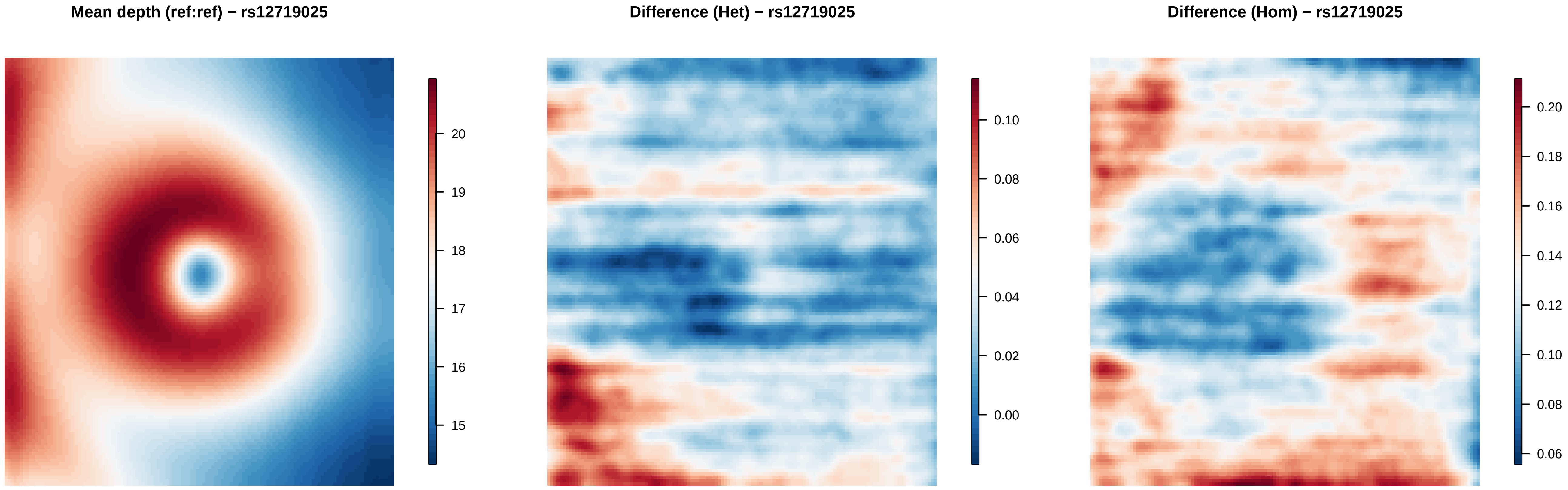
Mean depth (ref:ref) − rs12719025
20
19
18
17
16
15
Difference (Het) − rs12719025
0.10
0.08
0.06
0.04
0.02
0.00
Difference (Hom) − rs12719025
0.20
0.18
0.16
0.14
0.12
0.10
0.08
0.06

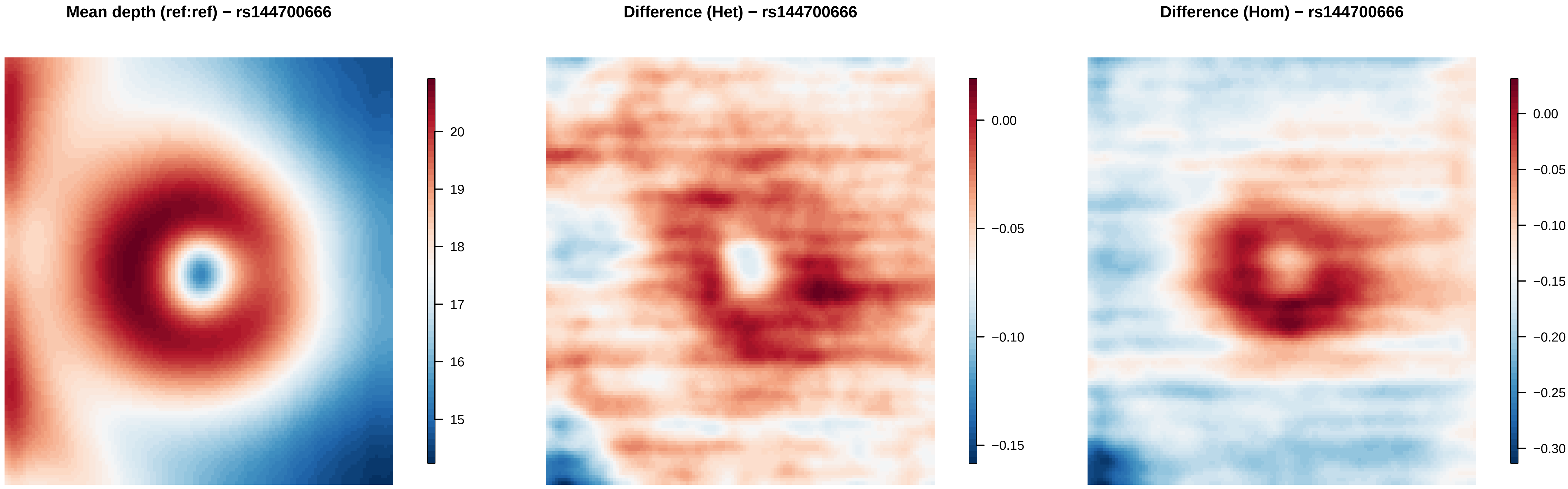
Mean depth (ref:ref) − rs144700666
20
19
18
17
16
15
Difference (Het) − rs144700666
0.00
−0.05
−0.10
−0.15
Difference (Hom) − rs144700666
0.00
−0.05
−0.10
−0.15
−0.20
−0.25
−0.30

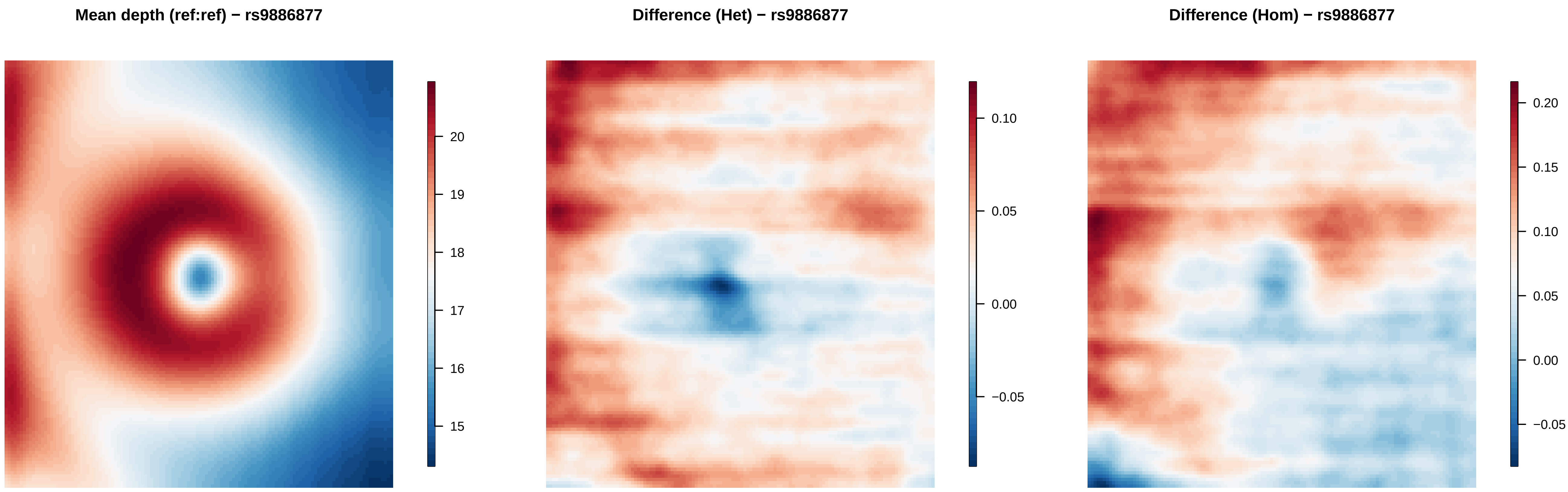
Mean depth (ref:ref) − rs9886877
20
19
18
17
16
15
Difference (Het) − rs9886877
0.10
0.05
0.00
−0.05
Difference (Hom) − rs9886877
0.20
0.15
0.10
0.05
0.00
−0.05

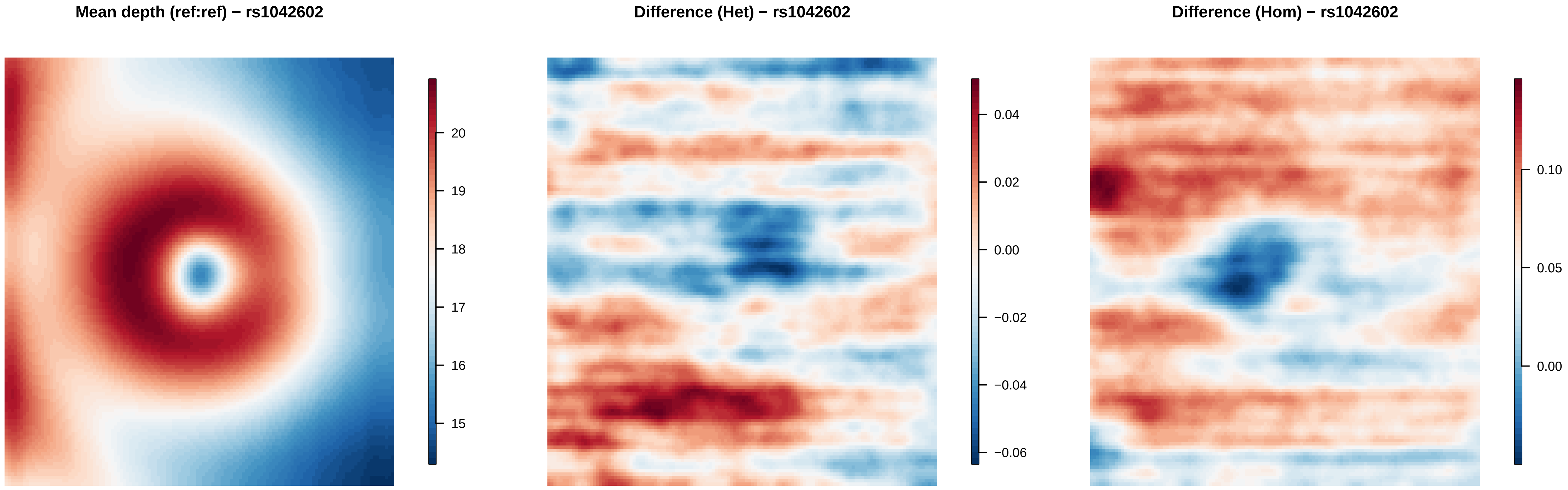

Mean depth (ref:ref) − rs1042602
20
19
18
17
16
15
Difference (Het) − rs1042602
0.04
0.02
0.00
−0.02
−0.04
−0.06
Difference (Hom) − rs1042602
0.10
0.05
0.00

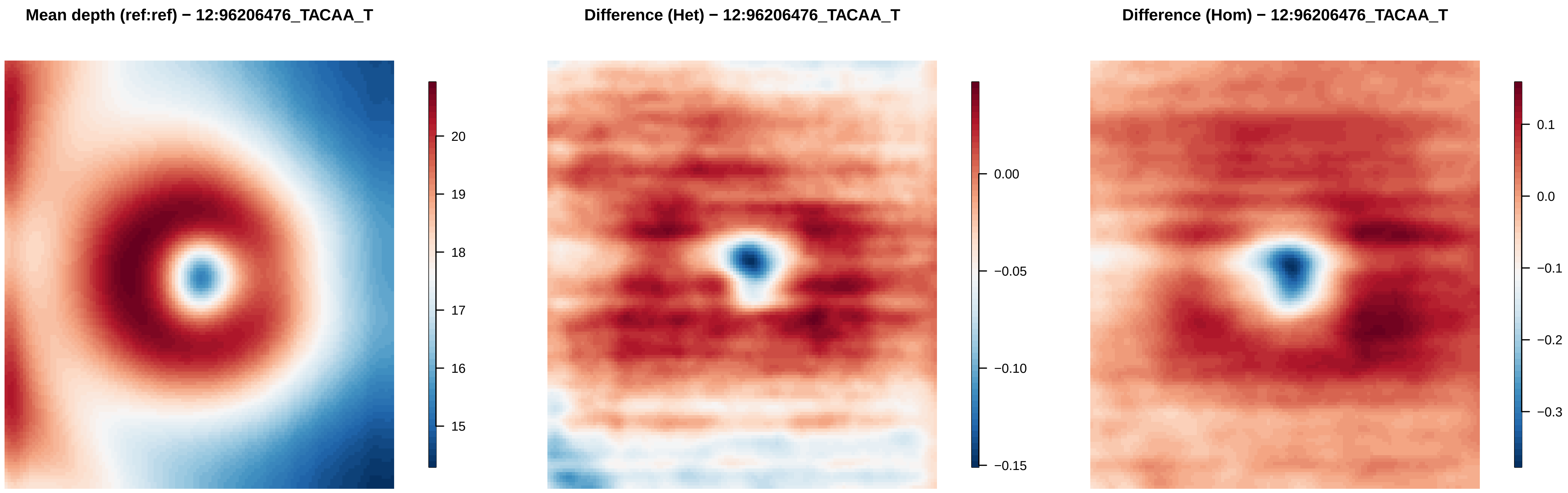

Mean depth (ref:ref) − 12:96206476_TACAA_T
20
19
18
17
16
15
Difference (Het) − 12:96206476_TACAA_T
0.00
−0.05
−0.10
−0.15
Difference (Hom) − 12:96206476_TACAA_T
0.1
0.0
−0.1
−0.2
−0.3

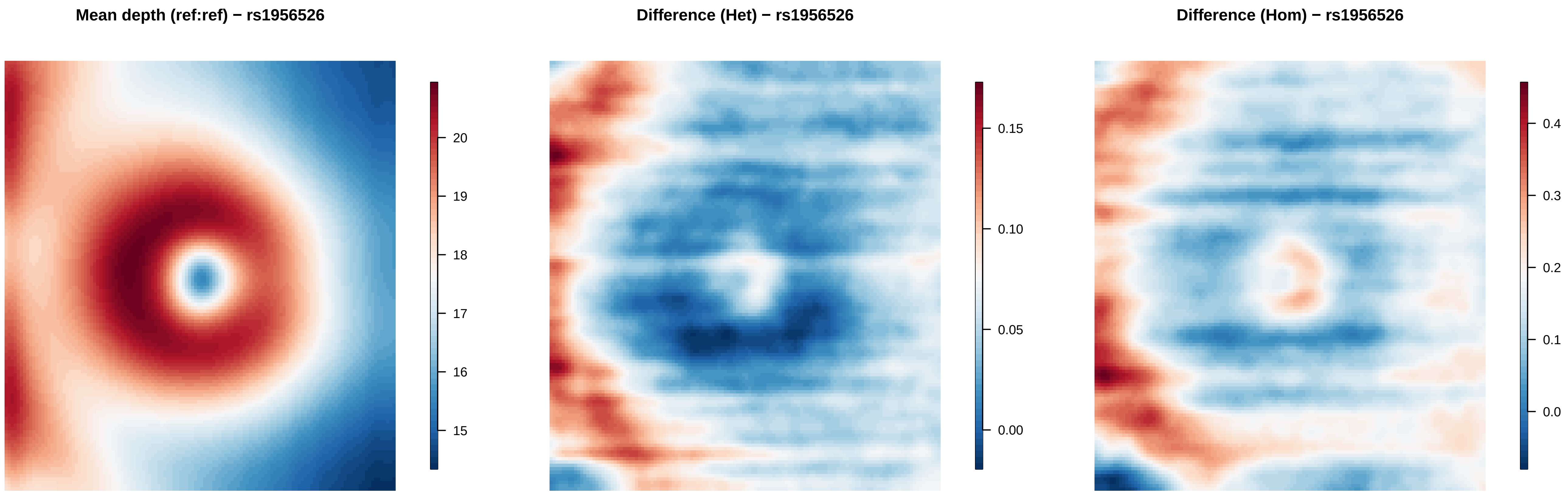

Mean depth (ref:ref) − rs1956526
20
19
18
17
16
15
Difference (Het) − rs1956526
0.15
0.10
0.05
0.00
Difference (Hom) − rs1956526
0.4
0.3
0.2
0.1
0.0

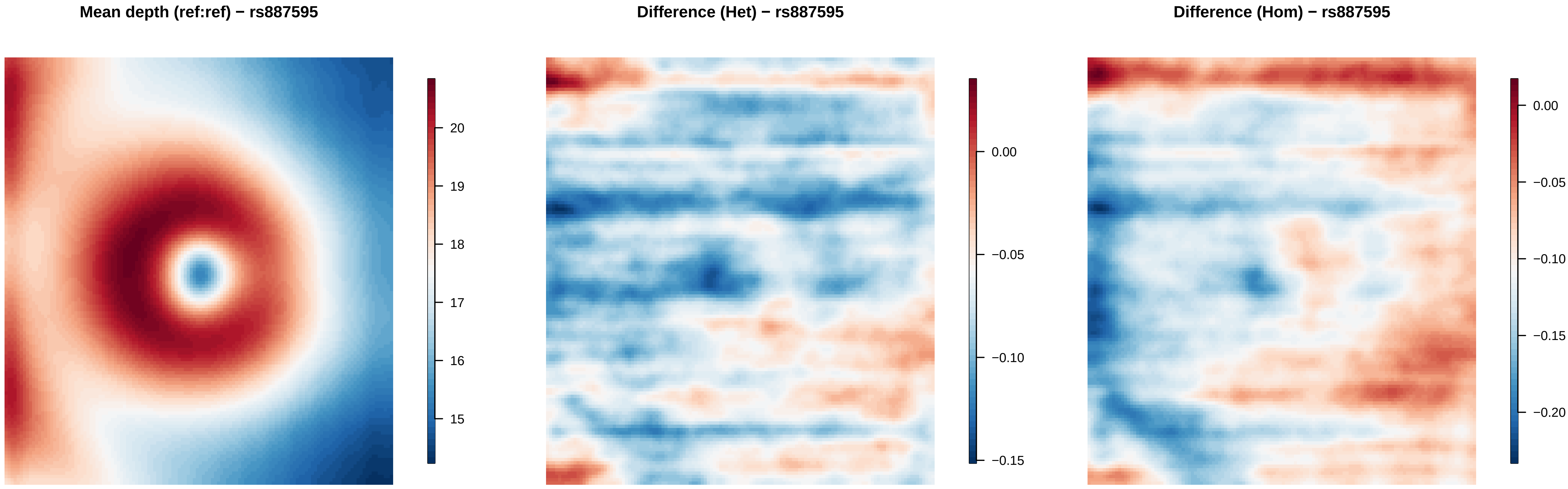
Mean depth (ref:ref) − rs887595
20
19
18
17
16
15
Difference (Het) − rs887595
0.00
−0.05
−0.10
−0.15
Difference (Hom) − rs887595
0.00
−0.05
−0.10
−0.15
−0.20

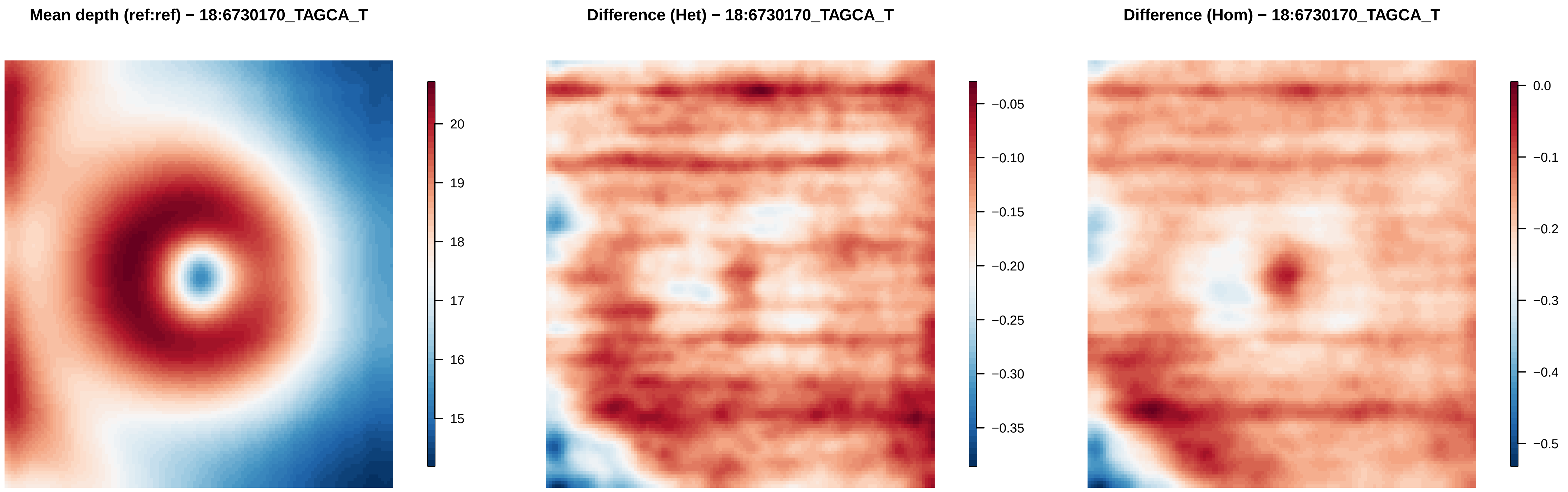
Mean depth (ref:ref) − 18:6730170_TAGCA_T
20
19
18
17
16
15
Difference (Het) − 18:6730170_TAGCA_T
−0.05
−0.10
−0.15
−0.20
−0.25
−0.30
−0.35
Difference (Hom) − 18:6730170_TAGCA_T
0.0
−0.1
−0.2
−0.3
−0.4
−0.5

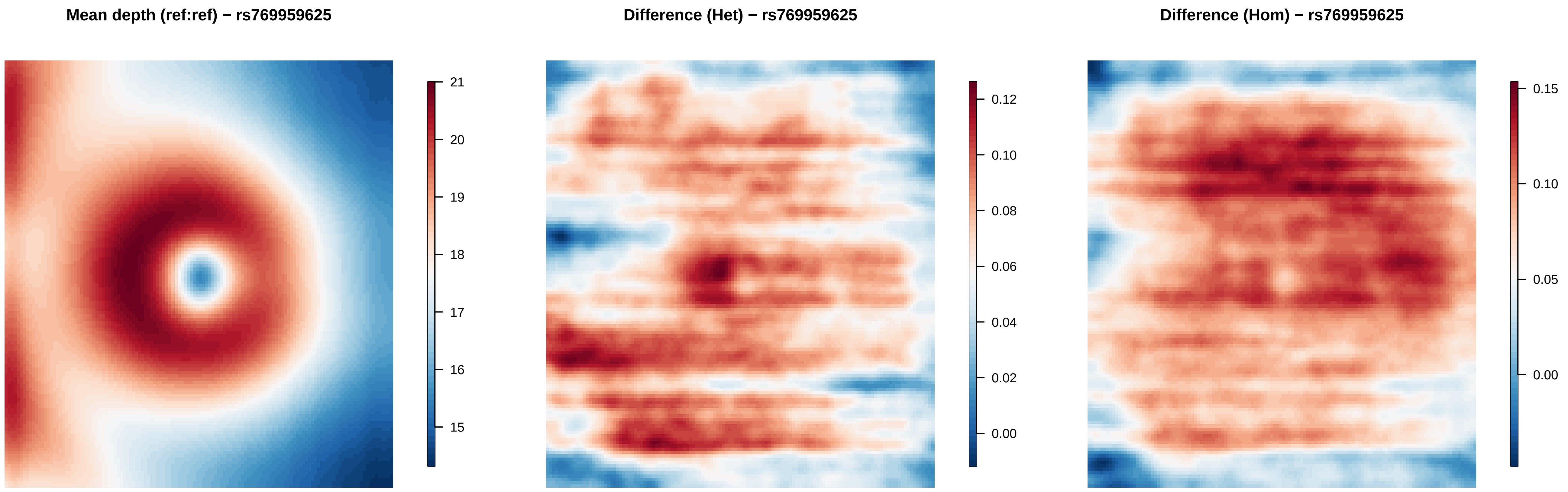
Mean depth (ref:ref) − rs769959625
21
20
19
18
17
16
15
Difference (Het) − rs769959625
0.12
0.10
0.08
0.06
0.04
0.02
0.00
Difference (Hom) − rs769959625
0.15
0.10
0.05
0.00

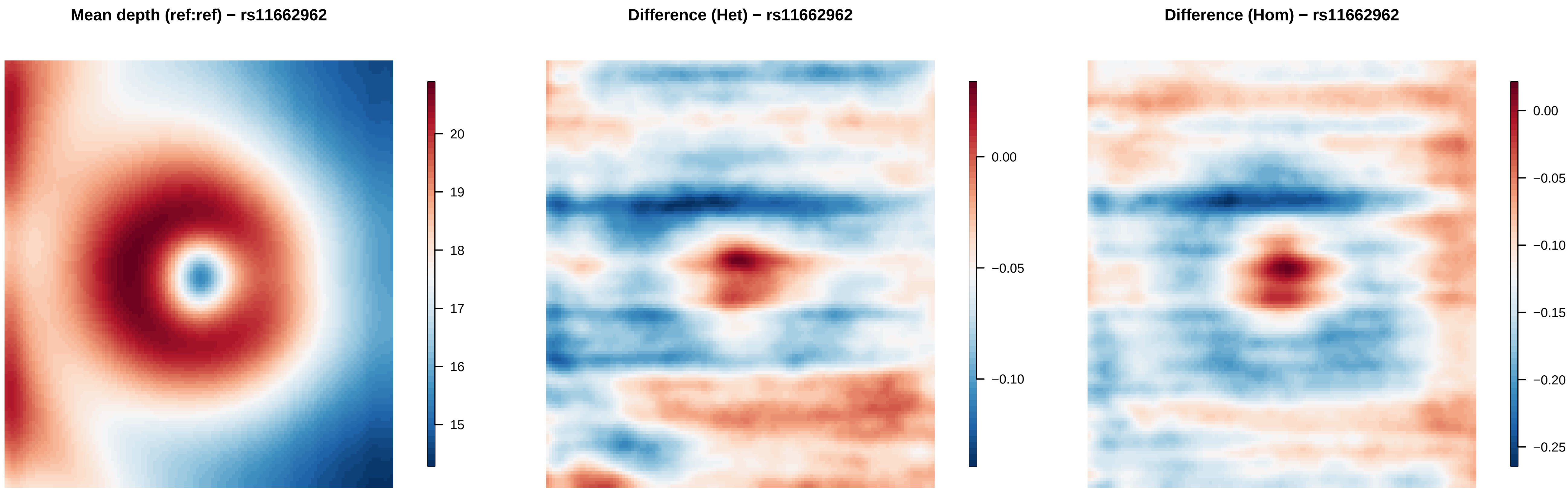
Mean depth (ref:ref) − rs11662962
20
19
18
17
16
15
Difference (Het) − rs11662962
0.00
−0.05
−0.10
Difference (Hom) − rs11662962
0.00
−0.05
−0.10
−0.15
−0.20
−0.25

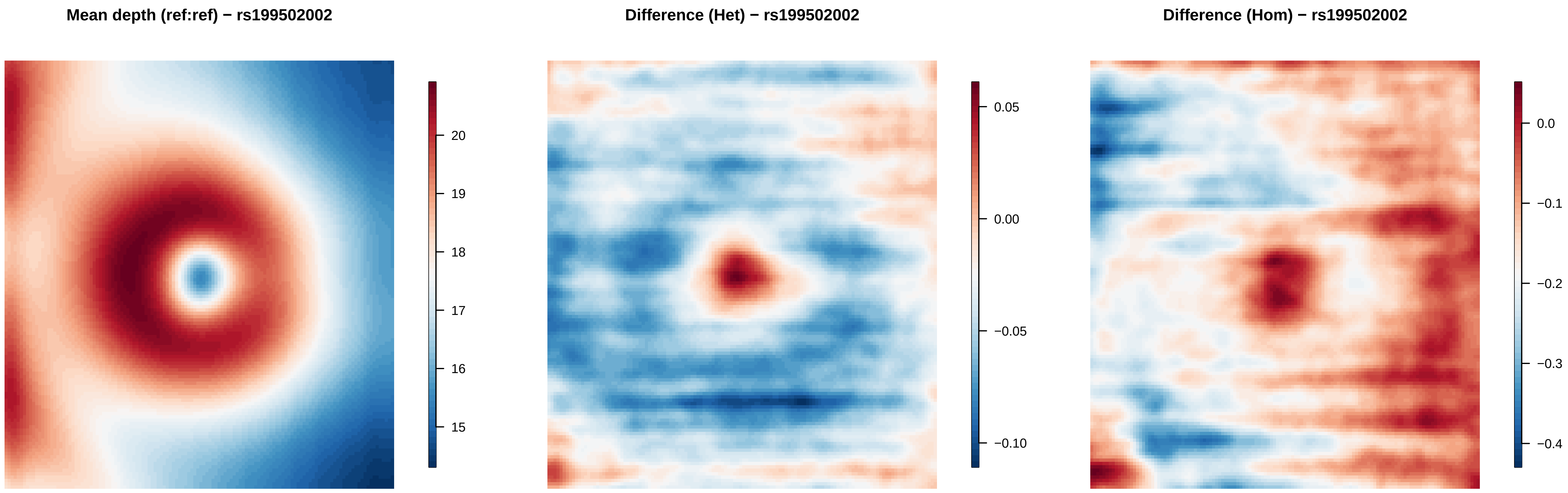
Mean depth (ref:ref) − rs199502002
20
19
18
17
16
15
Difference (Het) − rs199502002
0.05
0.00
−0.05
−0.10
Difference (Hom) − rs199502002
0.0
−0.1
−0.2
−0.3
−0.4

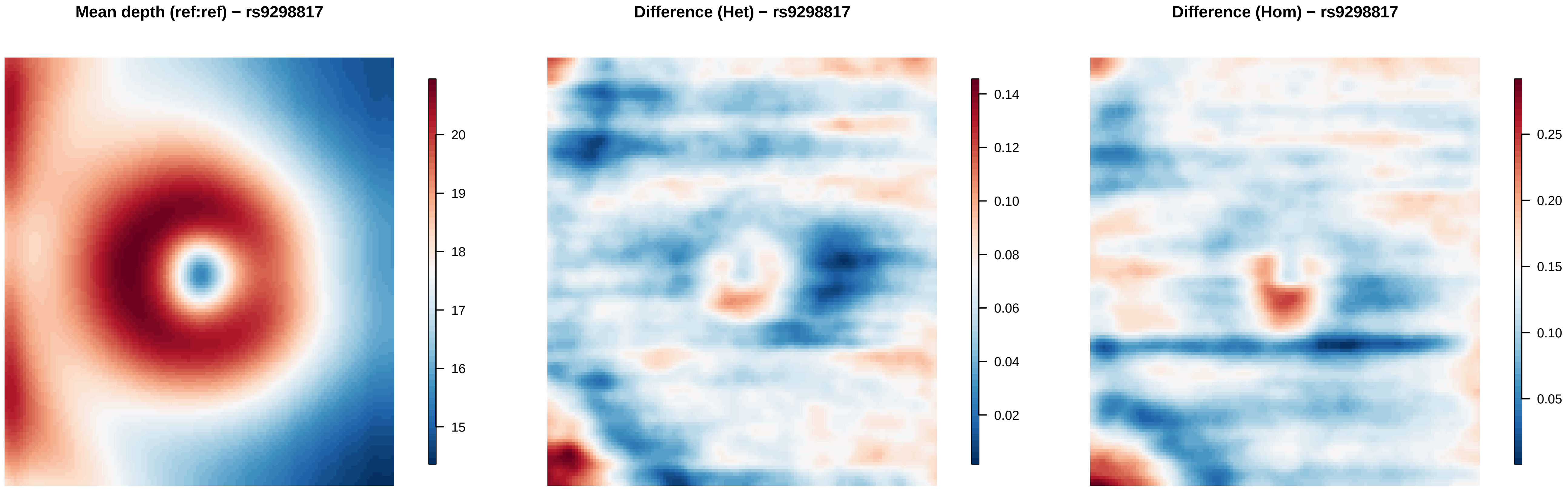
Mean depth (ref:ref) − rs9298817
20
19
18
17
16
15
Difference (Het) − rs9298817
0.14
0.12
0.10
0.08
0.06
0.04
0.02
Difference (Hom) − rs9298817
0.25
0.20
0.15
0.10
0.05

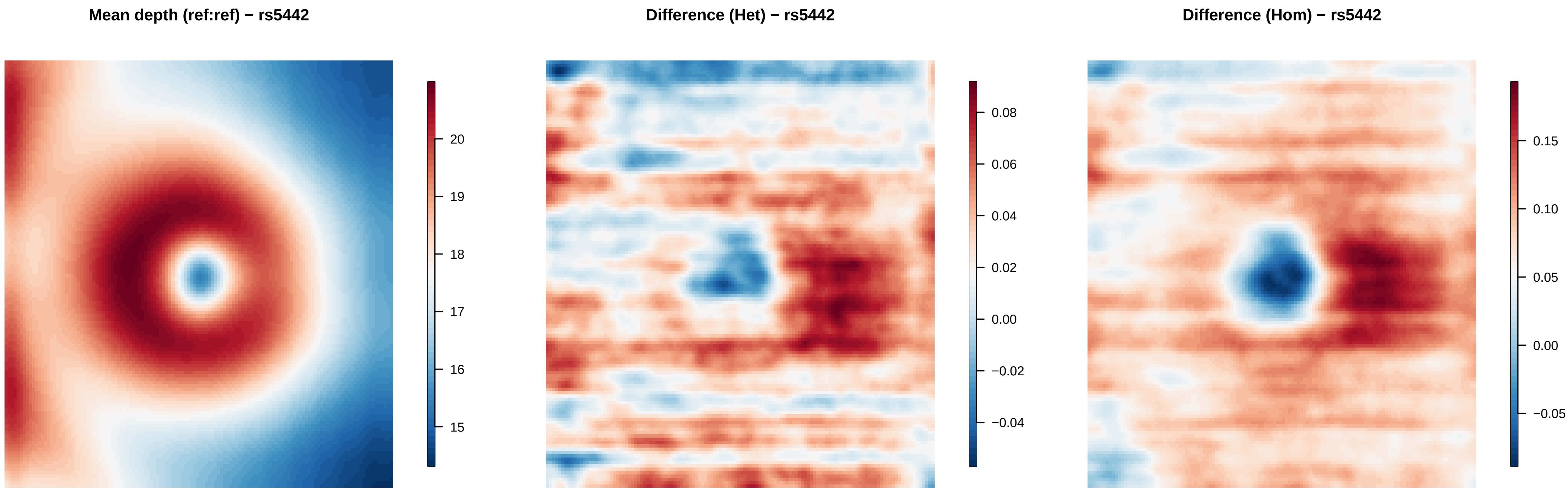
Mean depth (ref:ref) − rs5442
20
19
18
17
16
15
Difference (Het) − rs5442
0.08
0.06
0.04
0.02
0.00
−0.02
−0.04
Difference (Hom) − rs5442
0.15
0.10
0.05
0.00
−0.05

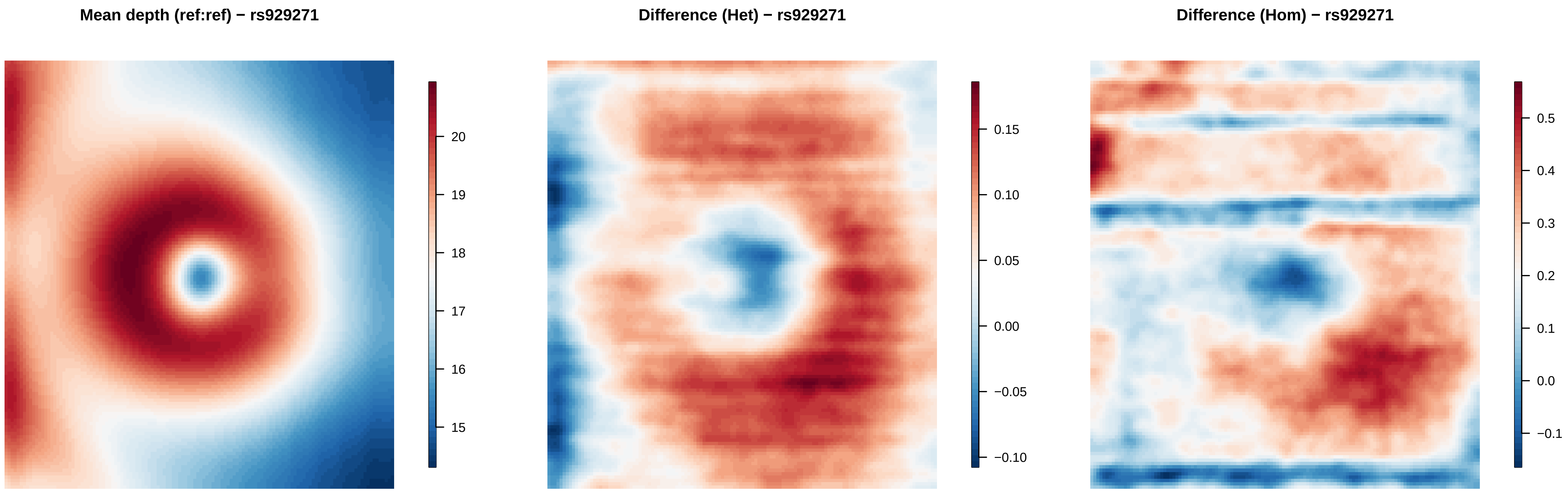
Mean depth (ref:ref) − rs929271
20
19
18
17
16
15
Difference (Het) − rs929271
0.15
0.10
0.05
0.00
−0.05
−0.10
Difference (Hom) − rs929271
0.5
0.4
0.3
0.2
0.1
0.0
−0.1

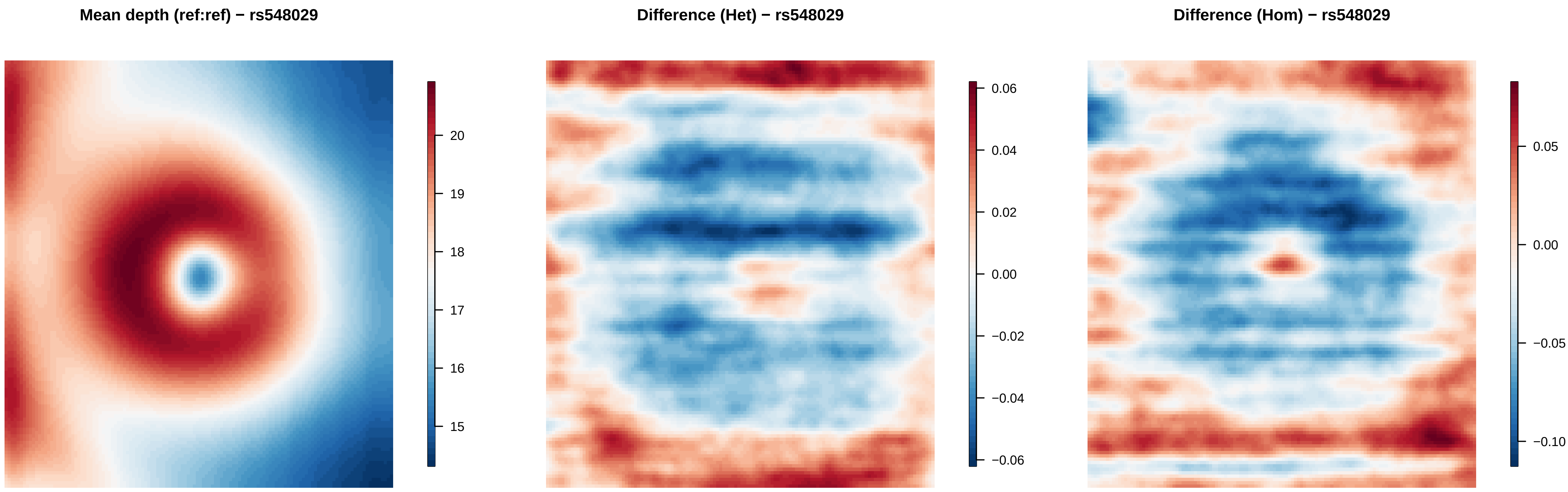
Mean depth (ref:ref) − rs548029
20
19
18
17
16
15
Difference (Het) − rs548029
0.06
0.04
0.02
0.00
−0.02
−0.04
−0.06
Difference (Hom) − rs548029
0.05
0.00
−0.05
−0.10

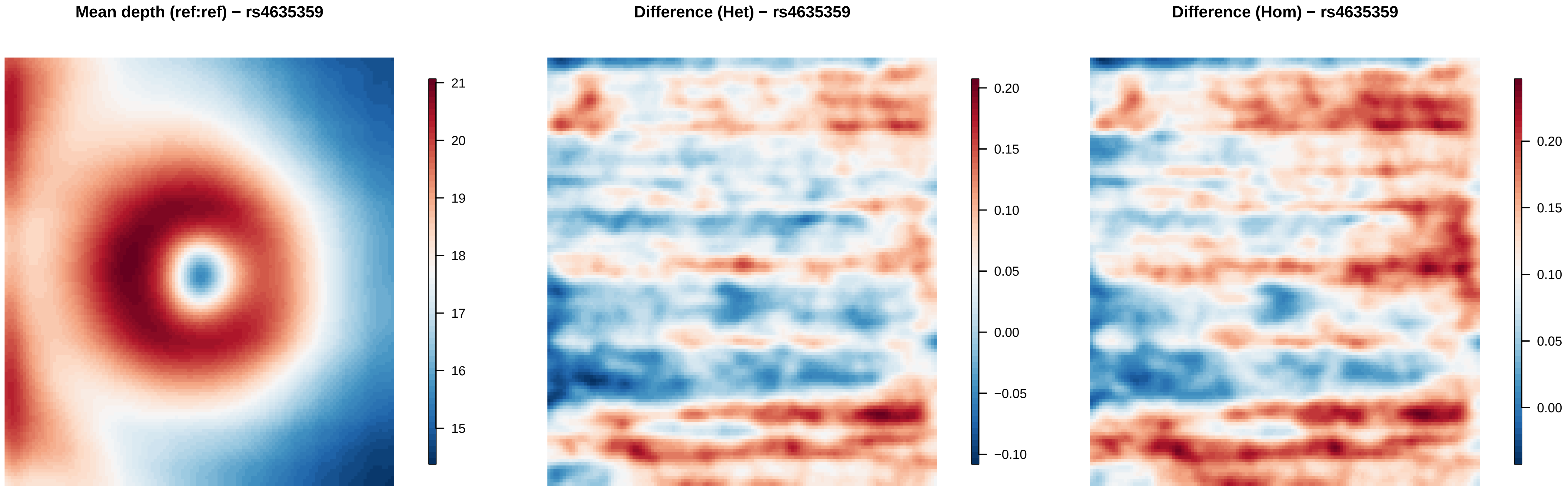
Mean depth (ref:ref) − rs4635359
21
20
19
18
17
16
15
Difference (Het) − rs4635359
0.20
0.15
0.10
0.05
0.00
−0.05
−0.10
Difference (Hom) − rs4635359
0.20
0.15
0.10
0.05
0.00

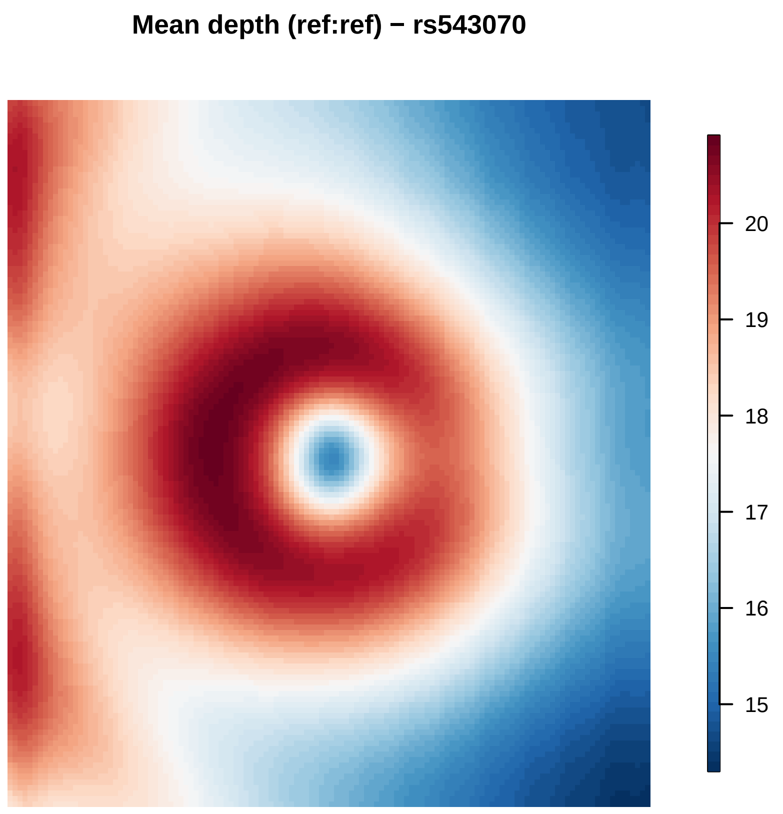

Mean depth (ref:ref) − rs543070
20
19
18
17
16
15

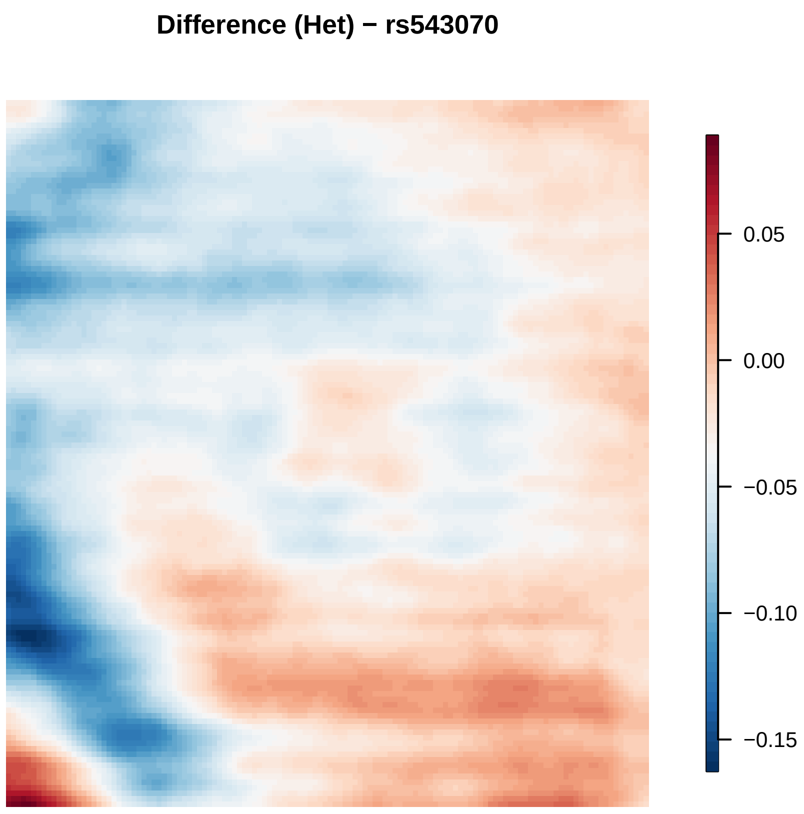

Difference (Het) − rs543070
0.05
0.00
−0.05
−0.10
−0.15

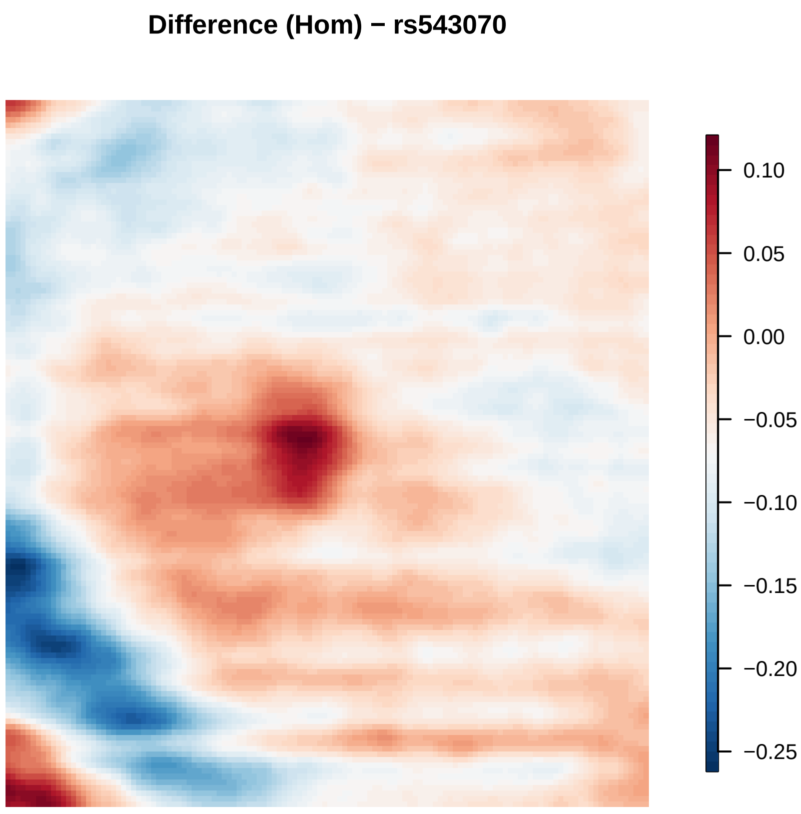

Difference (Hom) − rs543070
0.10
0.05
0.00
−0.05
−0.10
−0.15
−0.20
−0.25

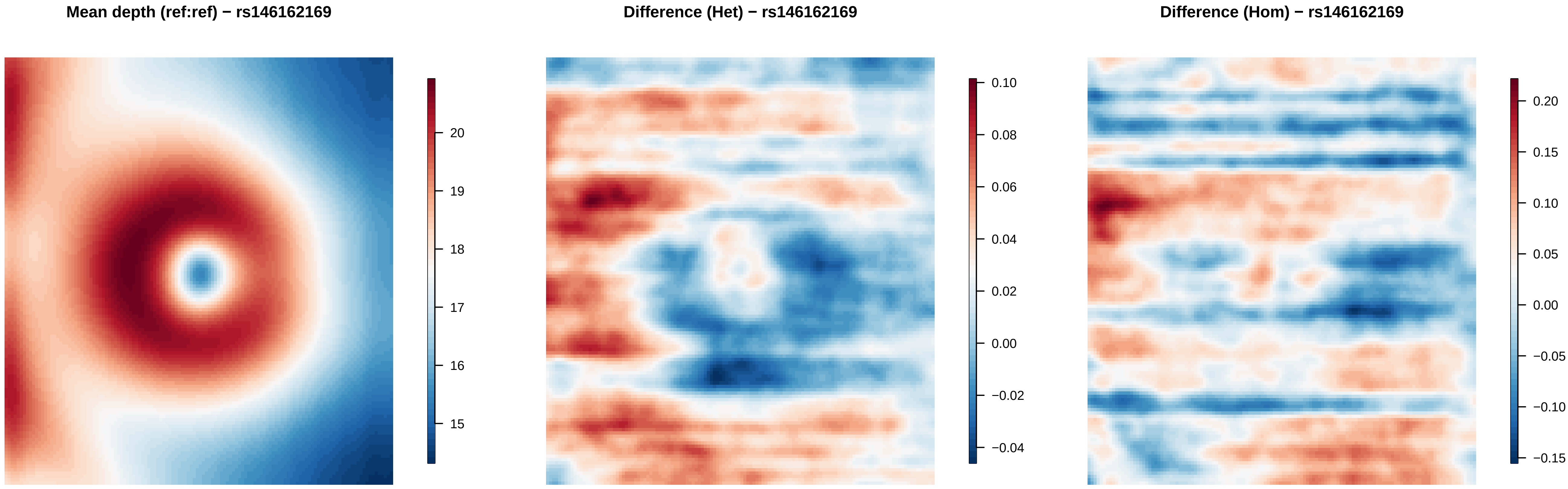
Mean depth (ref:ref) − rs146162169
20
19
18
17
16
15
Difference (Het) − rs146162169
0.10
0.08
0.06
0.04
0.02
0.00
−0.02
−0.04
Difference (Hom) − rs146162169
0.20
0.15
0.10
0.05
0.00
−0.05
−0.10
−0.15

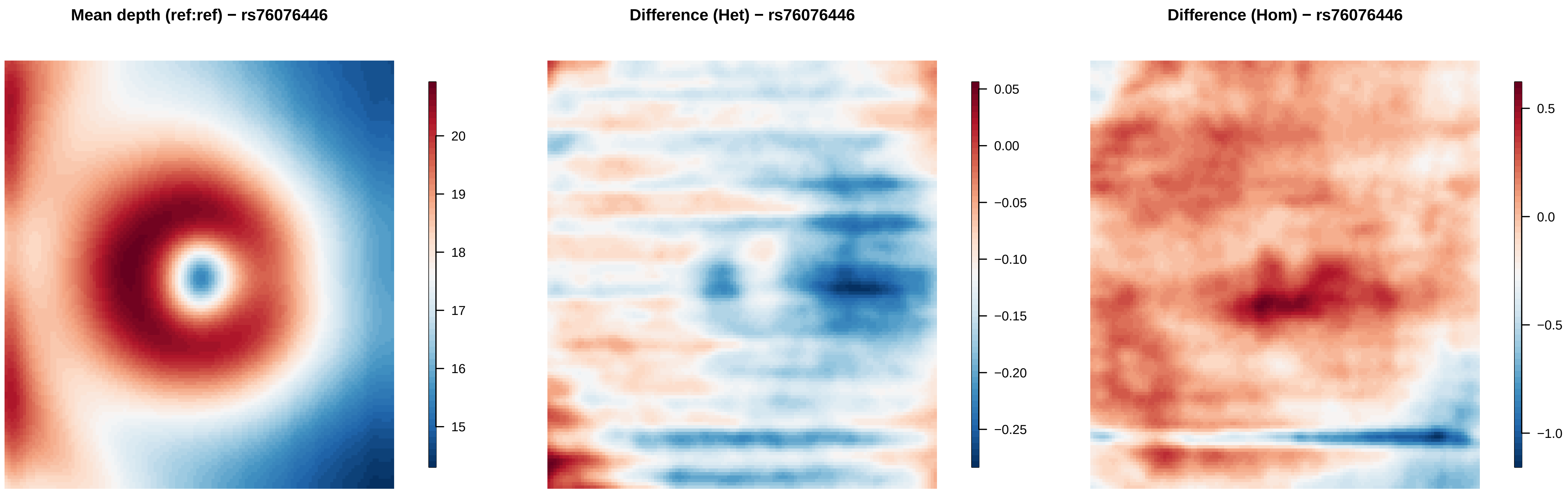
Mean depth (ref:ref) − rs76076446
20
19
18
17
16
15
Difference (Het) − rs76076446
0.05
0.00
−0.05
−0.10
−0.15
−0.20
−0.25
Difference (Hom) − rs76076446
0.5
0.0
−0.5
−1.0

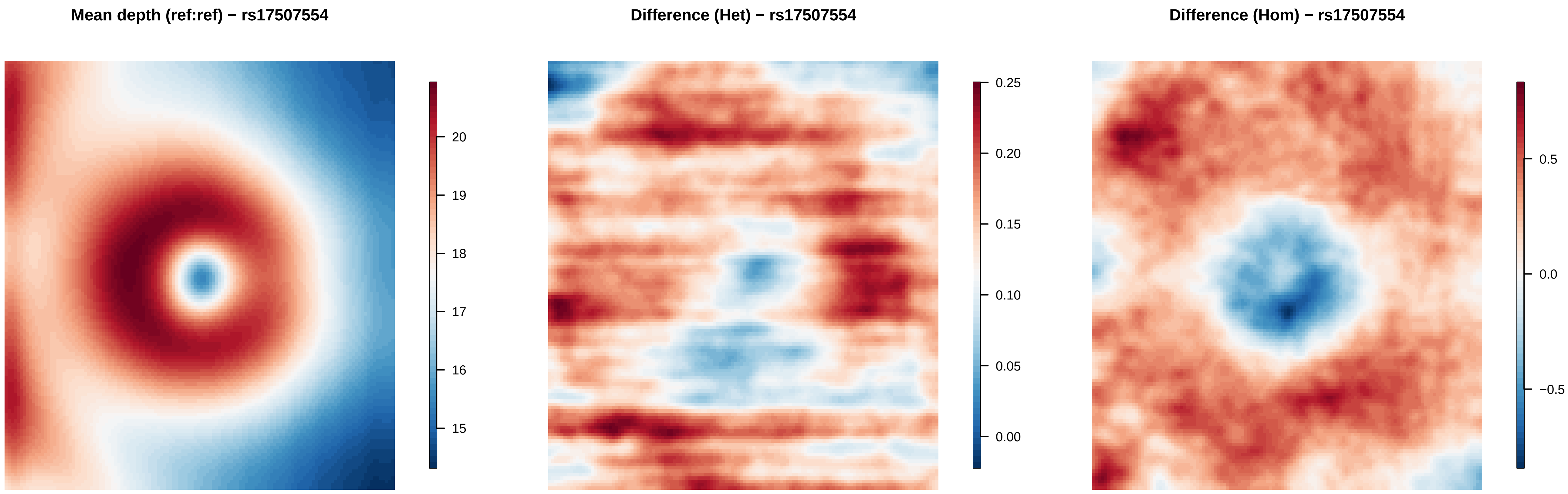
Mean depth (ref:ref) − rs17507554
20
19
18
17
16
15
Difference (Het) − rs17507554
0.25
0.20
0.15
0.10
0.05
0.00
Difference (Hom) − rs17507554
0.5
0.0
−0.5

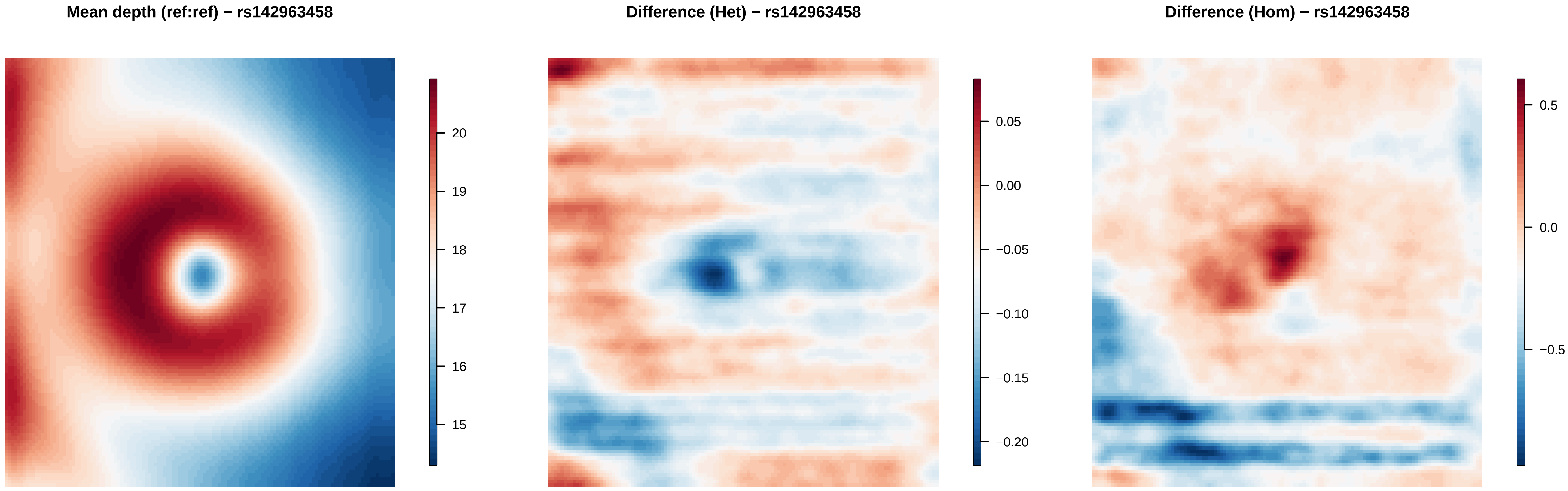
Mean depth (ref:ref) − rs142963458
20
19
18
17
16
15
Difference (Het) − rs142963458
0.05
0.00
−0.05
−0.10
−0.15
−0.20
Difference (Hom) − rs142963458
0.5
0.0
−0.5

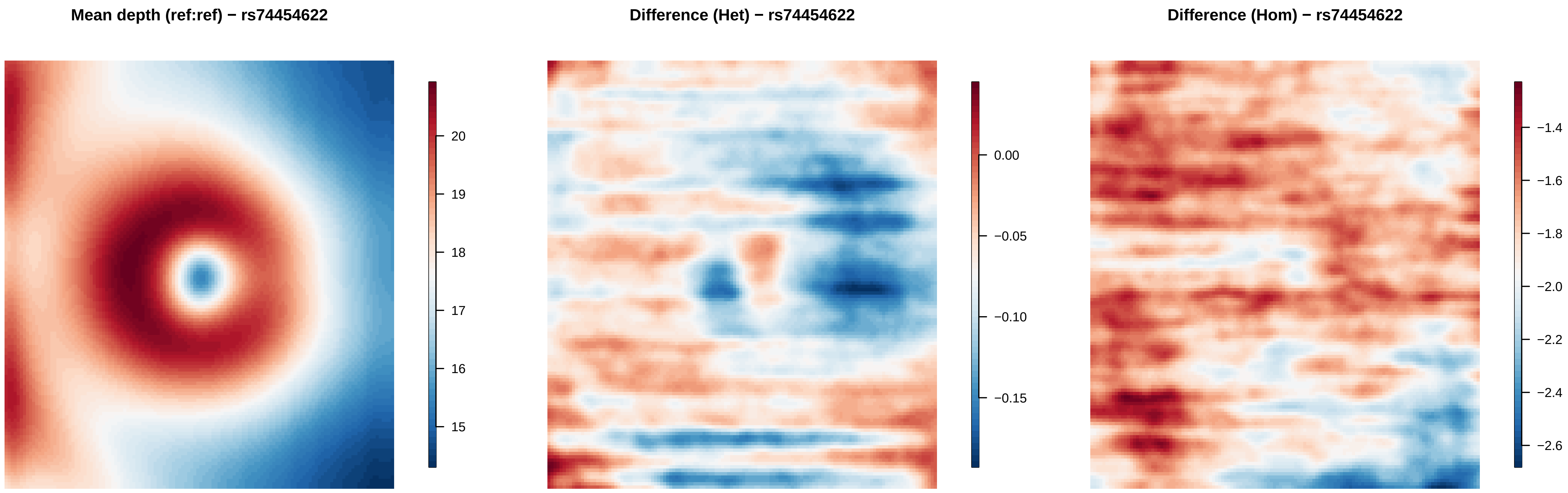
Mean depth (ref:ref) − rs74454622
20
19
18
17
16
15
Difference (Het) − rs74454622
0.00
−0.05
−0.10
−0.15
Difference (Hom) − rs74454622
−1.4
−1.6
−1.8
−2.0
−2.2
−2.4
−2.6

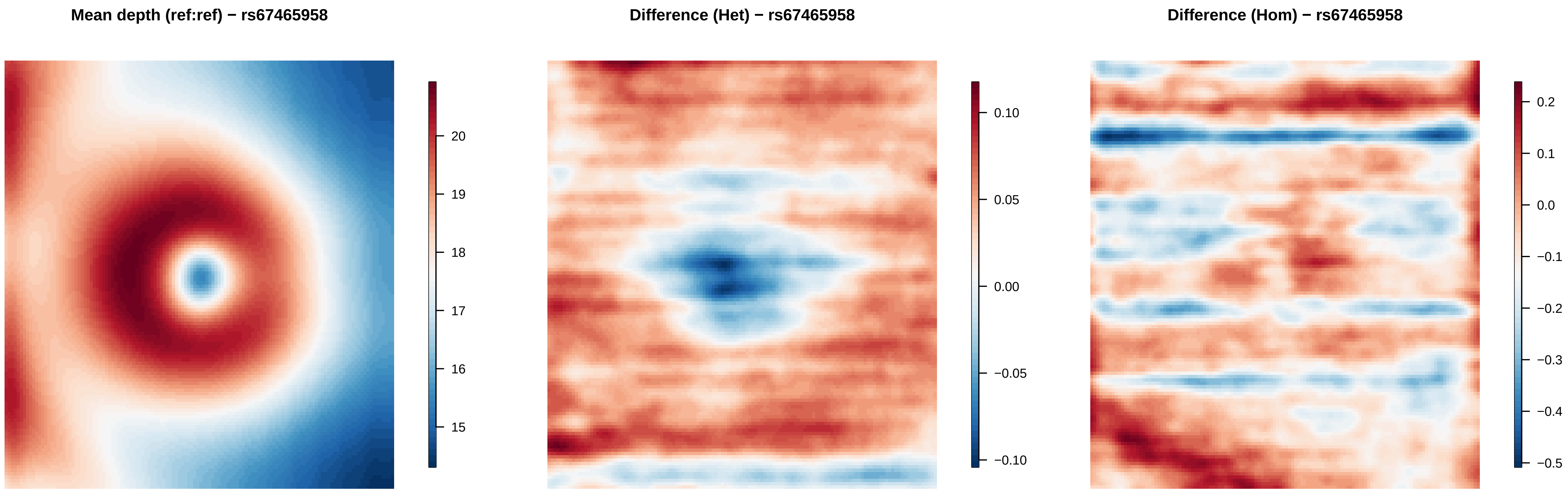
Mean depth (ref:ref) − rs67465958
20
19
18
17
16
15
Difference (Het) − rs67465958
0.10
0.05
0.00
−0.05
−0.10
Difference (Hom) − rs67465958
0.2
0.1
0.0
−0.1
−0.2
−0.3
−0.4
−0.5

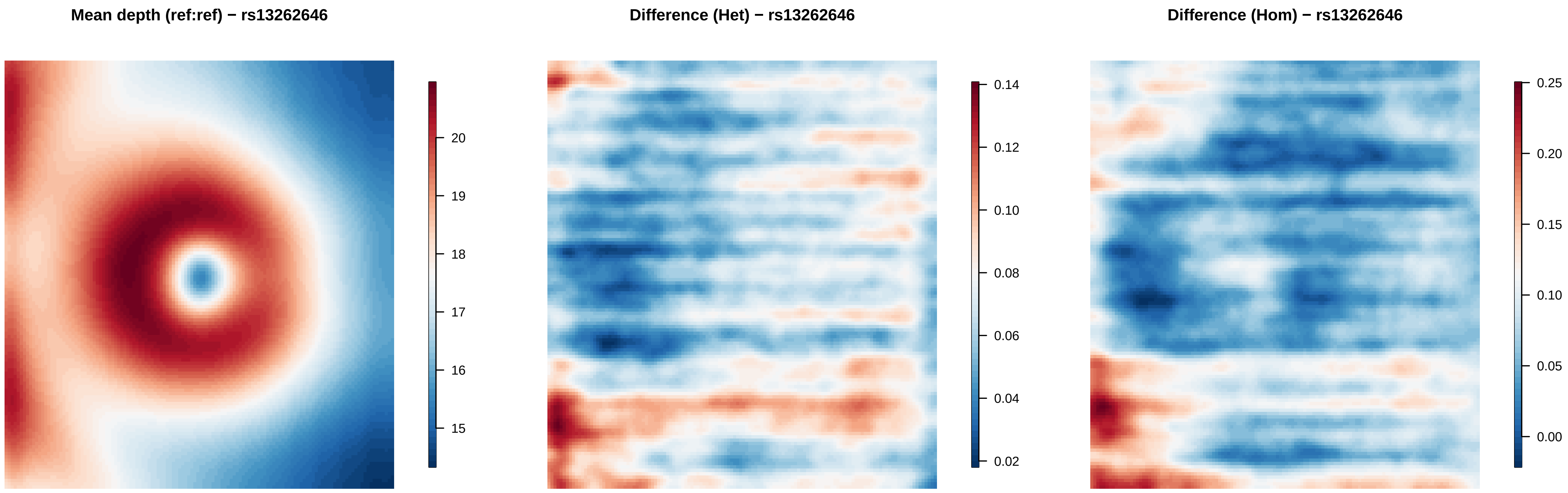
Mean depth (ref:ref) − rs13262646
20
19
18
17
16
15
Difference (Het) − rs13262646
0.14
0.12
0.10
0.08
0.06
0.04
0.02
Difference (Hom) − rs13262646
0.25
0.20
0.15
0.10
0.05
0.00

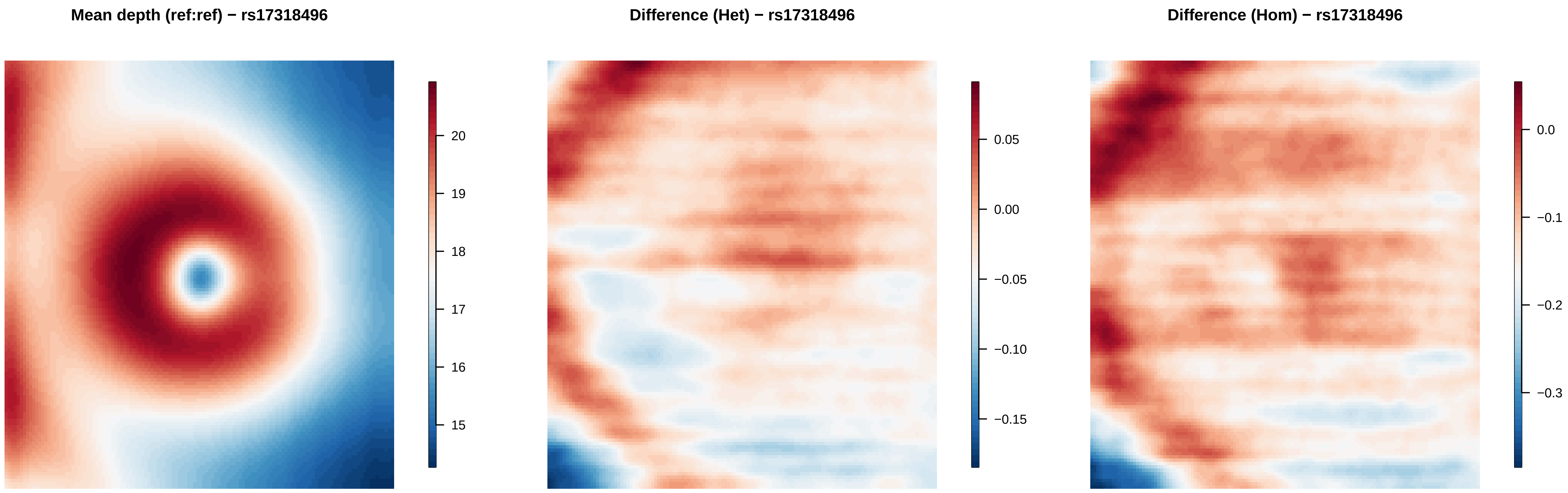
Mean depth (ref:ref) − rs17318496
20
19
18
17
16
15
Difference (Het) − rs17318496
0.05
0.00
−0.05
−0.10
−0.15
Difference (Hom) − rs17318496
0.0
−0.1
−0.2
−0.3

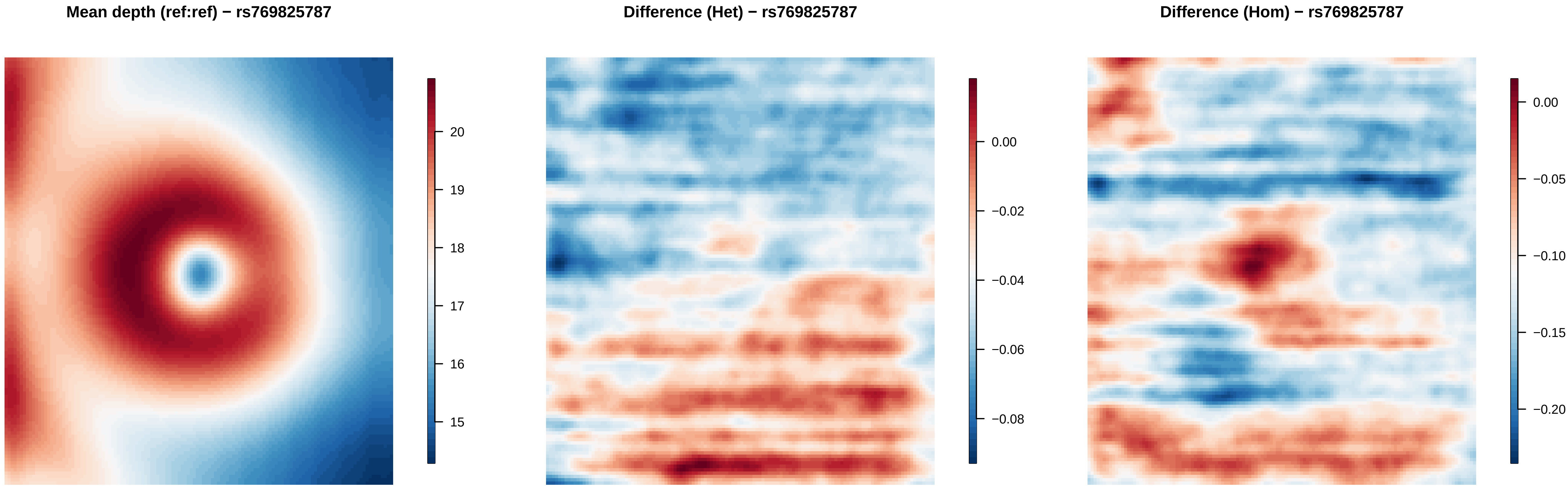
Mean depth (ref:ref) − rs769825787
20
19
18
17
16
15
Difference (Het) − rs769825787
0.00
−0.02
−0.04
−0.06
−0.08
Difference (Hom) − rs769825787
0.00
−0.05
−0.10
−0.15
−0.20

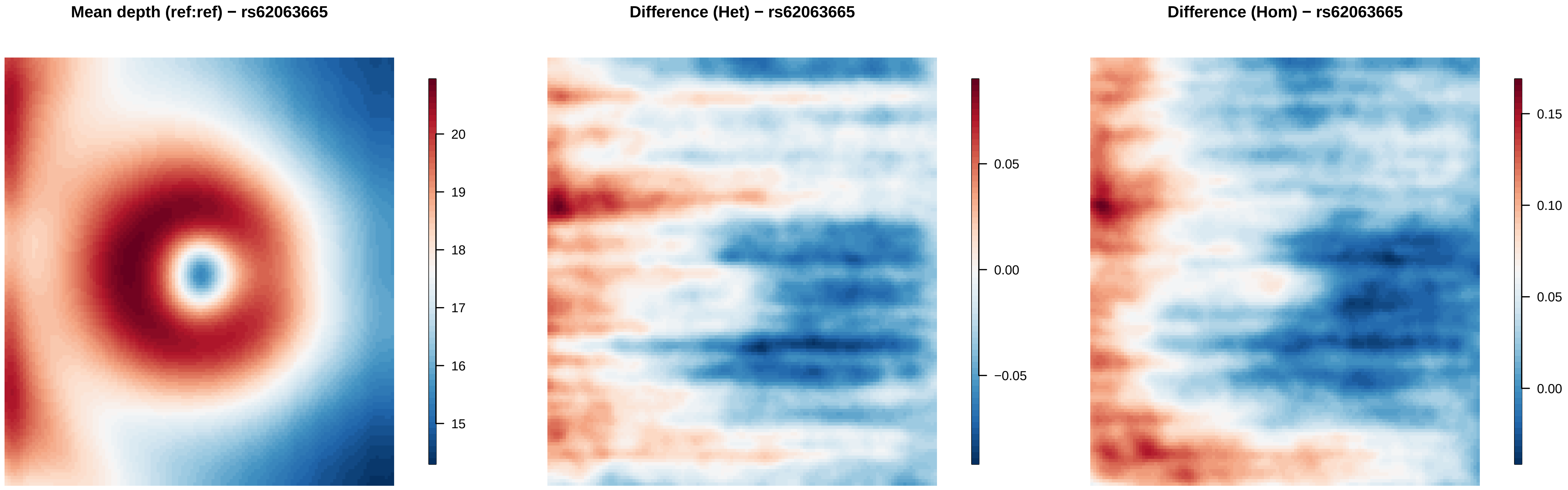
Mean depth (ref:ref) − rs62063665
20
19
18
17
16
15
Difference (Het) − rs62063665
0.05
0.00
−0.05
Difference (Hom) − rs62063665
0.15
0.10
0.05
0.00

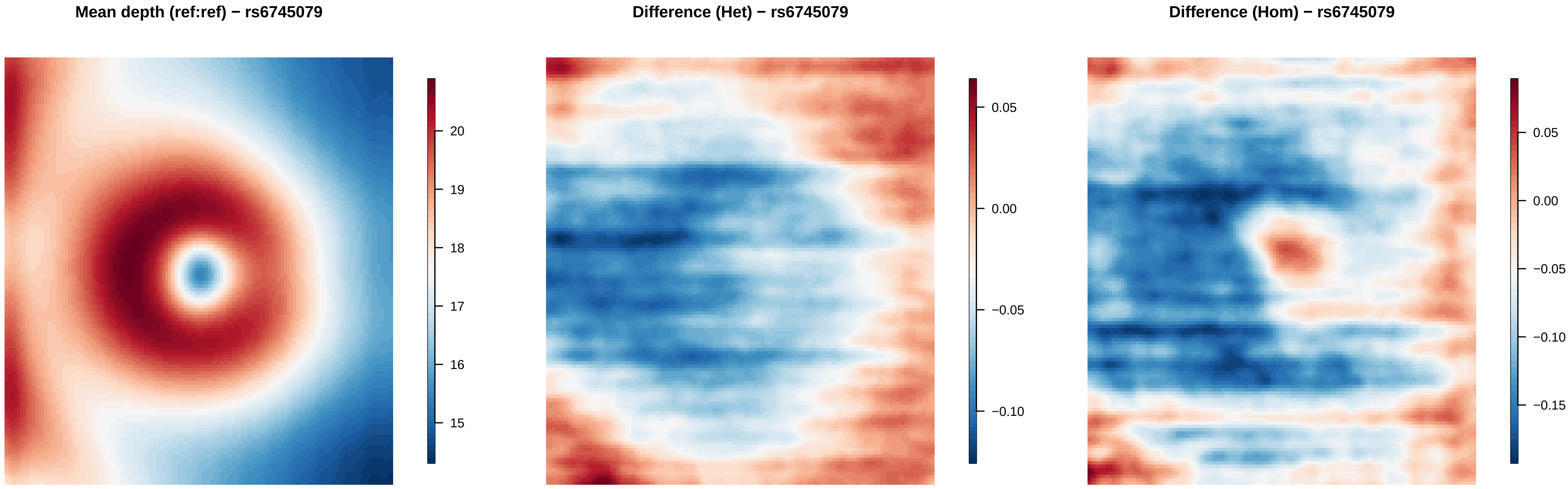

Mean depth (ref:ref) − rs6745079
20
19
18
17
16
15
Difference (Het) − rs6745079
0.05
0.00
−0.05
−0.10
Difference (Hom) − rs6745079
0.05
0.00
−0.05
−0.10
−0.15

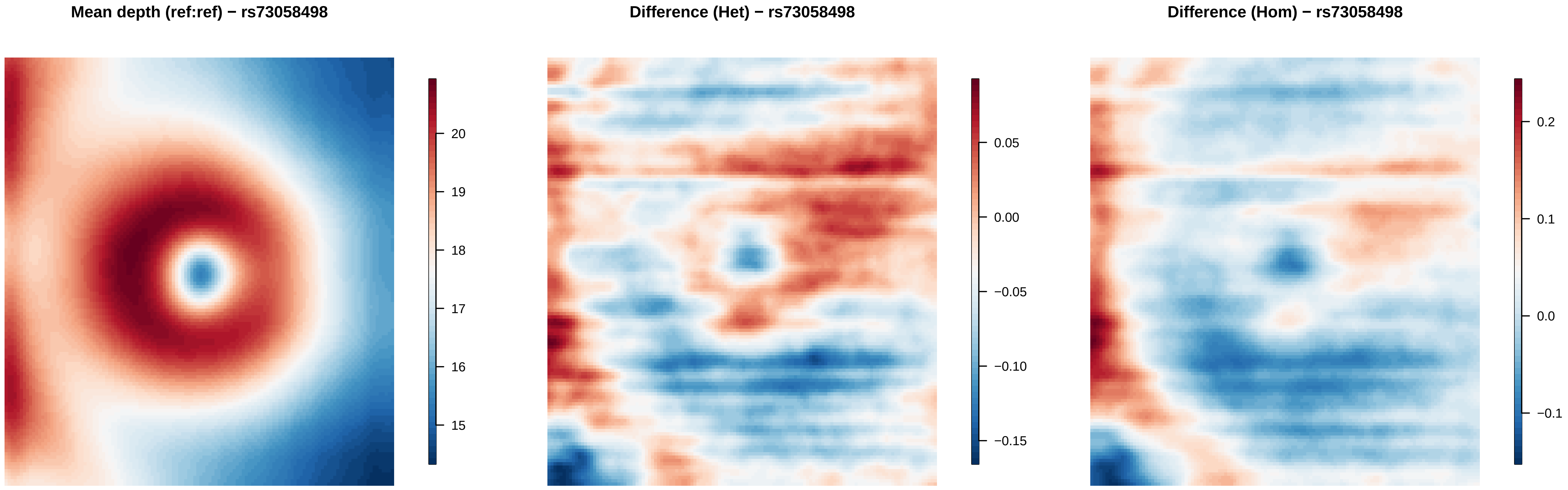

Mean depth (ref:ref) − rs73058498
20
19
18
17
16
15
Difference (Het) − rs73058498
0.05
0.00
−0.05
−0.10
−0.15
Difference (Hom) − rs73058498
0.2
0.1
0.0
−0.1

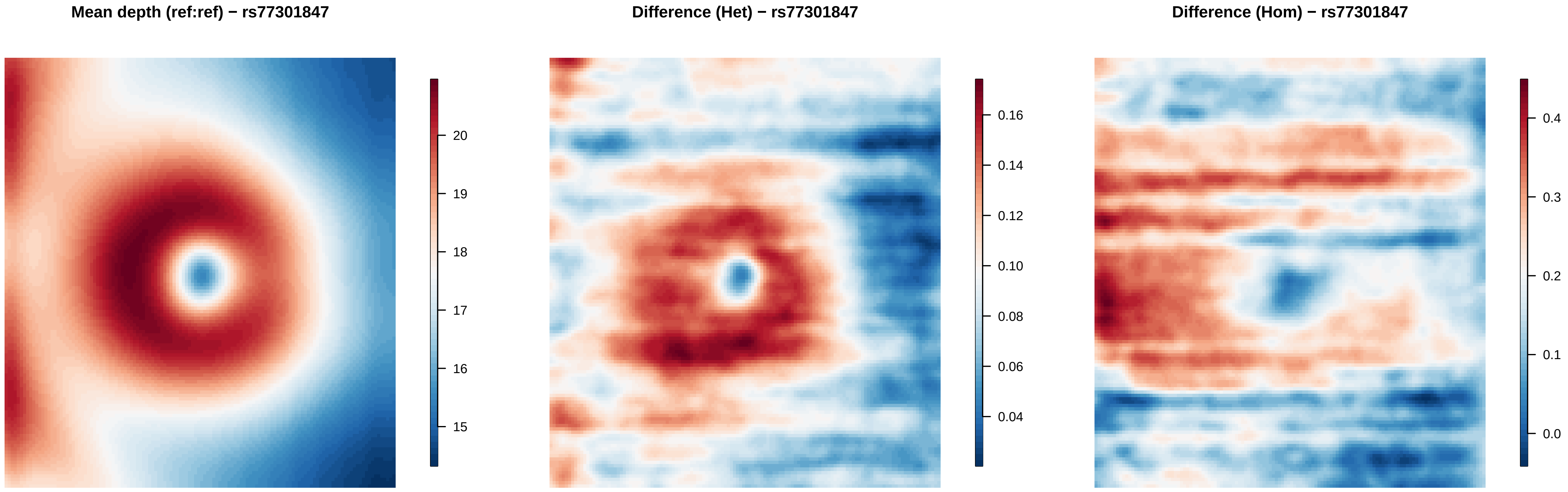
Mean depth (ref:ref) − rs77301847
20
19
18
17
16
15
Difference (Het) − rs77301847
0.16
0.14
0.12
0.10
0.08
0.06
0.04
Difference (Hom) − rs77301847
0.4
0.3
0.2
0.1
0.0

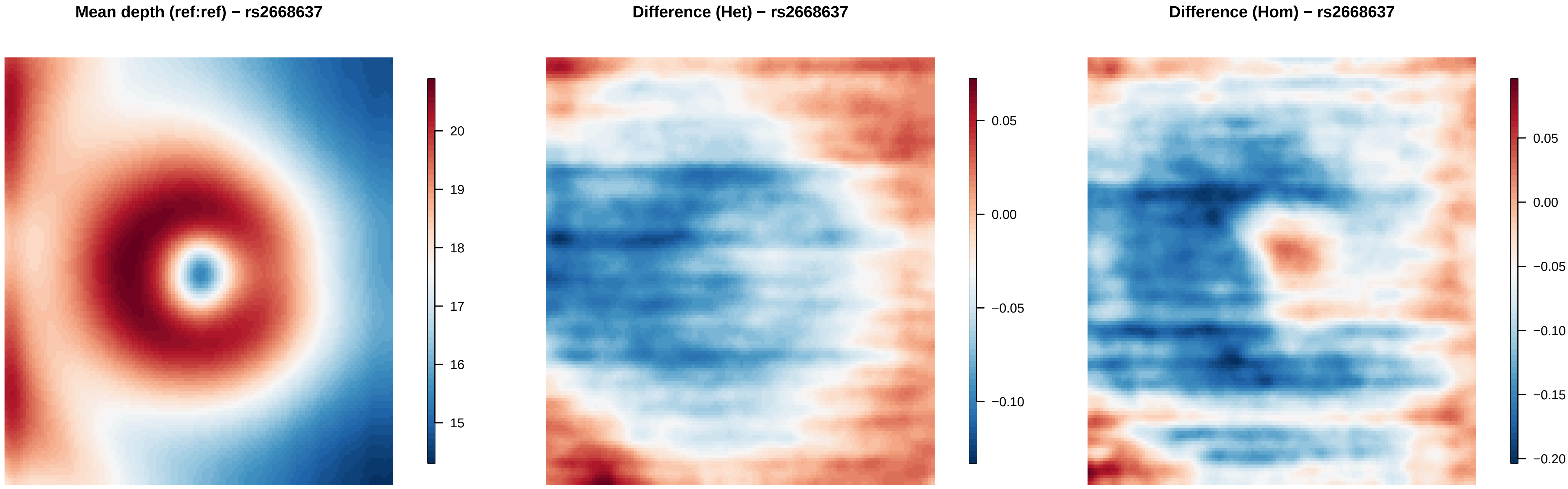

Mean depth (ref:ref) − rs2668637
20
19
18
17
16
15
Difference (Het) − rs2668637
0.05
0.00
−0.05
−0.10
Difference (Hom) − rs2668637
0.05
0.00
−0.05
−0.10
−0.15
−0.20

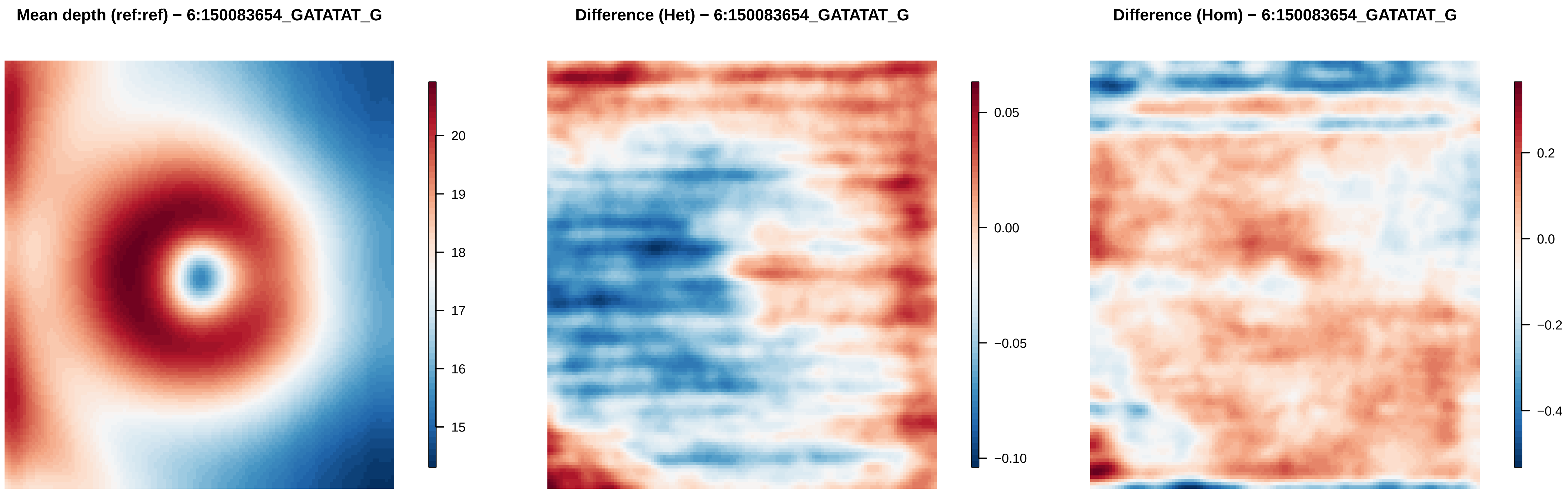

Mean depth (ref:ref) − 6:150083654_GATATAT_G
20
19
18
17
16
15
Difference (Het) − 6:150083654_GATATAT_G
0.05
0.00
−0.05
−0.10
Difference (Hom) − 6:150083654_GATATAT_G
0.2
0.0
−0.2
−0.4

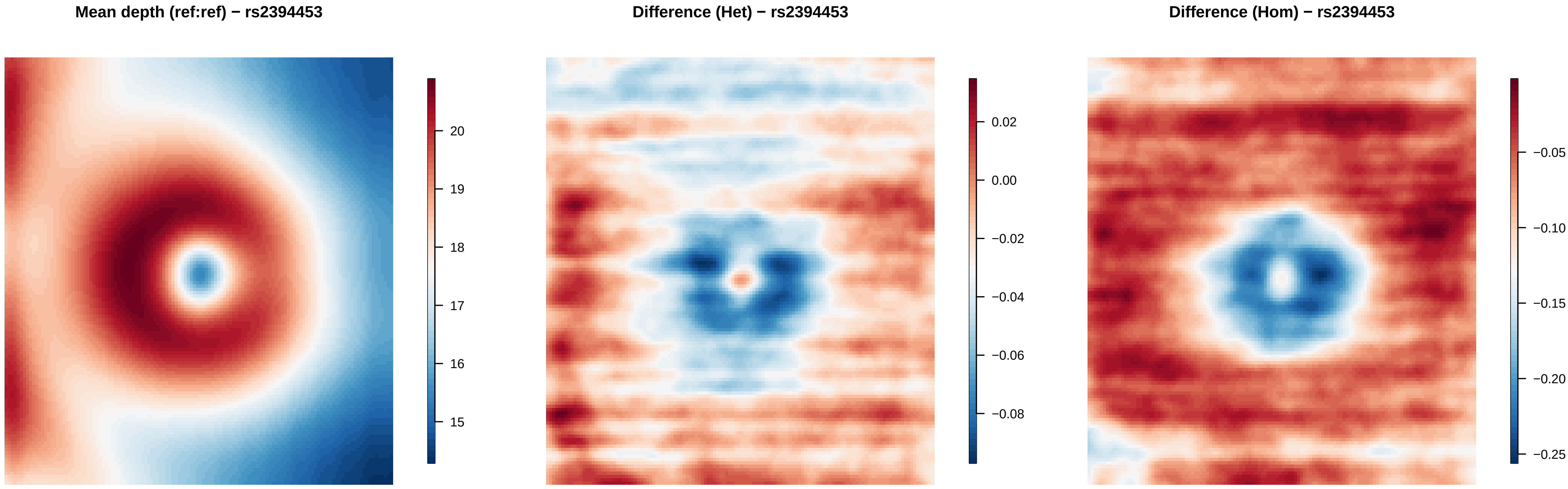
Mean depth (ref:ref) − rs2394453
20
19
18
17
16
15
Difference (Het) − rs2394453
0.02
0.00
−0.02
−0.04
−0.06
−0.08
Difference (Hom) − rs2394453
−0.05
−0.10
−0.15
−0.20
−0.25

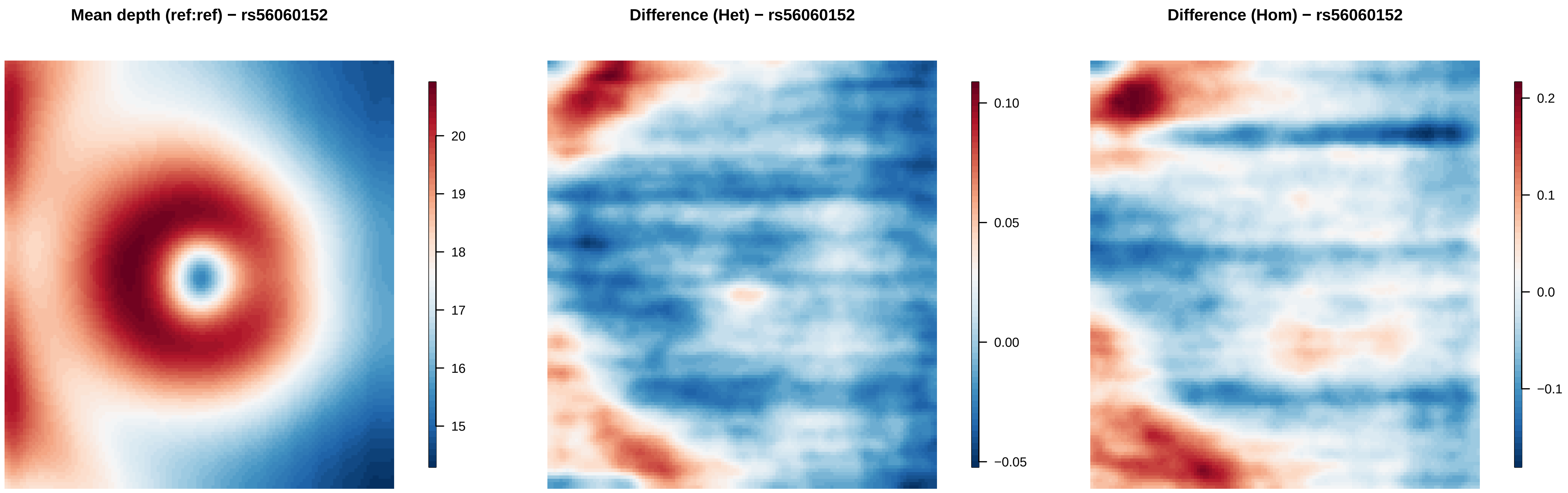
Mean depth (ref:ref) − rs56060152
20
19
18
17
16
15
Difference (Het) − rs56060152
0.10
0.05
0.00
−0.05
Difference (Hom) − rs56060152
0.2
0.1
0.0
−0.1

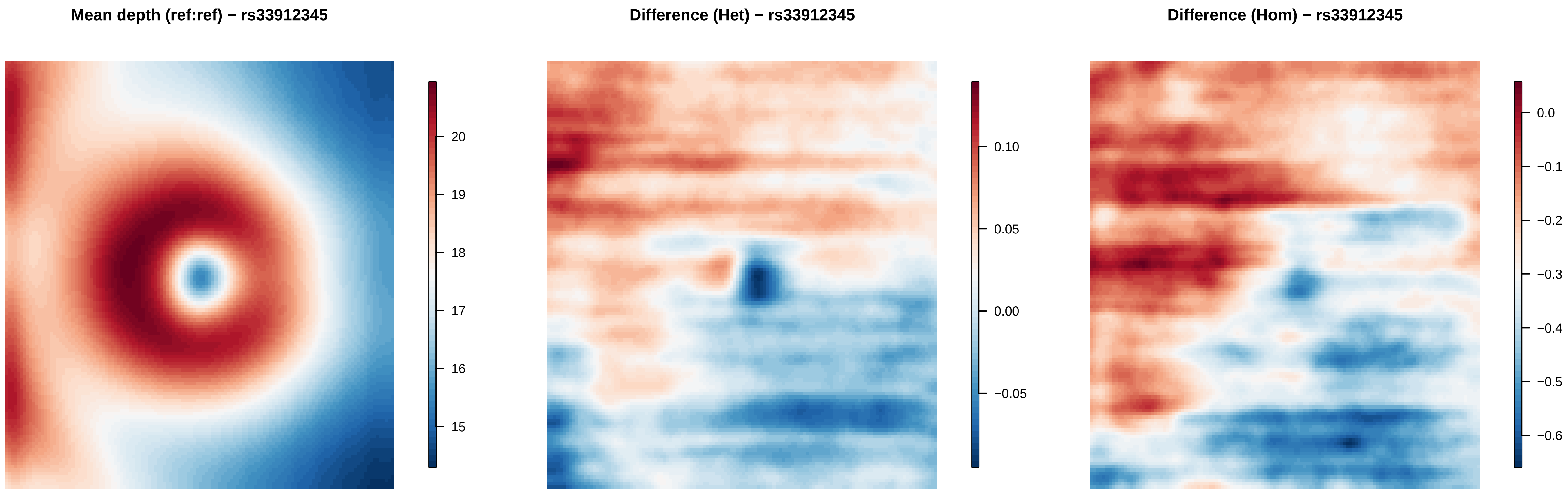
Mean depth (ref:ref) − rs33912345
20
19
18
17
16
15
Difference (Het) − rs33912345
0.10
0.05
0.00
−0.05
Difference (Hom) − rs33912345
0.0
−0.1
−0.2
−0.3
−0.4
−0.5
−0.6

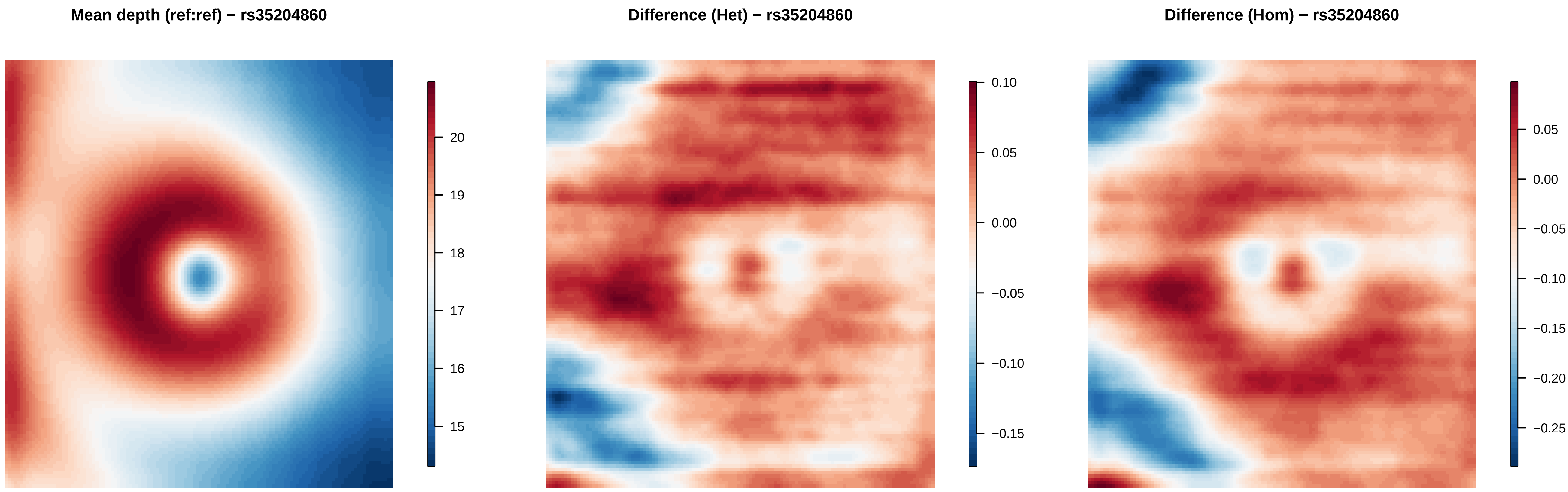
Mean depth (ref:ref) − rs35204860
20
19
18
17
16
15
Difference (Het) − rs35204860
0.10
0.05
0.00
−0.05
−0.10
−0.15
Difference (Hom) − rs35204860
0.05
0.00
−0.05
−0.10
−0.15
−0.20
−0.25

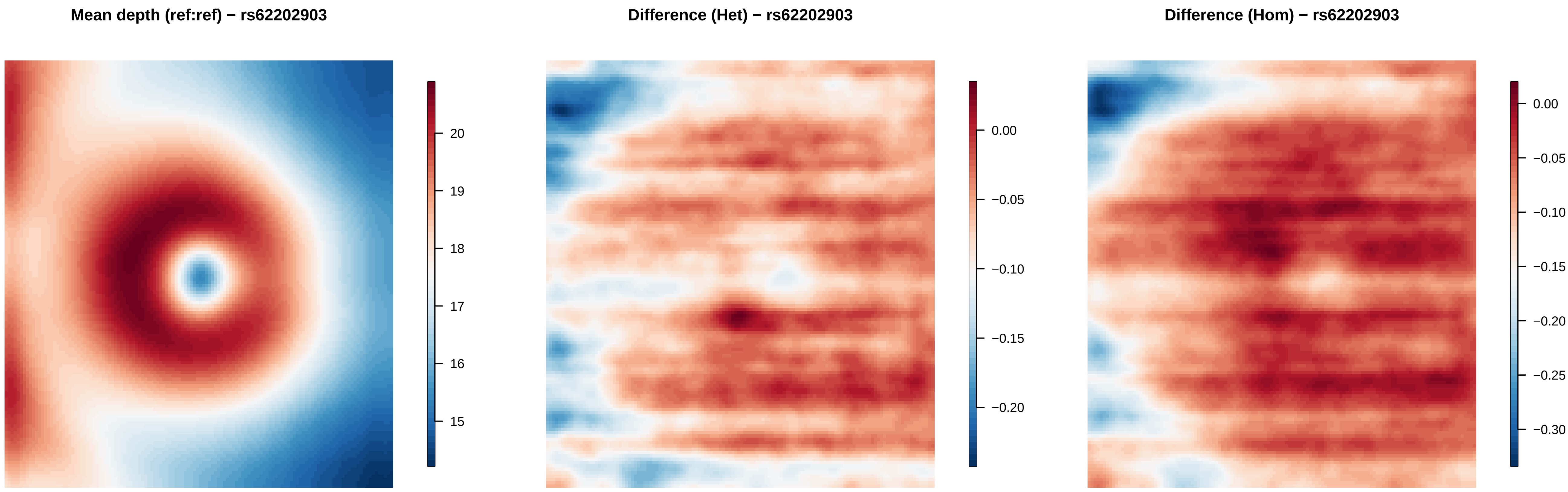
Mean depth (ref:ref) − rs62202903
20
19
18
17
16
15
Difference (Het) − rs62202903
0.00
−0.05
−0.10
−0.15
−0.20
Difference (Hom) − rs62202903
0.00
−0.05
−0.10
−0.15
−0.20
−0.25
−0.30

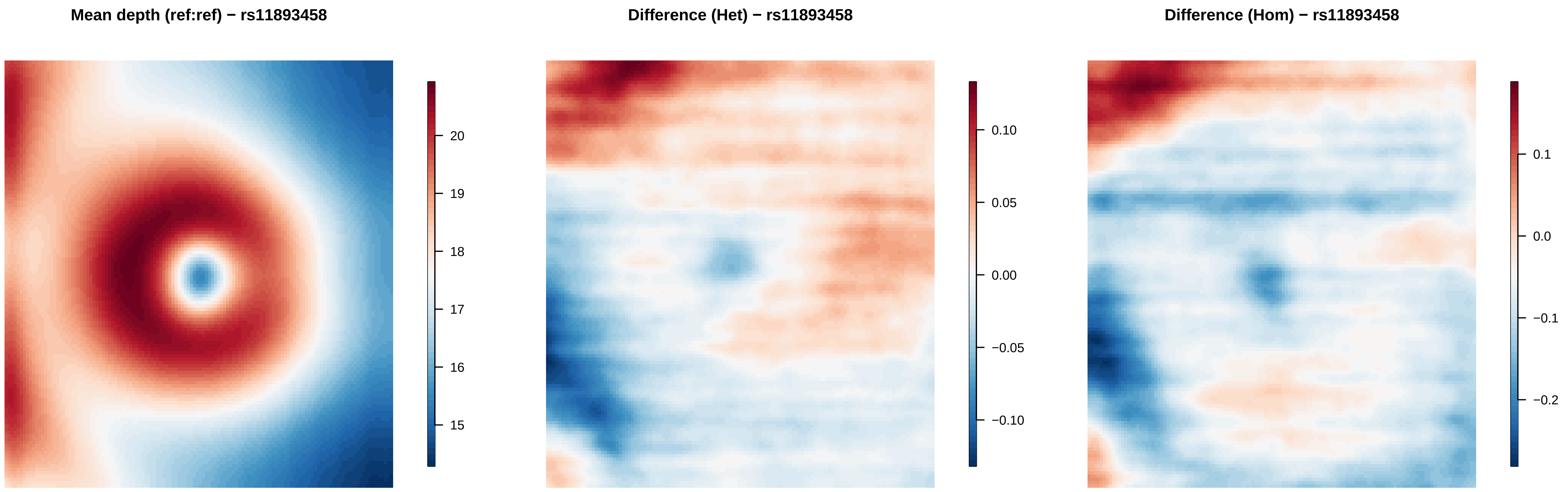
Mean depth (ref:ref) − rs11893458
20
19
18
17
16
15
Difference (Het) − rs11893458
0.10
0.05
0.00
−0.05
−0.10
Difference (Hom) − rs11893458
0.1
0.0
−0.1
−0.2

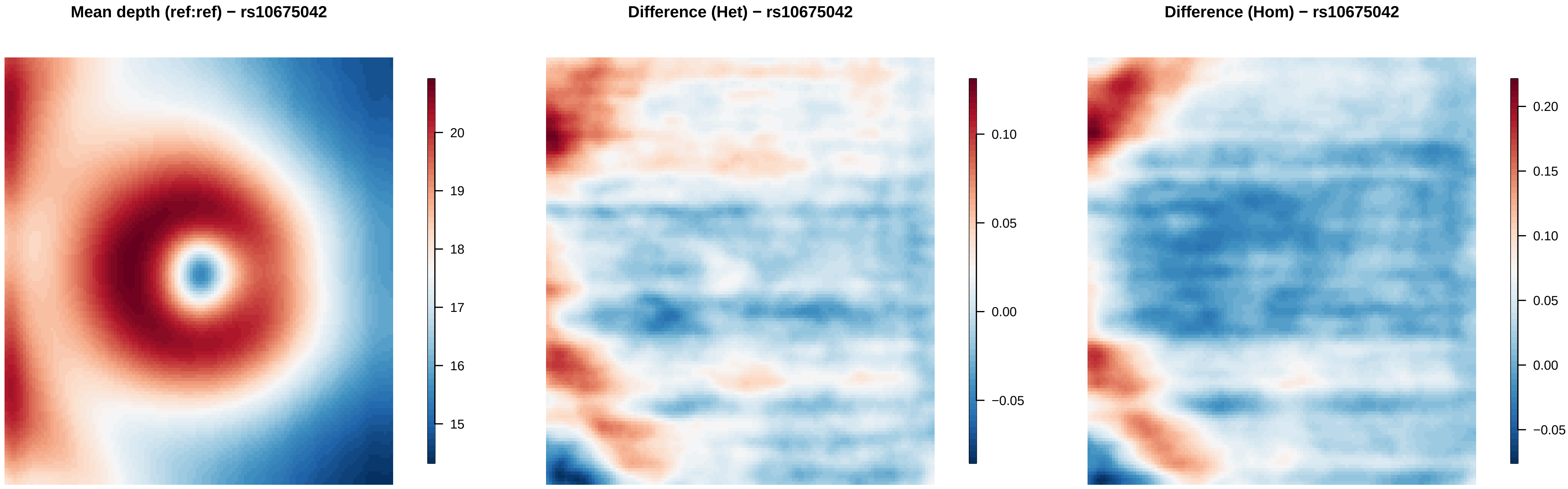

Mean depth (ref:ref) − rs10675042
20
19
18
17
16
15
Difference (Het) − rs10675042
0.10
0.05
0.00
−0.05
Difference (Hom) − rs10675042
0.20
0.15
0.10
0.05
0.00
−0.05

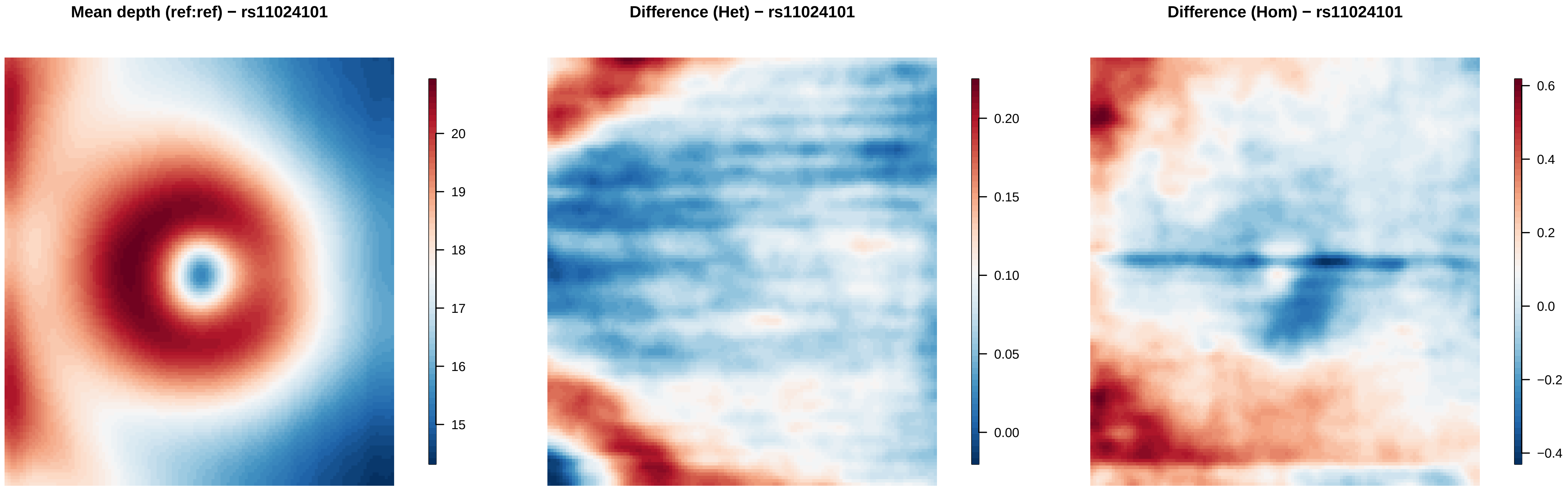
Mean depth (ref:ref) − rs11024101
20
19
18
17
16
15
Difference (Het) − rs11024101
0.20
0.15
0.10
0.05
0.00
Difference (Hom) − rs11024101
0.6
0.4
0.2
0.0
−0.2
−0.4

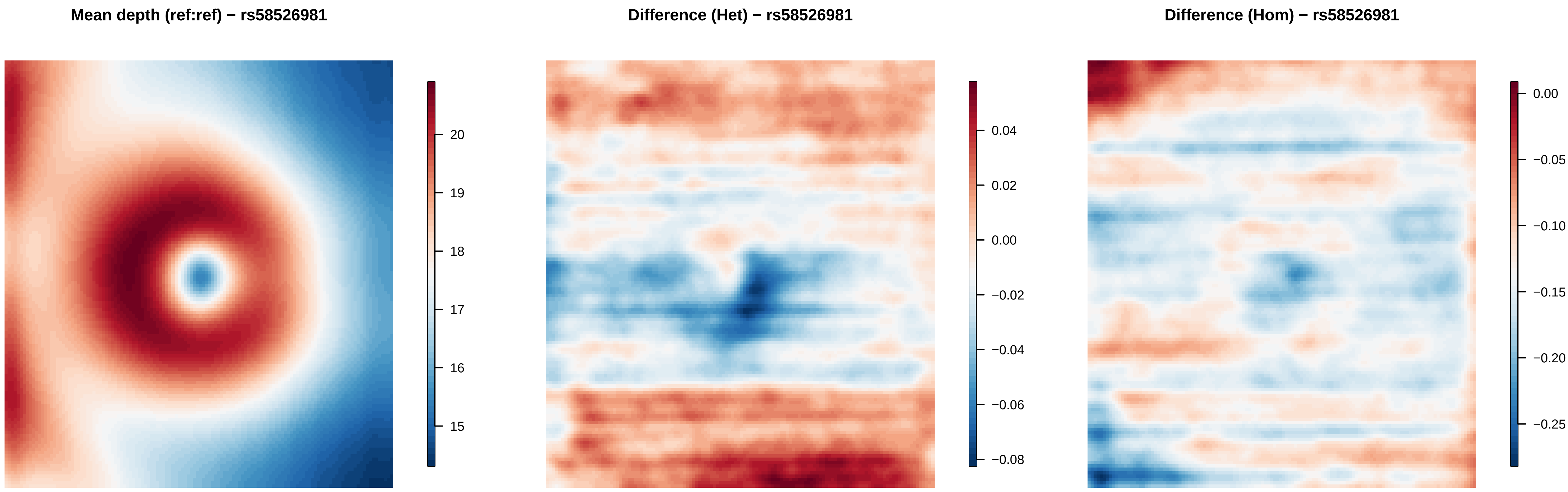
Mean depth (ref:ref) − rs58526981
20
19
18
17
16
15
Difference (Het) − rs58526981
0.04
0.02
0.00
−0.02
−0.04
−0.06
−0.08
Difference (Hom) − rs58526981
0.00
−0.05
−0.10
−0.15
−0.20
−0.25

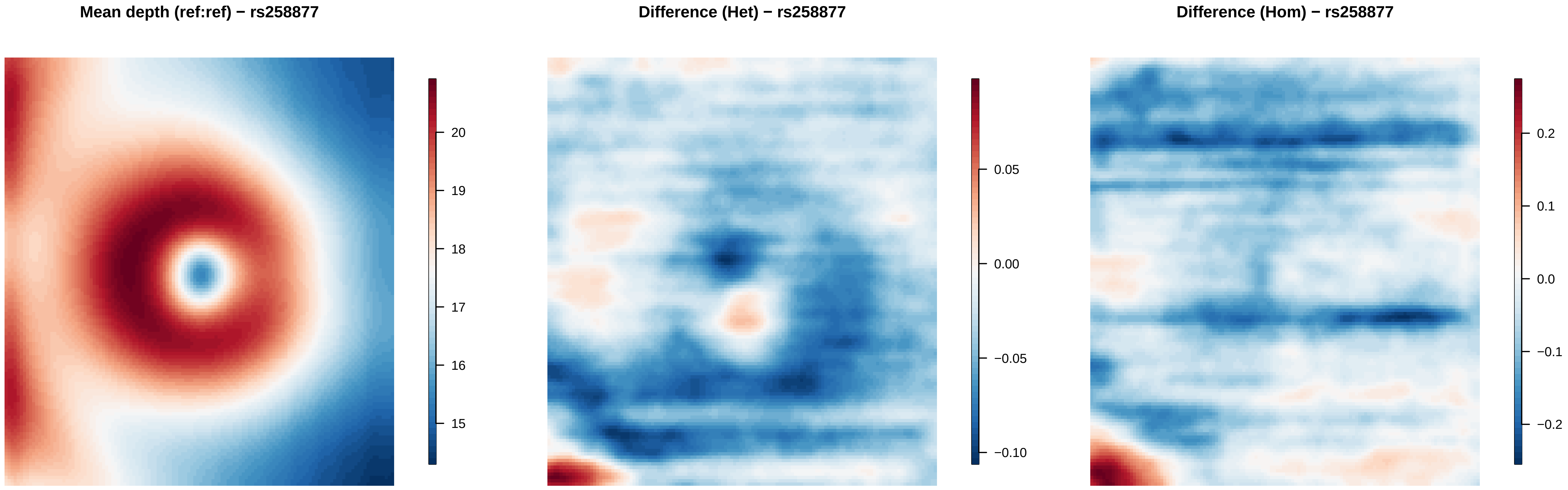
Mean depth (ref:ref) − rs258877
20
19
18
17
16
15
Difference (Het) − rs258877
0.05
0.00
−0.05
−0.10
Difference (Hom) − rs258877
0.2
0.1
0.0
−0.1
−0.2

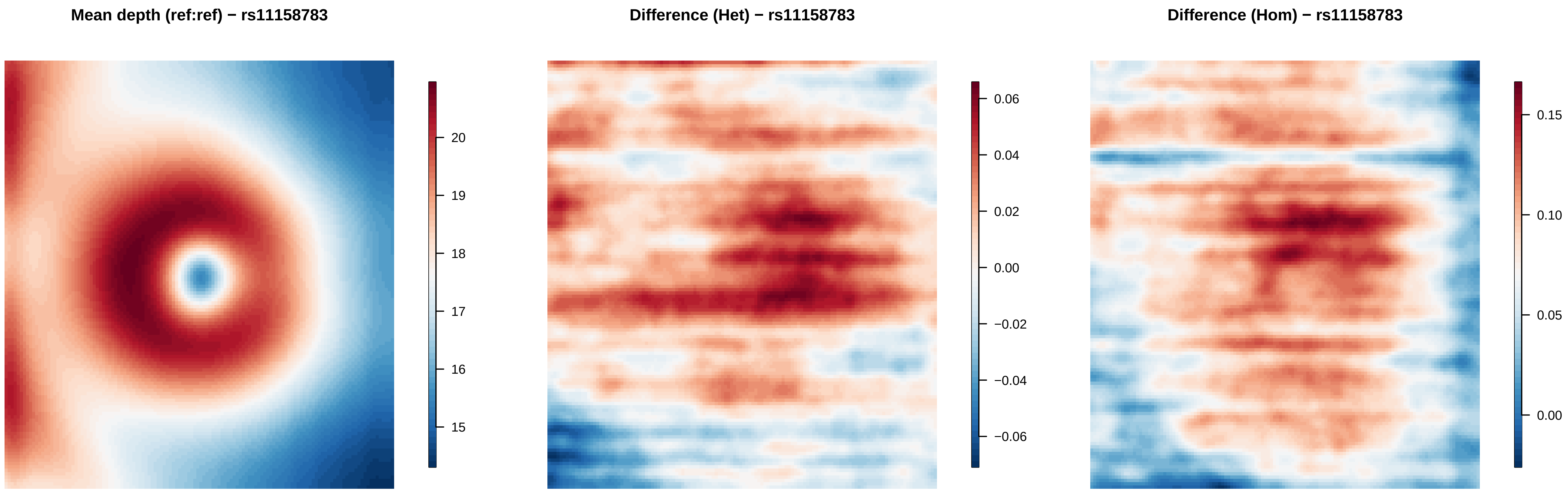
Mean depth (ref:ref) − rs11158783
20
19
18
17
16
15
Difference (Het) − rs11158783
0.06
0.04
0.02
0.00
−0.02
−0.04
−0.06
Difference (Hom) − rs11158783
0.15
0.10
0.05
0.00

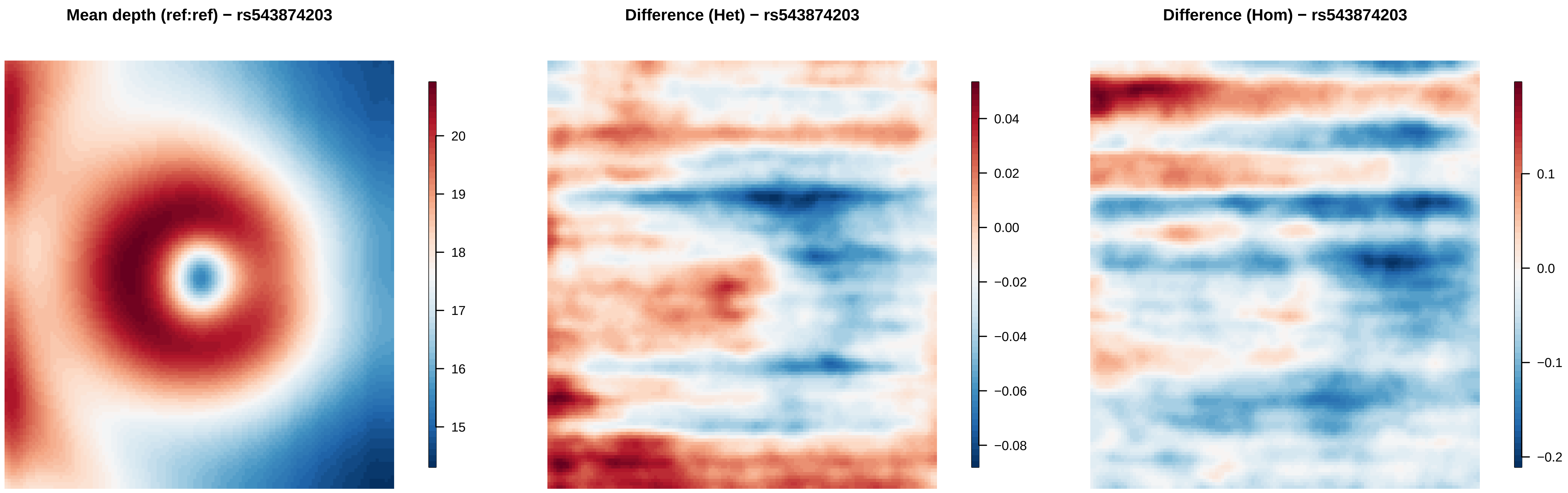

Mean depth (ref:ref) − rs543874203
20
19
18
17
16
15
Difference (Het) − rs543874203
0.04
0.02
0.00
−0.02
−0.04
−0.06
−0.08
Difference (Hom) − rs543874203
0.1
0.0
−0.1
−0.2

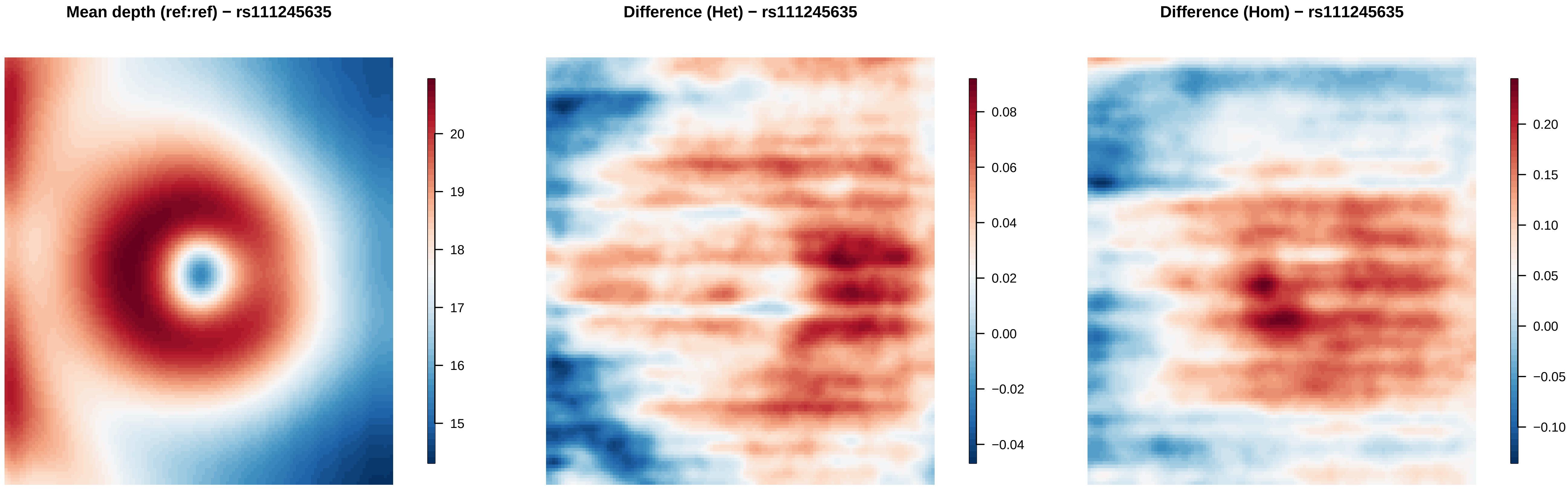
Mean depth (ref:ref) − rs111245635
20
19
18
17
16
15
Difference (Het) − rs111245635
0.08
0.06
0.04
0.02
0.00
−0.02
−0.04
Difference (Hom) − rs111245635
0.20
0.15
0.10
0.05
0.00
−0.05
−0.10

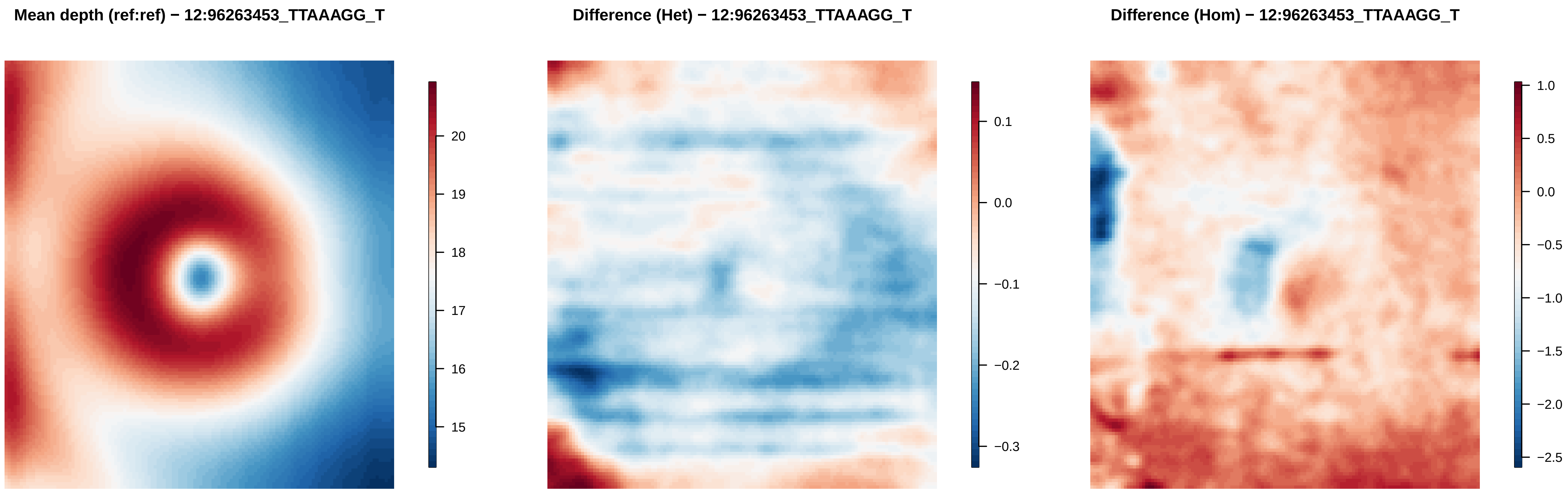
Mean depth (ref:ref) − 12:96263453_TTAAAGG_T
20
19
18
17
16
15
Difference (Het) − 12:96263453_TTAAAGG_T
0.1
0.0
−0.1
−0.2
−0.3
Difference (Hom) − 12:96263453_TTAAAGG_T
1.0
0.5
0.0
−0.5
−1.0
−1.5
−2.0
−2.5

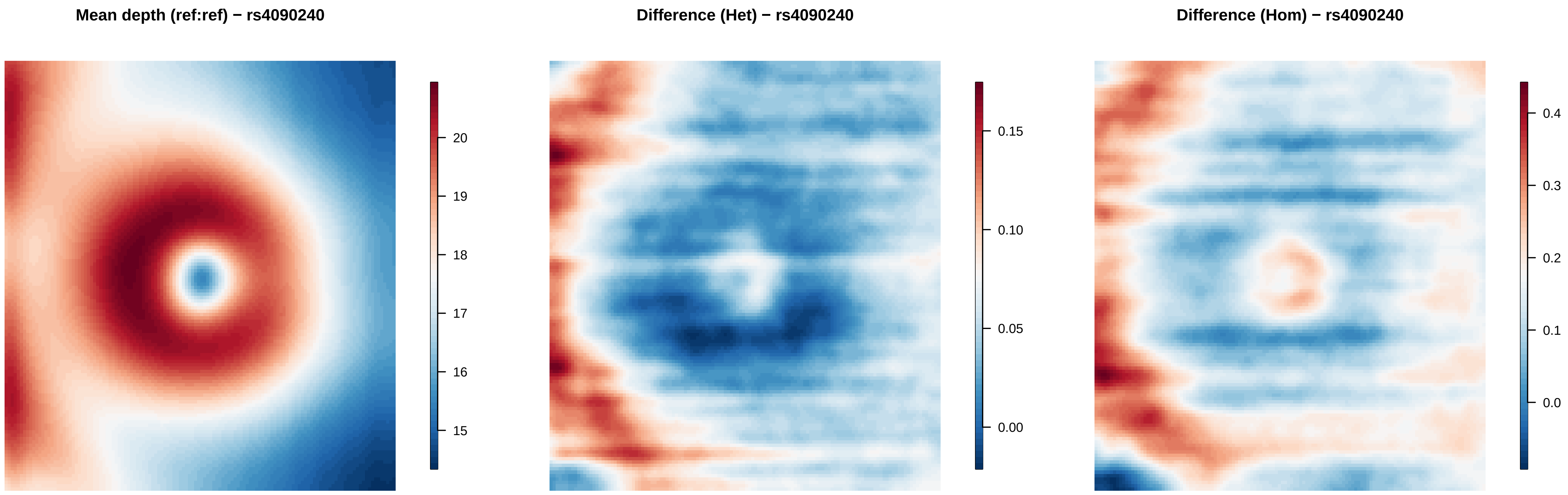
Mean depth (ref:ref) − rs4090240
20
19
18
17
16
15
Difference (Het) − rs4090240
0.15
0.10
0.05
0.00
Difference (Hom) − rs4090240
0.4
0.3
0.2
0.1
0.0

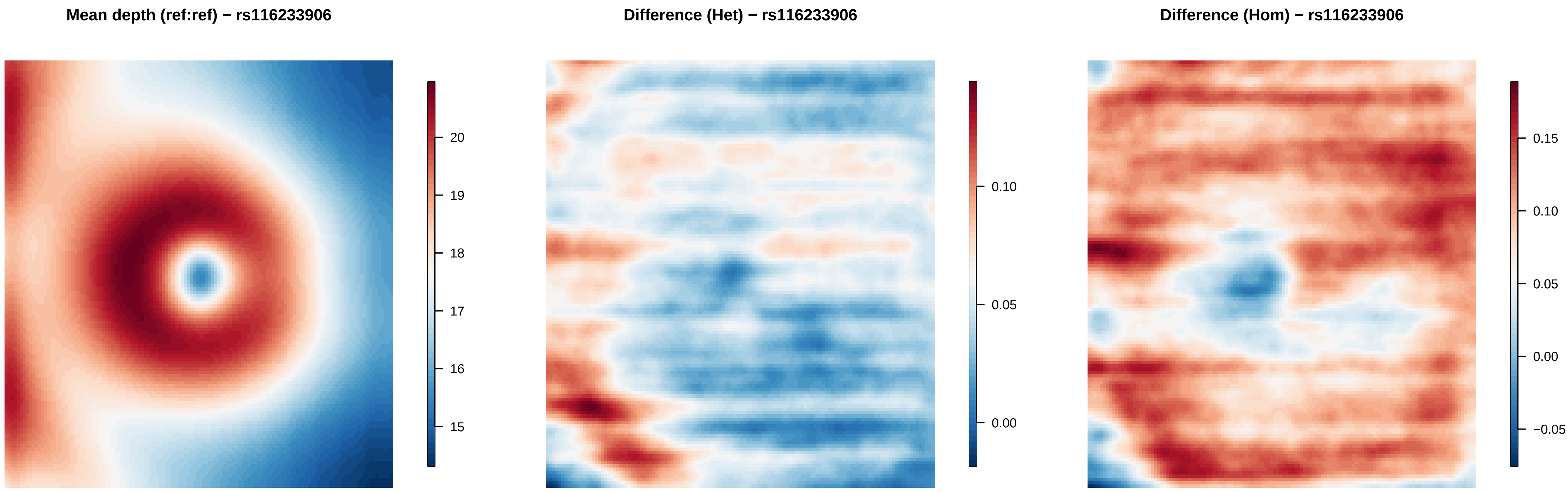
Mean depth (ref:ref) − rs116233906
20
19
18
17
16
15
Difference (Het) − rs116233906
0.10
0.05
0.00
Difference (Hom) − rs116233906
0.15
0.10
0.05
0.00
−0.05

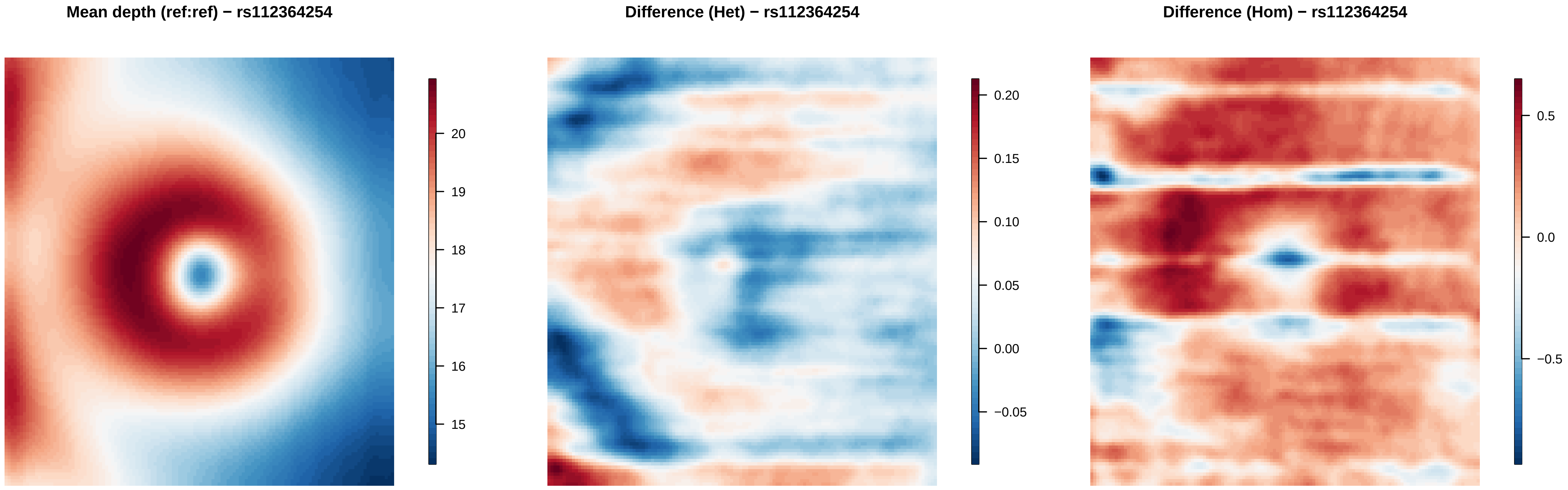
Mean depth (ref:ref) − rs112364254
20
19
18
17
16
15
Difference (Het) − rs112364254
0.20
0.15
0.10
0.05
0.00
−0.05
Difference (Hom) − rs112364254
0.5
0.0
−0.5

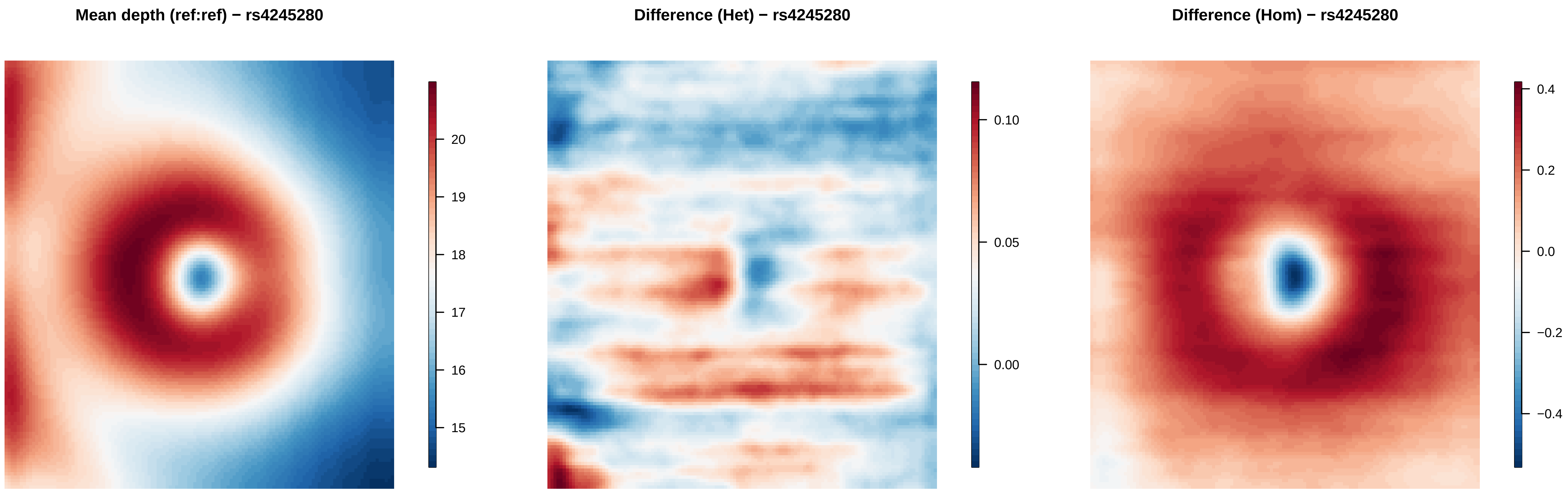

Mean depth (ref:ref) − rs4245280
20
19
18
17
16
15
Difference (Het) − rs4245280
0.10
0.05
0.00
Difference (Hom) − rs4245280
0.4
0.2
0.0
−0.2
−0.4

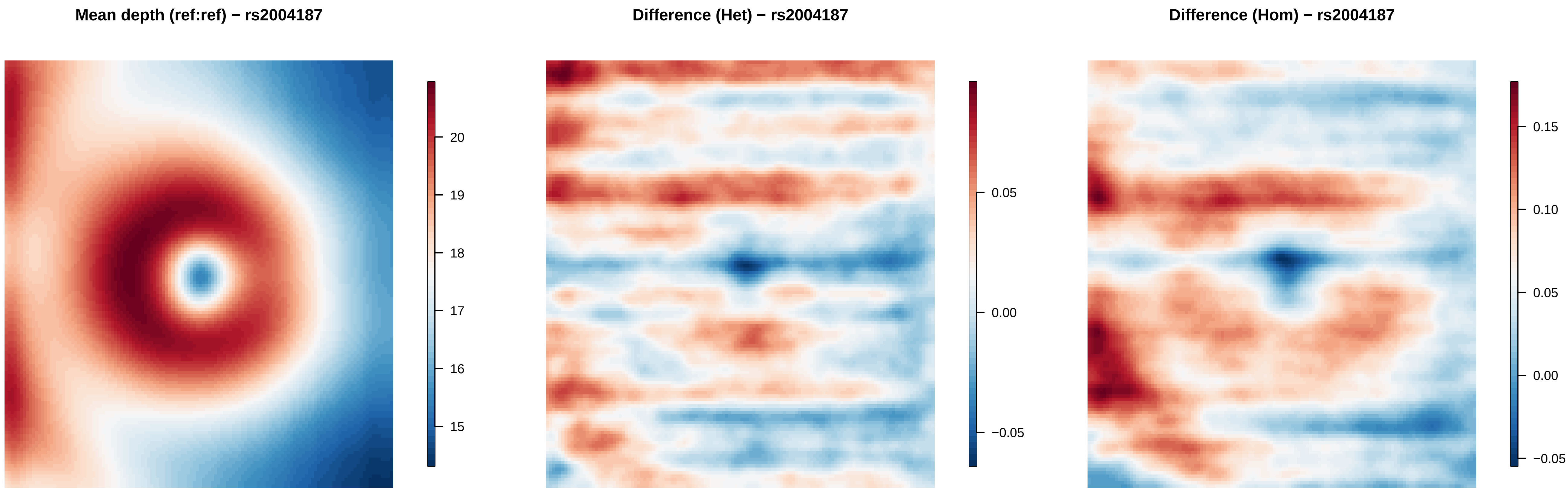

Mean depth (ref:ref) − rs2004187
20
19
18
17
16
15
Difference (Het) − rs2004187
0.05
0.00
−0.05
Difference (Hom) − rs2004187
0.15
0.10
0.05
0.00
−0.05

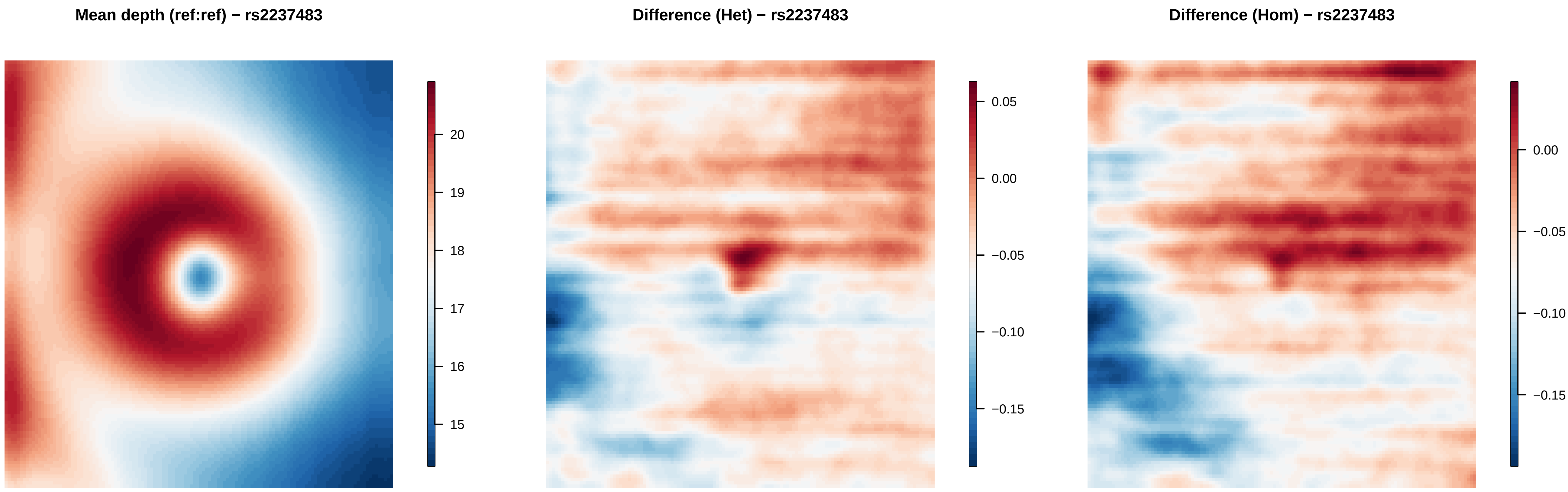
Mean depth (ref:ref) − rs2237483
20
19
18
17
16
15
Difference (Het) − rs2237483
0.05
0.00
−0.05
−0.10
−0.15
Difference (Hom) − rs2237483
0.00
−0.05
−0.10
−0.15

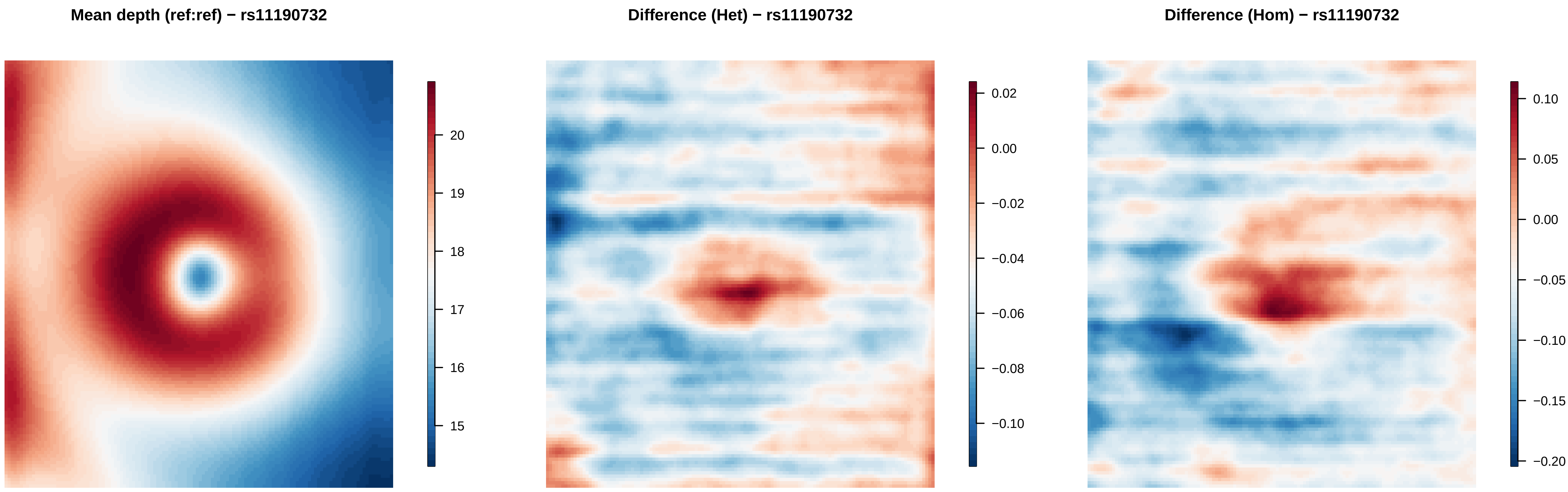
Mean depth (ref:ref) − rs11190732
20
19
18
17
16
15
Difference (Het) − rs11190732
0.02
0.00
−0.02
−0.04
−0.06
−0.08
−0.10
Difference (Hom) − rs11190732
0.10
0.05
0.00
−0.05
−0.10
−0.15
−0.20

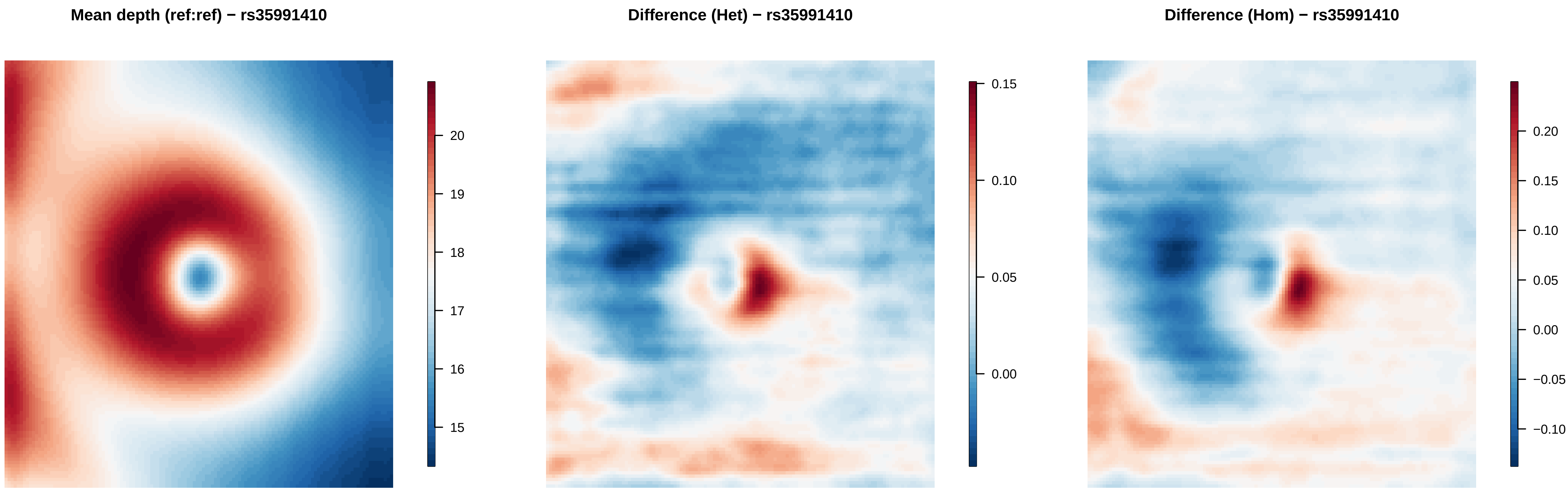
Mean depth (ref:ref) − rs35991410
20
19
18
17
16
15
Difference (Het) − rs35991410
0.15
0.10
0.05
0.00
Difference (Hom) − rs35991410
0.20
0.15
0.10
0.05
0.00
−0.05
−0.10

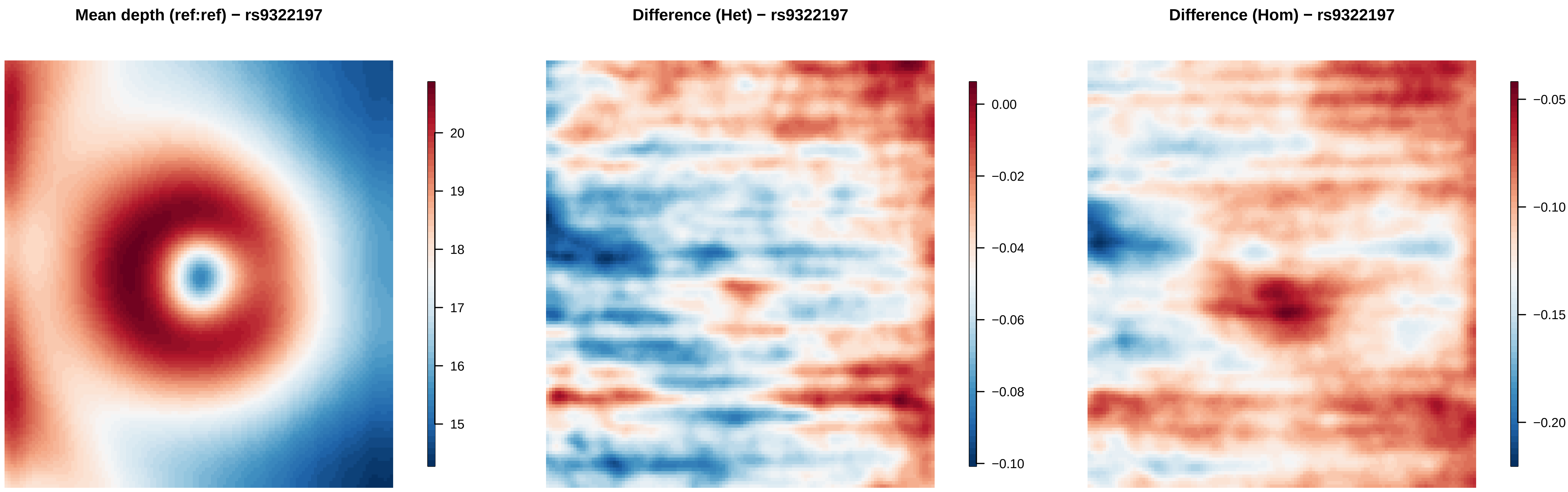

Mean depth (ref:ref) − rs9322197
20
19
18
17
16
15
Difference (Het) − rs9322197
0.00
−0.02
−0.04
−0.06
−0.08
−0.10
Difference (Hom) − rs9322197
−0.05
−0.10
−0.15
−0.20

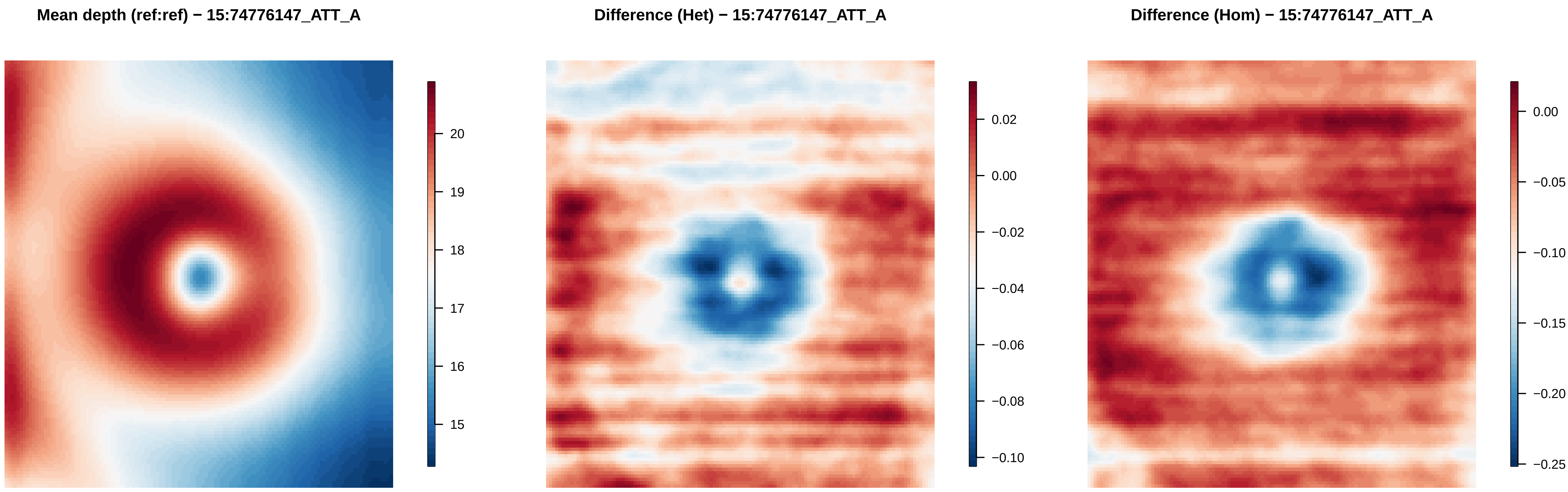
Mean depth (ref:ref) − 15:74776147_ATT_A
20
19
18
17
16
15
Difference (Het) − 15:74776147_ATT_A
0.02
0.00
−0.02
−0.04
−0.06
−0.08
−0.10
Difference (Hom) − 15:74776147_ATT_A
0.00
−0.05
−0.10
−0.15
−0.20
−0.25

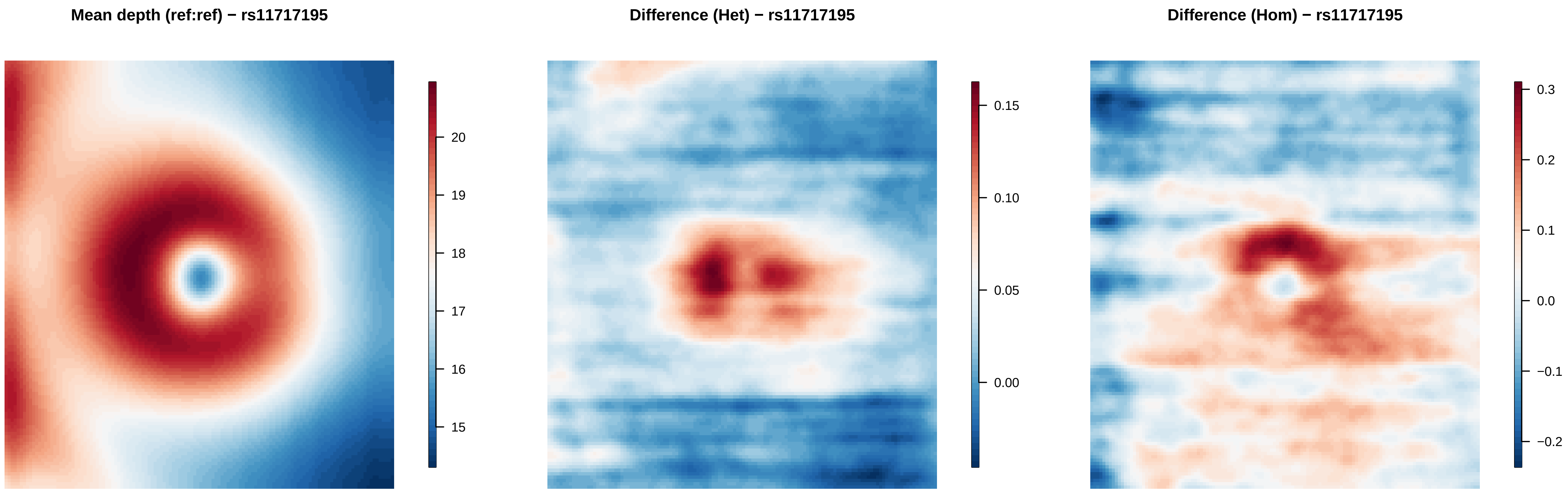

Mean depth (ref:ref) − rs11717195
Difference (Het) − rs11717195
Difference (Hom) − rs11717195
20
19
18
17
16
15
0.15
0.10
0.05
0.00
0.3
0.2
0.1
0.0
−0.1
−0.2

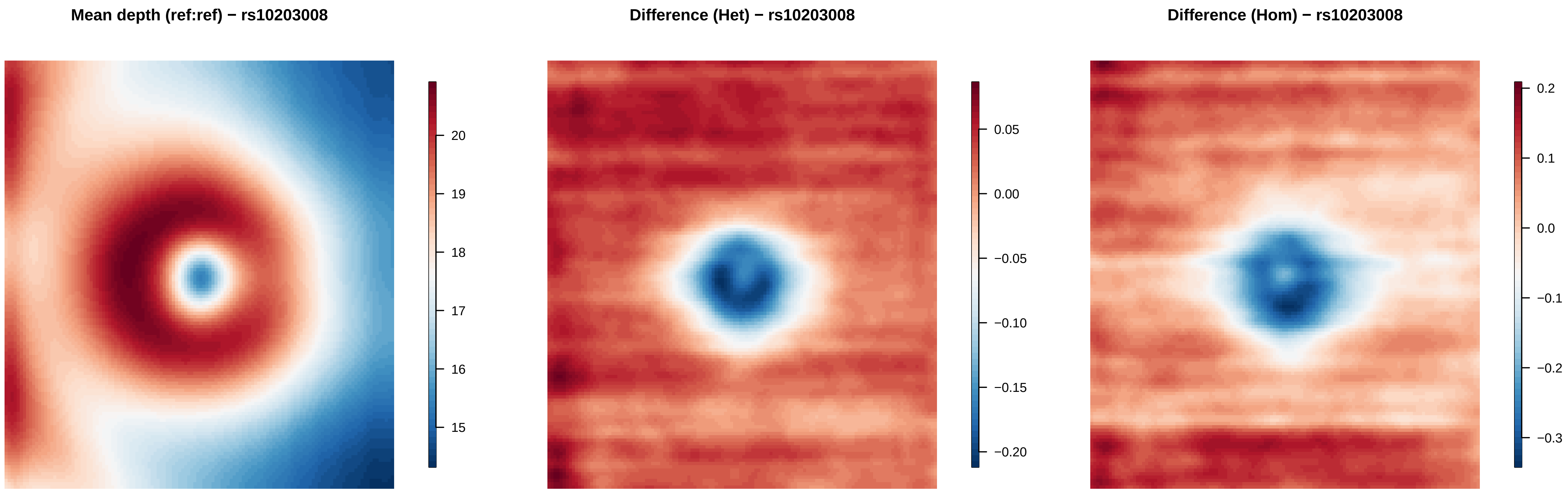
Mean depth (ref:ref) − rs10203008
20
19
18
17
16
15
Difference (Het) − rs10203008
0.05
0.00
−0.05
−0.10
−0.15
−0.20
Difference (Hom) − rs10203008
0.2
0.1
0.0
−0.1
−0.2
−0.3

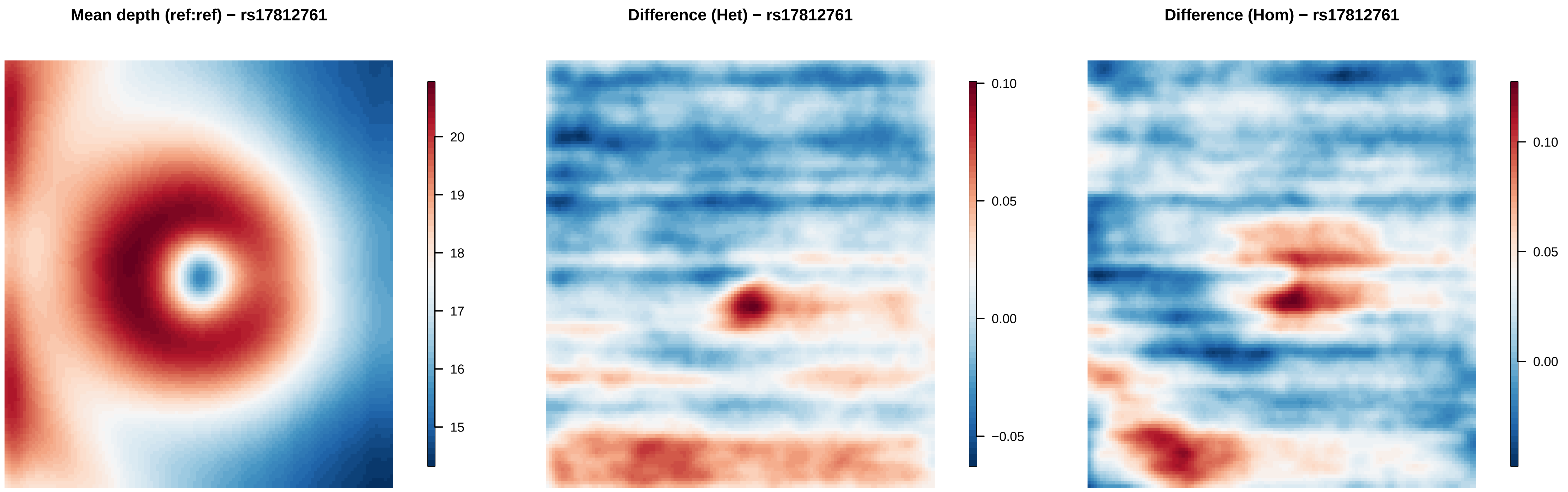

Mean depth (ref:ref) − rs17812761
20
19
18
17
16
15
Difference (Het) − rs17812761
0.10
0.05
0.00
−0.05
Difference (Hom) − rs17812761
0.10
0.05
0.00

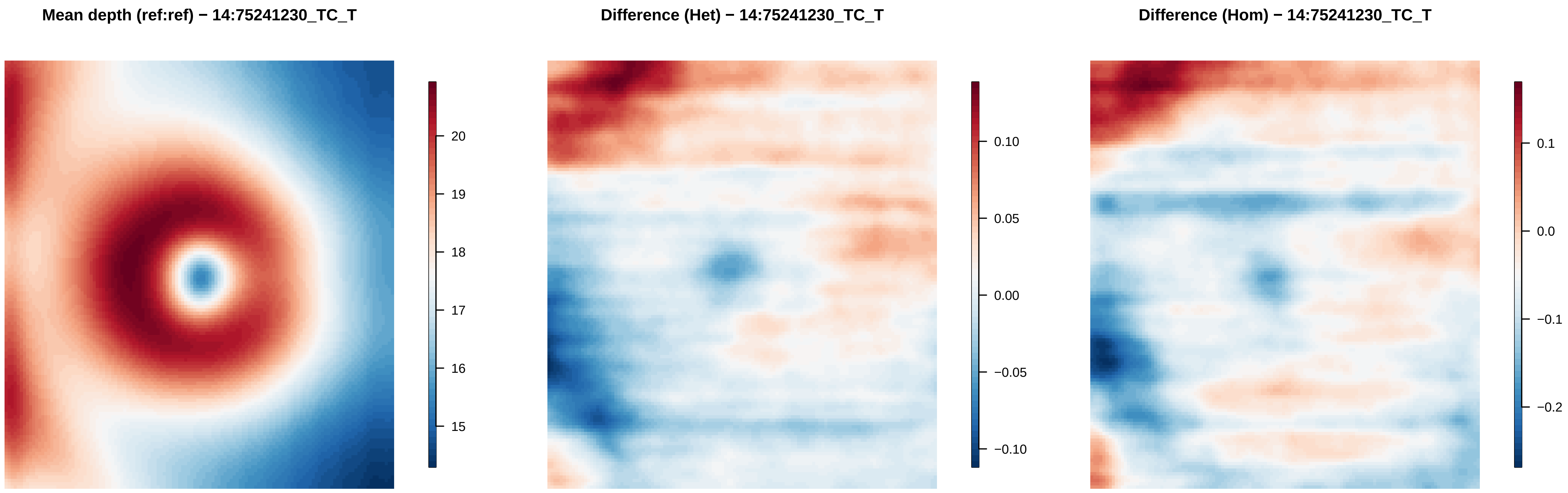
Mean depth (ref:ref) − 14:75241230_TC_T
20
19
18
17
16
15
Difference (Het) − 14:75241230_TC_T
0.10
0.05
0.00
−0.05
−0.10
Difference (Hom) − 14:75241230_TC_T
0.1
0.0
−0.1
−0.2

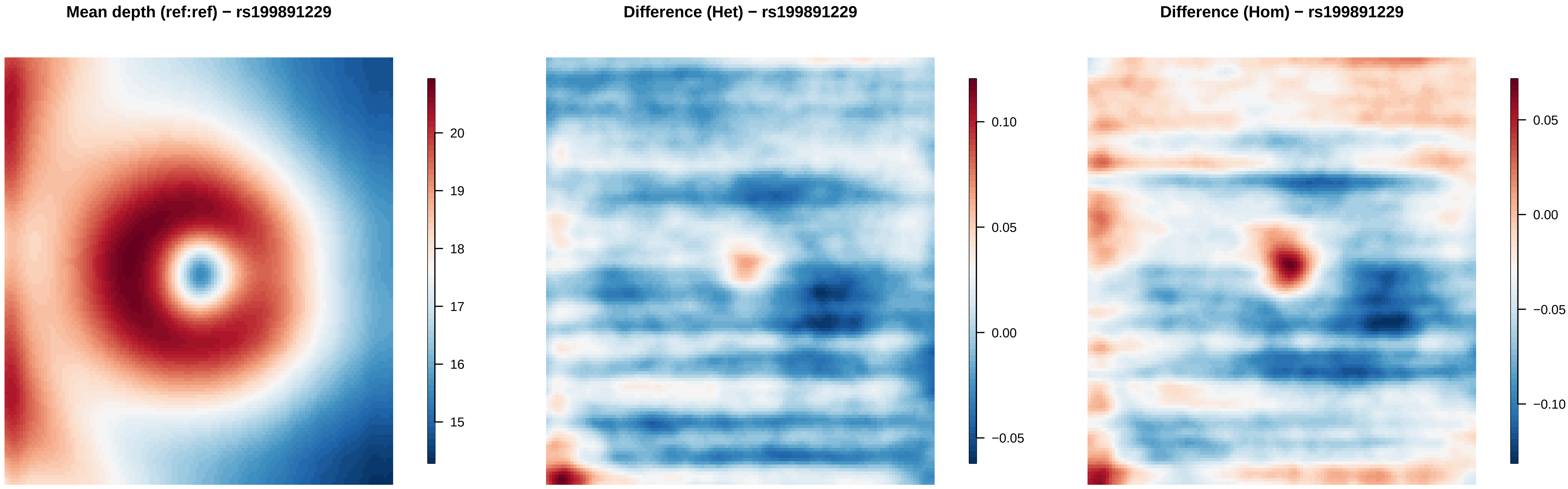

Mean depth (ref:ref) − rs199891229
20
19
18
17
16
15
Difference (Het) − rs199891229
0.10
0.05
0.00
−0.05
Difference (Hom) − rs199891229
0.05
0.00
−0.05
−0.10

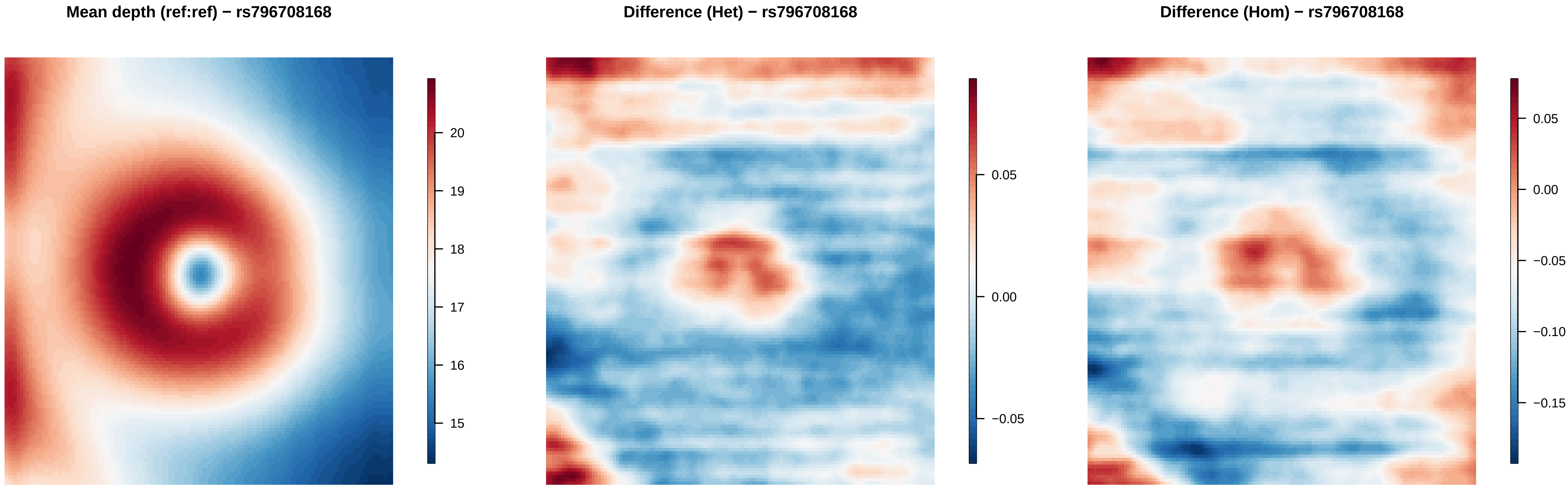
Mean depth (ref:ref) − rs796708168
20
19
18
17
16
15
Difference (Het) − rs796708168
0.05
0.00
−0.05
Difference (Hom) − rs796708168
0.05
0.00
−0.05
−0.10
−0.15

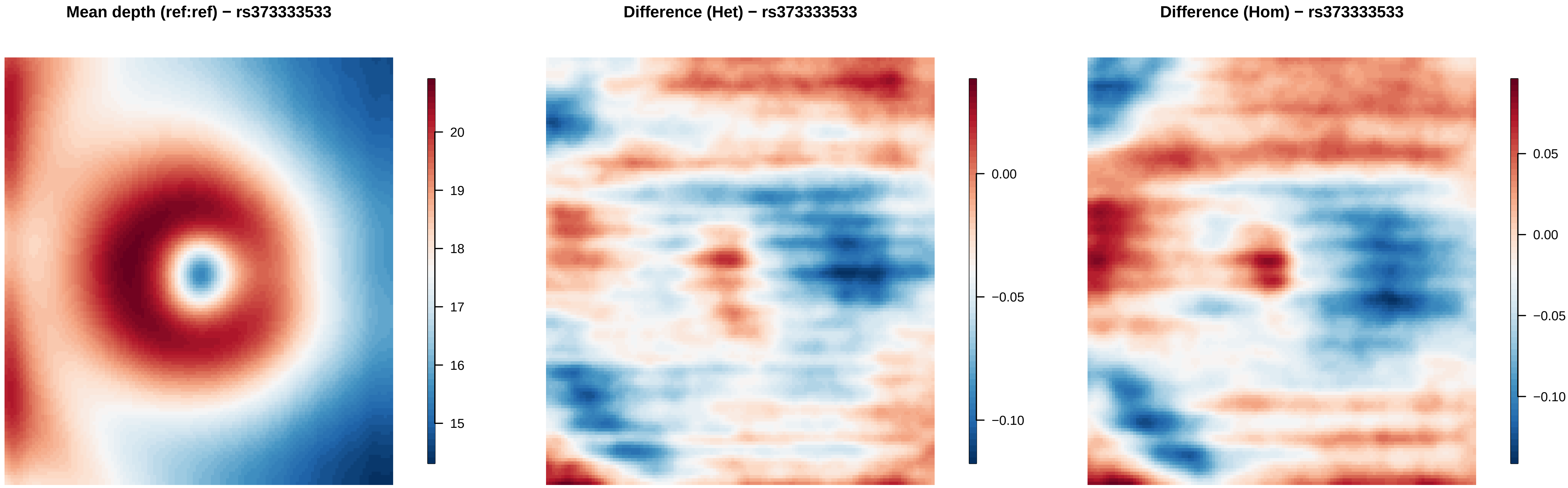
Mean depth (ref:ref) − rs373333533
20
19
18
17
16
15
Difference (Het) − rs373333533
0.00
−0.05
−0.10
Difference (Hom) − rs373333533
0.05
0.00
−0.05
−0.10

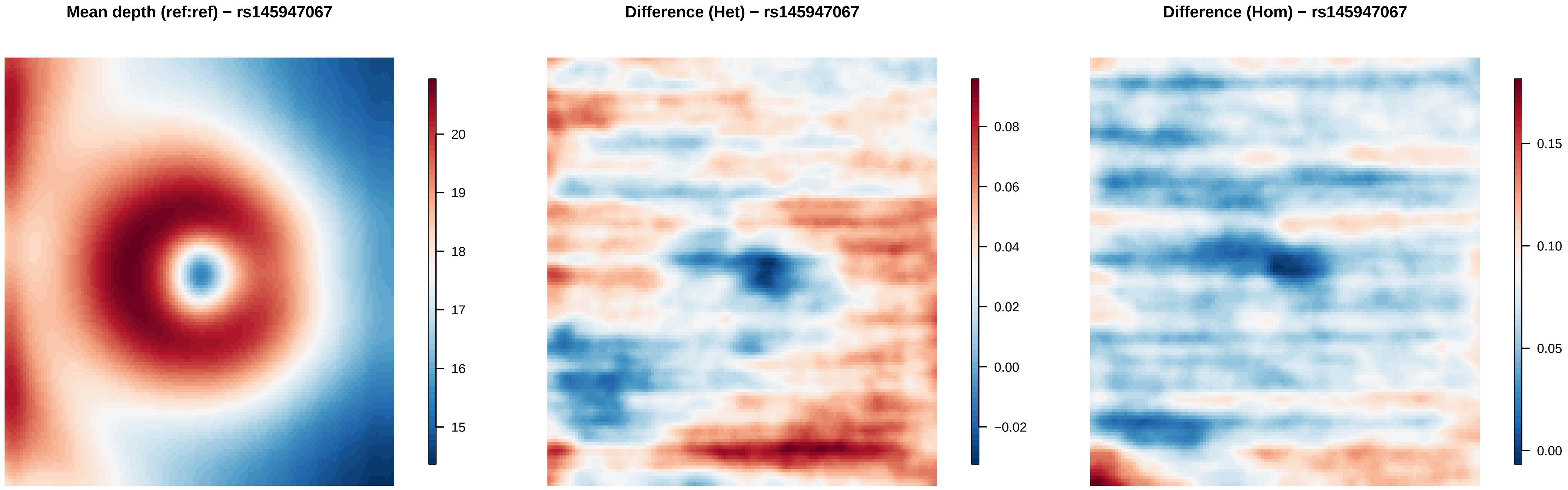

Mean depth (ref:ref) − rs145947067
20
19
18
17
16
15
Difference (Het) − rs145947067
0.08
0.06
0.04
0.02
0.00
−0.02
Difference (Hom) − rs145947067
0.15
0.10
0.05
0.00

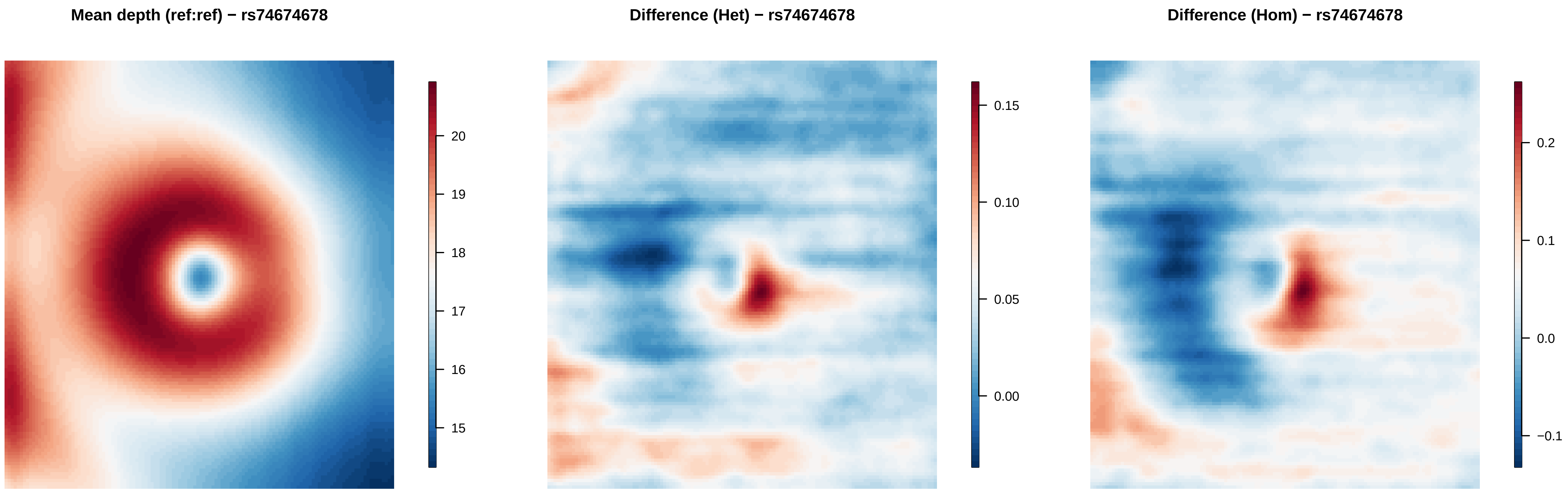

Mean depth (ref:ref) − rs74674678
20
19
18
17
16
15
Difference (Het) − rs74674678
0.15
0.10
0.05
0.00
Difference (Hom) − rs74674678
0.2
0.1
0.0
−0.1

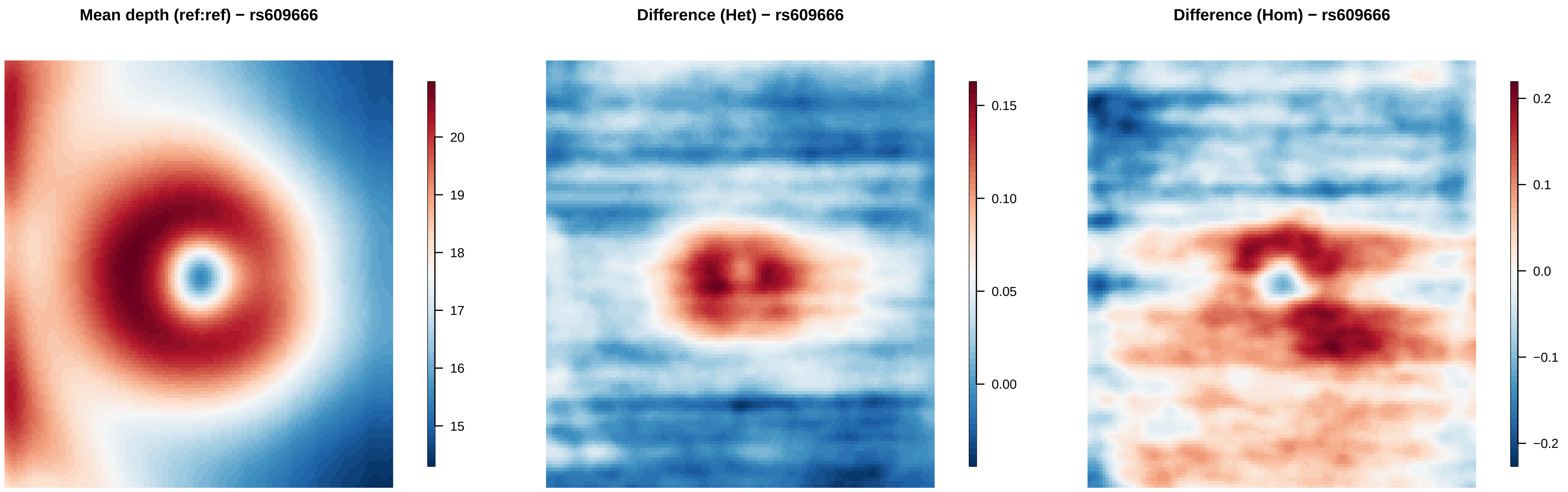
Mean depth (ref:ref) − rs609666
20
19
18
17
16
15
Difference (Het) − rs609666
0.15
0.10
0.05
0.00
Difference (Hom) − rs609666
0.2
0.1
0.0
−0.1
−0.2

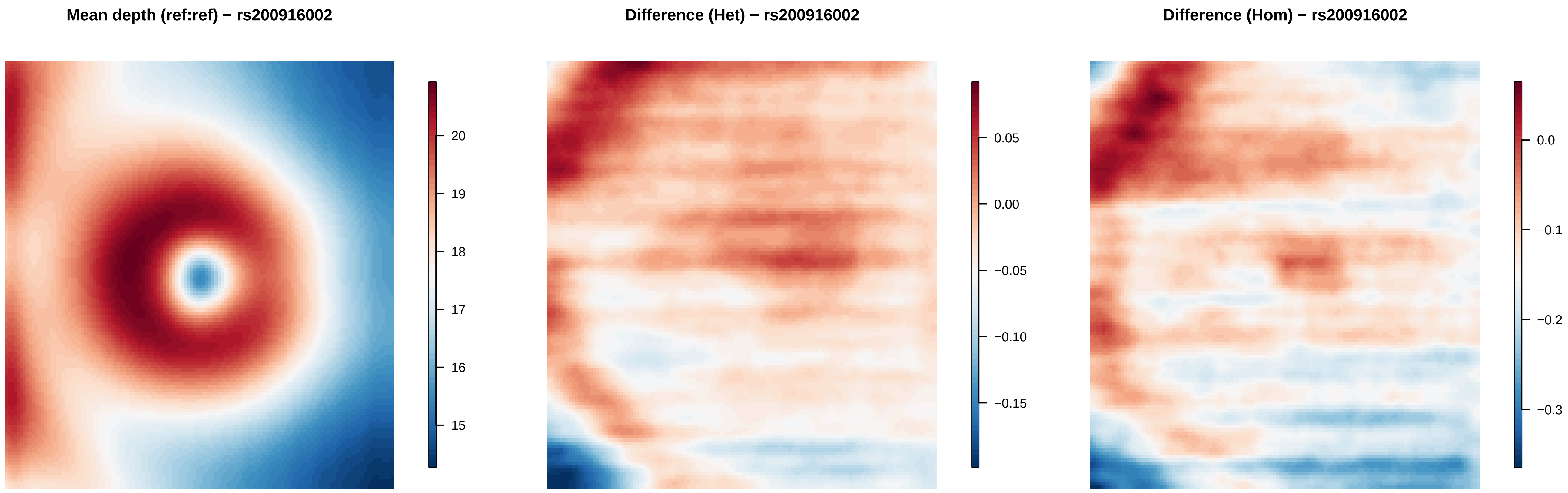
Mean depth (ref:ref) − rs200916002
20
19
18
17
16
15
Difference (Het) − rs200916002
0.05
0.00
−0.05
−0.10
−0.15
Difference (Hom) − rs200916002
0.0
−0.1
−0.2
−0.3

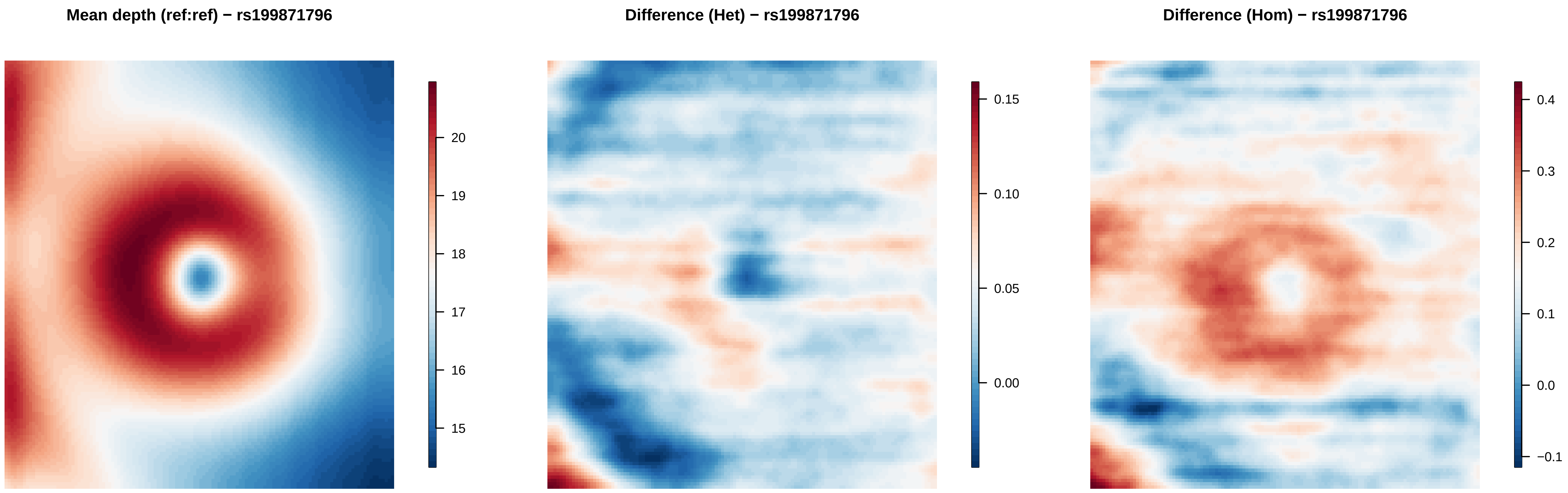

Mean depth (ref:ref) − rs199871796
20
19
18
17
16
15
Difference (Het) − rs199871796
0.15
0.10
0.05
0.00
Difference (Hom) − rs199871796
0.4
0.3
0.2
0.1
0.0
−0.1

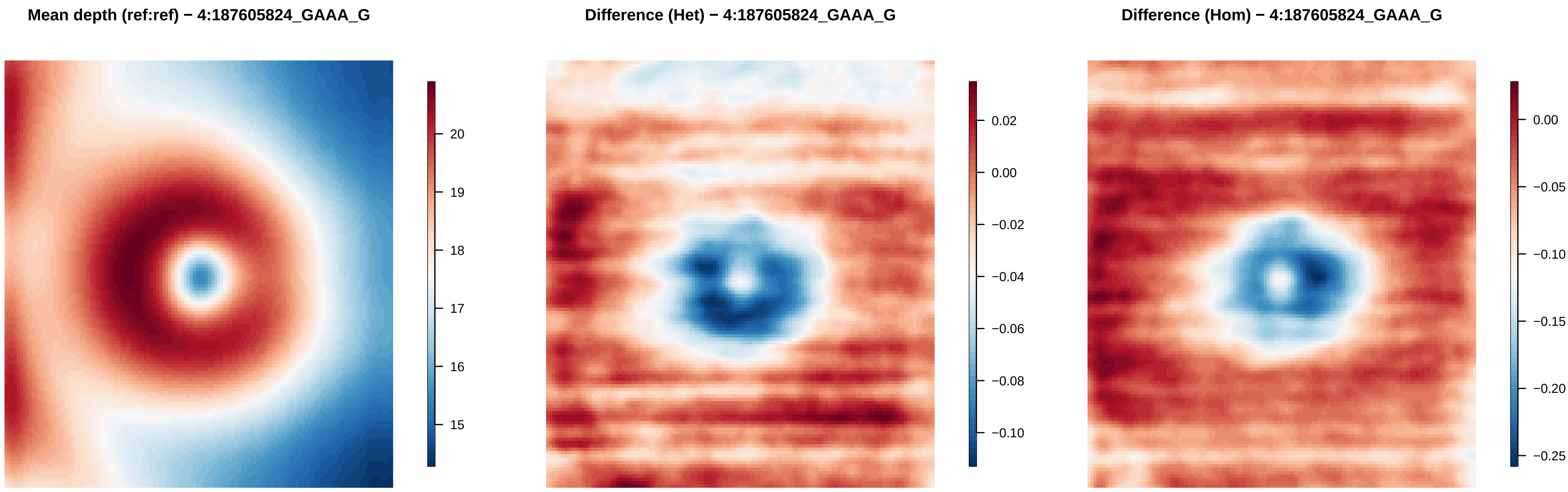

Mean depth (ref:ref) − 4:187605824_GAAA_G
20
19
18
17
16
15
Difference (Het) − 4:187605824_GAAA_G
0.02
0.00
−0.02
−0.04
−0.06
−0.08
−0.10
Difference (Hom) − 4:187605824_GAAA_G
0.00
−0.05
−0.10
−0.15
−0.20
−0.25

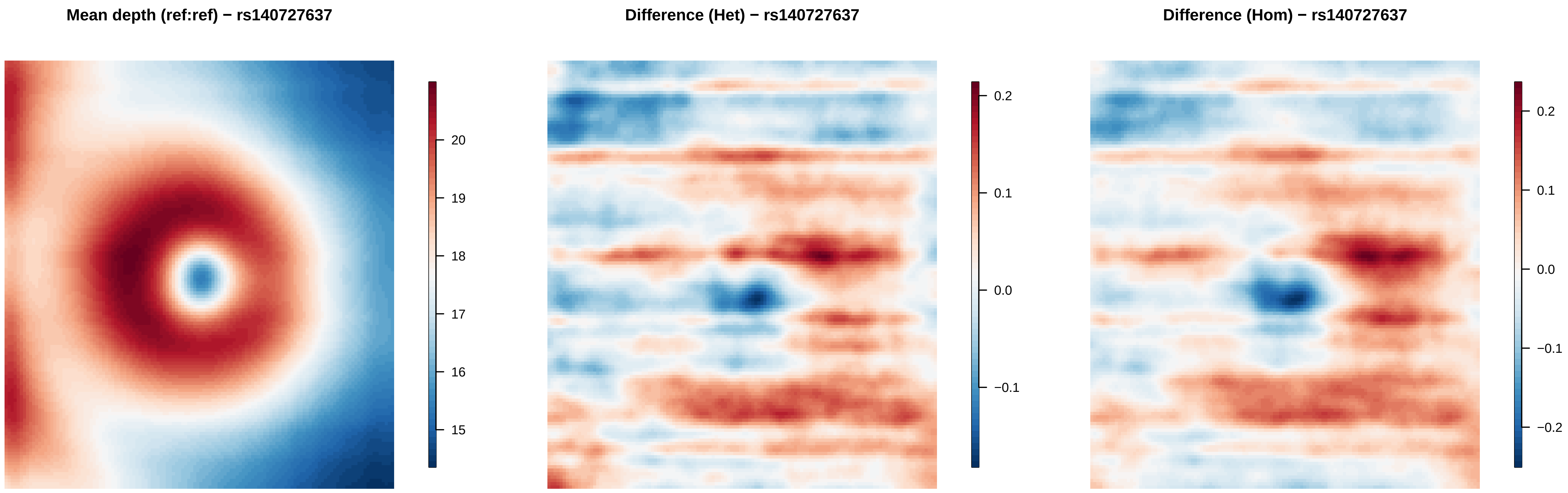
Mean depth (ref:ref) − rs140727637
20
19
18
17
16
15
Difference (Het) − rs140727637
0.2
0.1
0.0
−0.1
Difference (Hom) − rs140727637
0.2
0.1
0.0
−0.1
−0.2

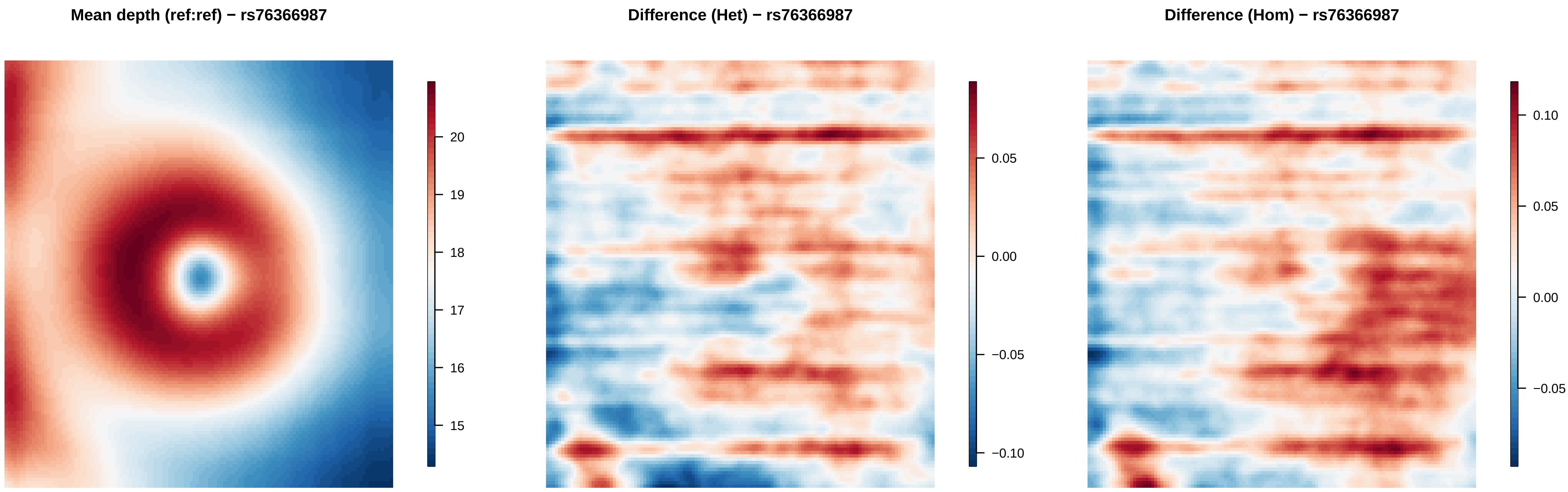

Mean depth (ref:ref) − rs76366987
20
19
18
17
16
15
Difference (Het) − rs76366987
0.05
0.00
−0.05
−0.10
Difference (Hom) − rs76366987
0.10
0.05
0.00
−0.05

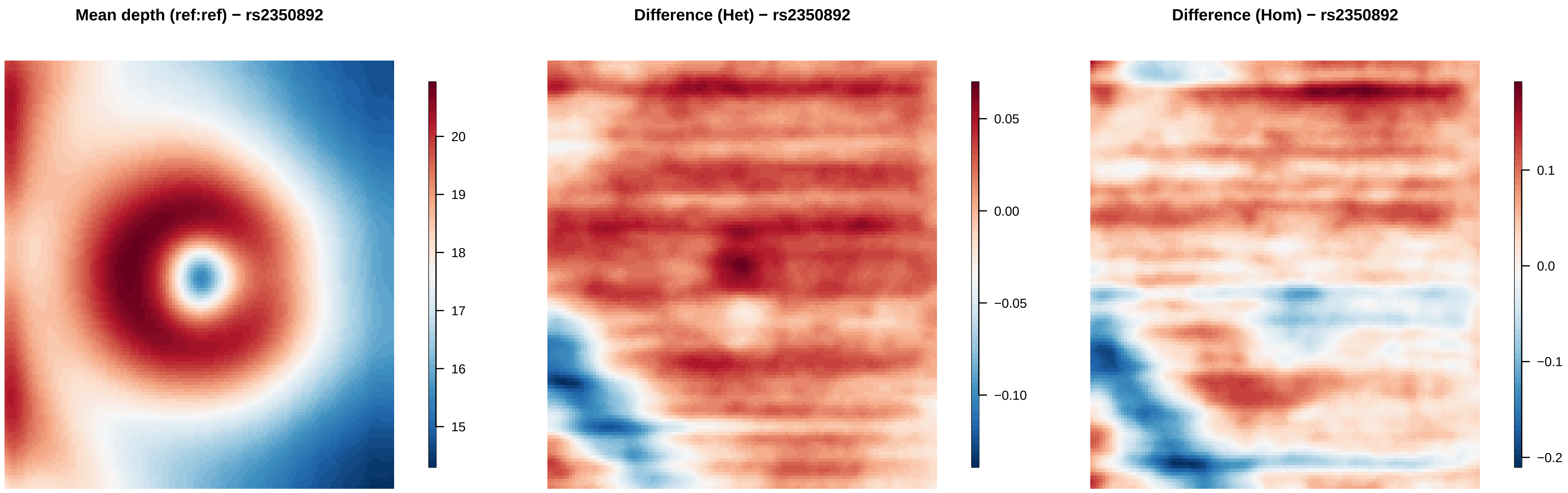
Mean depth (ref:ref) − rs2350892
20
19
18
17
16
15
Difference (Het) − rs2350892
0.05
0.00
−0.05
−0.10
Difference (Hom) − rs2350892
0.1
0.0
−0.1
−0.2

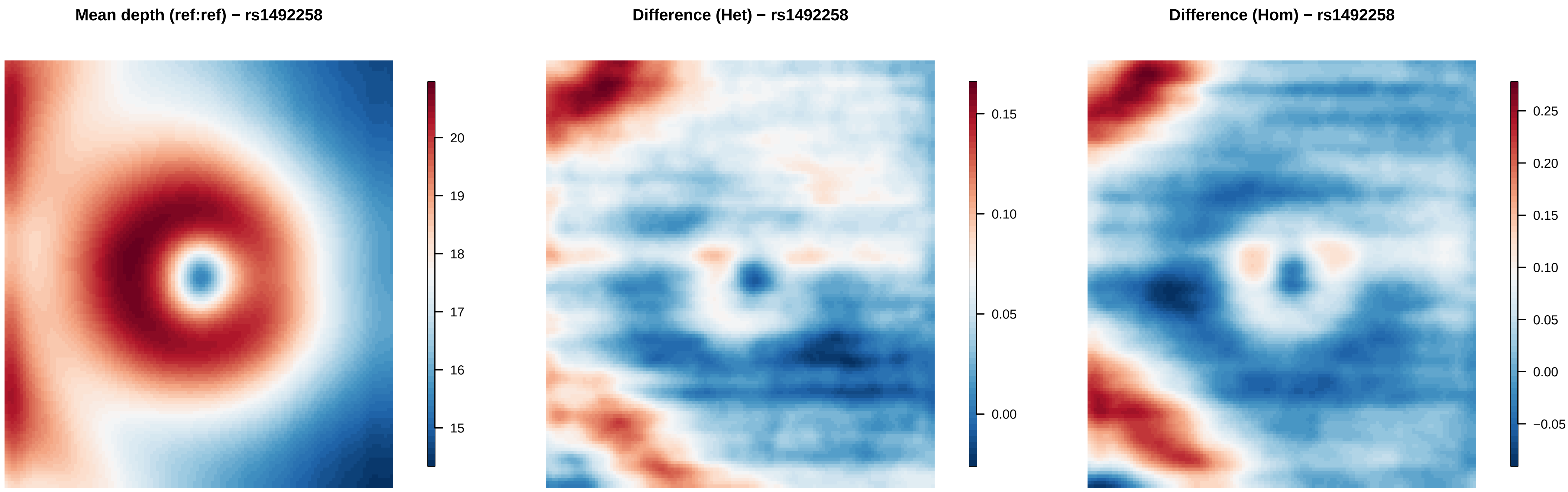
Mean depth (ref:ref) − rs1492258
20
19
18
17
16
15
Difference (Het) − rs1492258
0.15
0.10
0.05
0.00
Difference (Hom) − rs1492258
0.25
0.20
0.15
0.10
0.05
0.00
−0.05

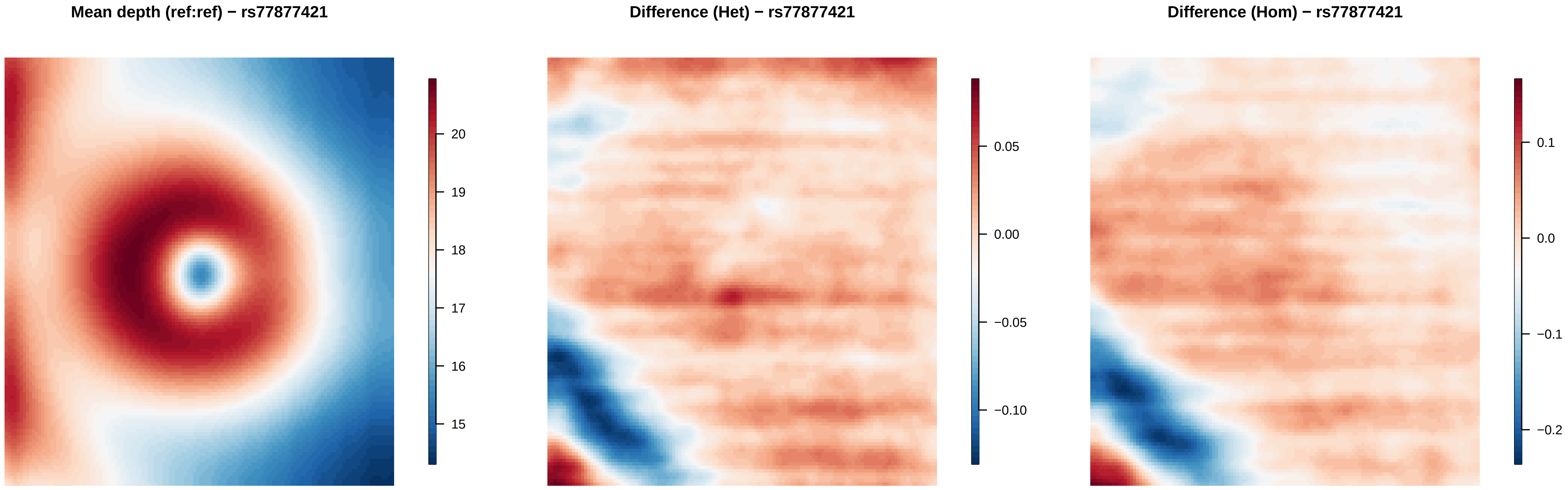
Mean depth (ref:ref) − rs77877421
20
19
18
17
16
15
Difference (Het) − rs77877421
0.05
0.00
−0.05
−0.10
Difference (Hom) − rs77877421
0.1
0.0
−0.1
−0.2

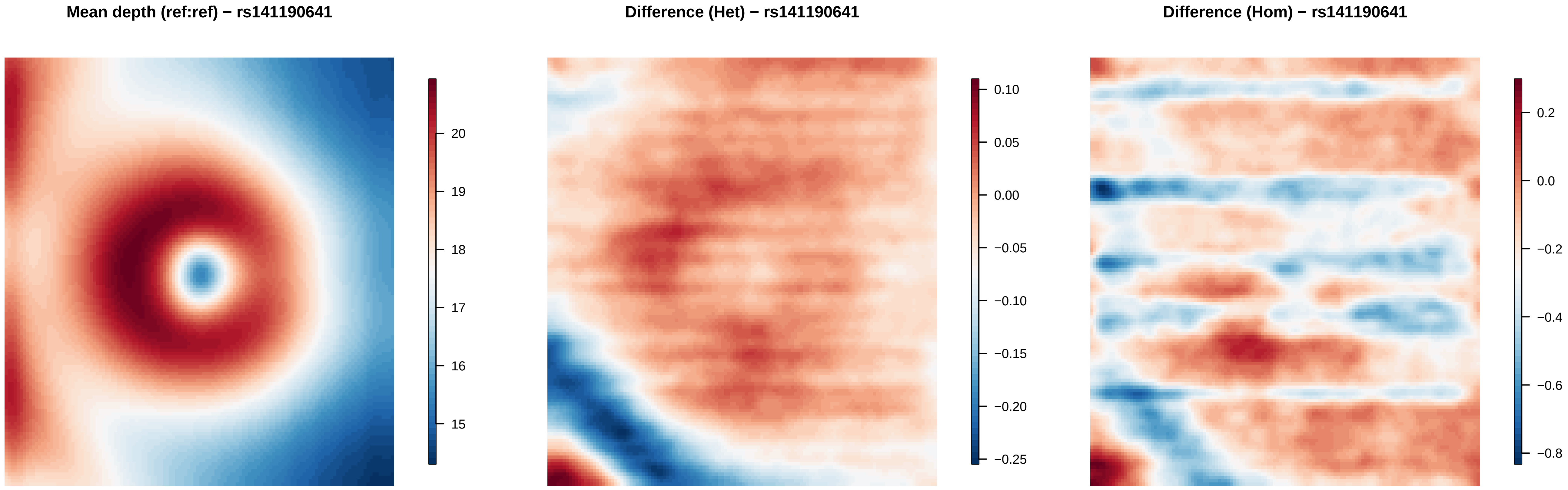
Mean depth (ref:ref) − rs141190641
20
19
18
17
16
15
Difference (Het) − rs141190641
0.10
0.05
0.00
−0.05
−0.10
−0.15
−0.20
−0.25
Difference (Hom) − rs141190641
0.2
0.0
−0.2
−0.4
−0.6
−0.8

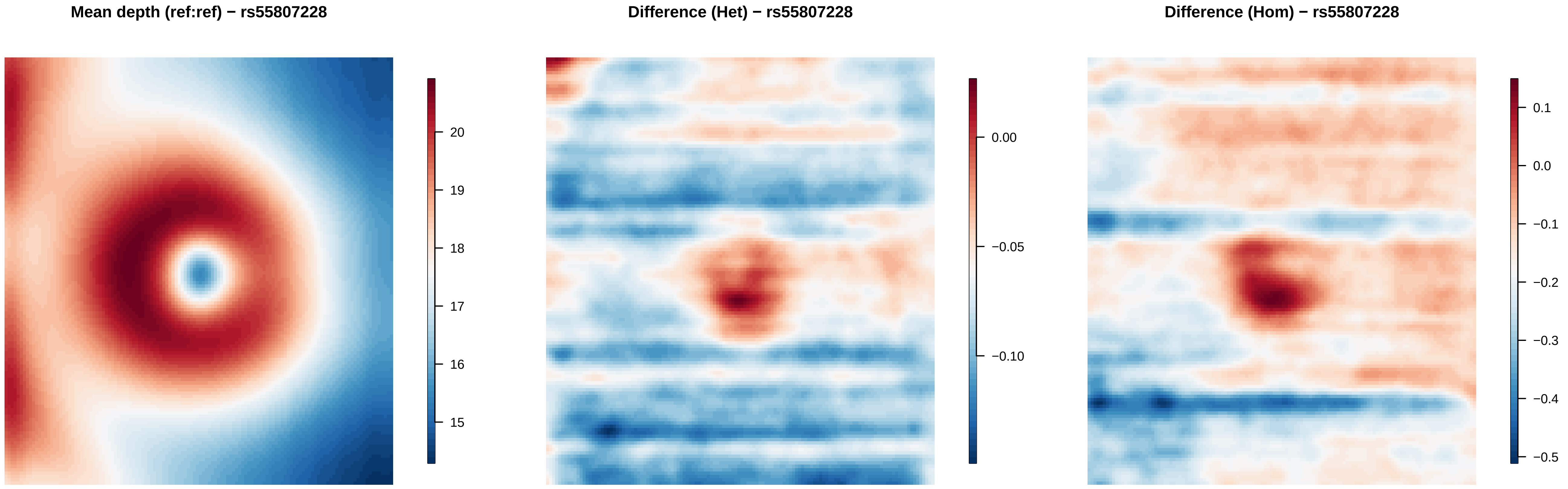
Mean depth (ref:ref) − rs55807228
20
19
18
17
16
15
Difference (Het) − rs55807228
0.00
−0.05
−0.10
Difference (Hom) − rs55807228
0.1
0.0
−0.1
−0.2
−0.3
−0.4
−0.5

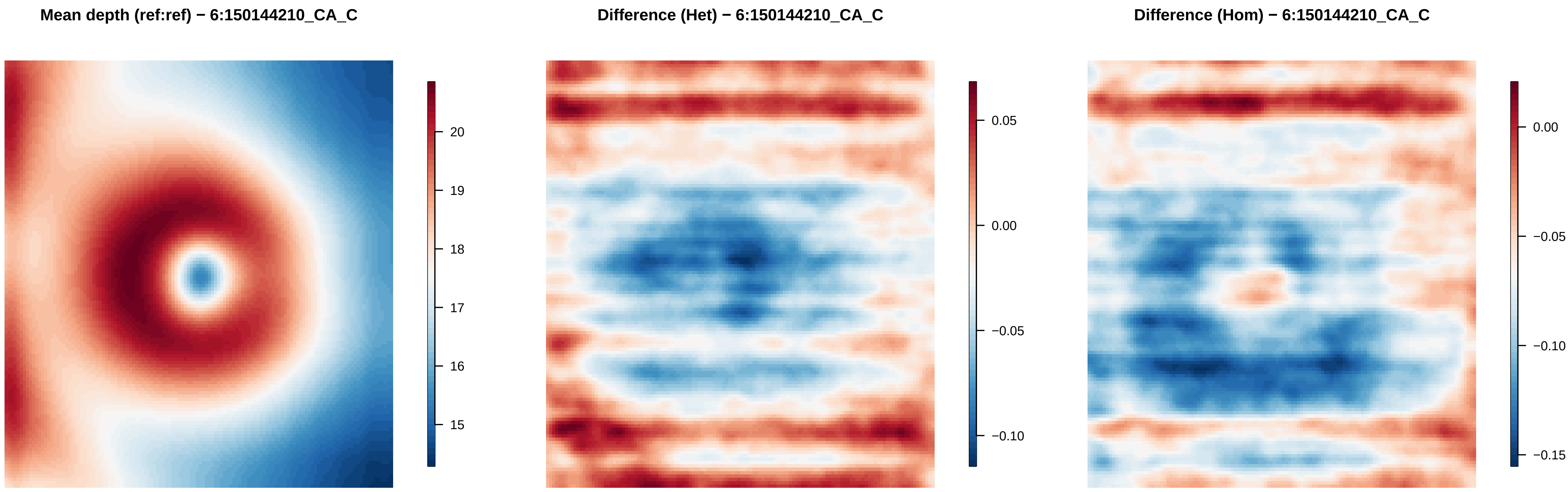
Mean depth (ref:ref) − 6:150144210_CA_C
20
19
18
17
16
15
Difference (Het) − 6:150144210_CA_C
0.05
0.00
−0.05
−0.10
Difference (Hom) − 6:150144210_CA_C
0.00
−0.05
−0.10
−0.15

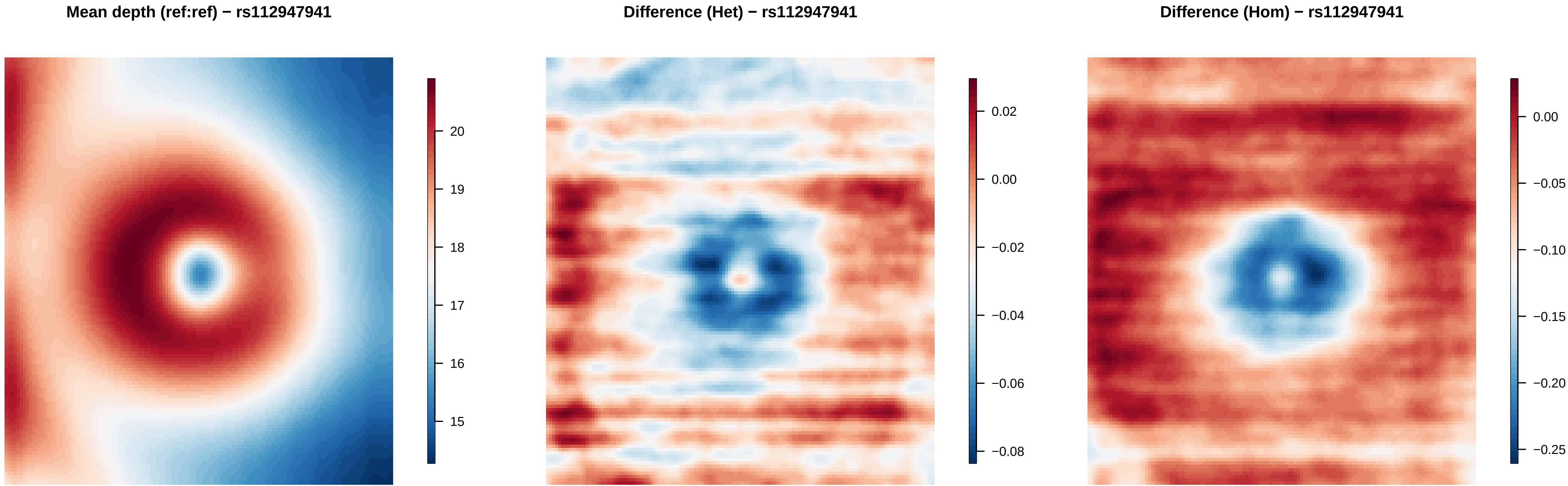
Mean depth (ref:ref) − rs112947941
20
19
18
17
16
15
Difference (Het) − rs112947941
0.02
0.00
−0.02
−0.04
−0.06
−0.08
Difference (Hom) − rs112947941
0.00
−0.05
−0.10
−0.15
−0.20
−0.25

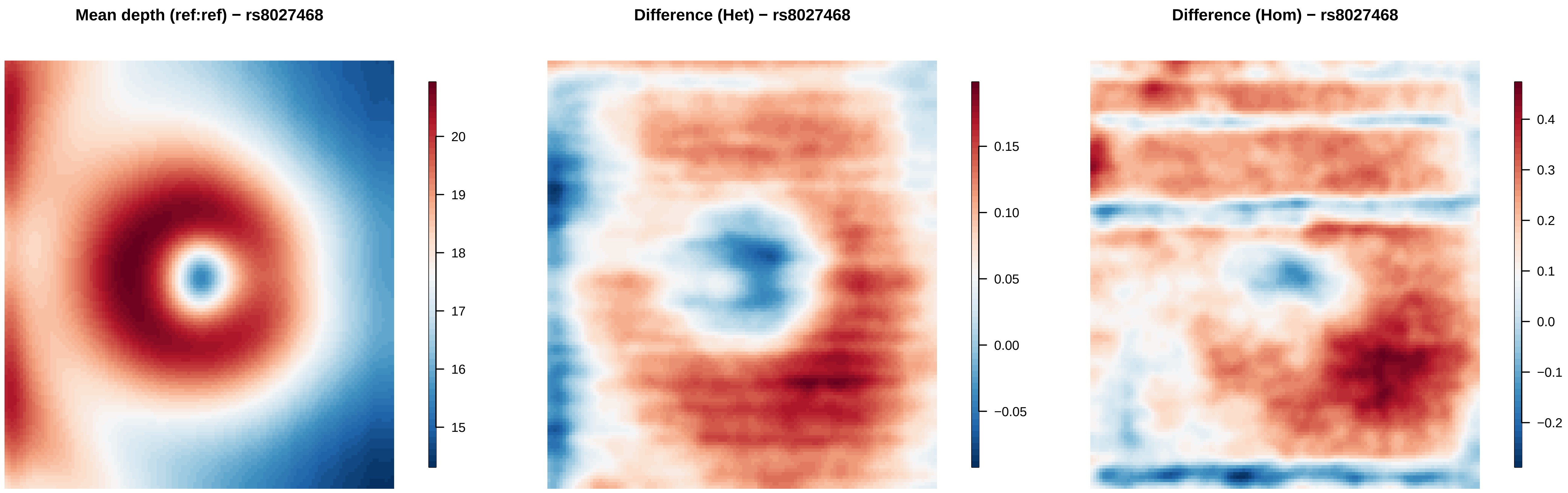
Mean depth (ref:ref) − rs8027468
20
19
18
17
16
15
Difference (Het) − rs8027468
0.15
0.10
0.05
0.00
−0.05
Difference (Hom) − rs8027468
0.4
0.3
0.2
0.1
0.0
−0.1
−0.2

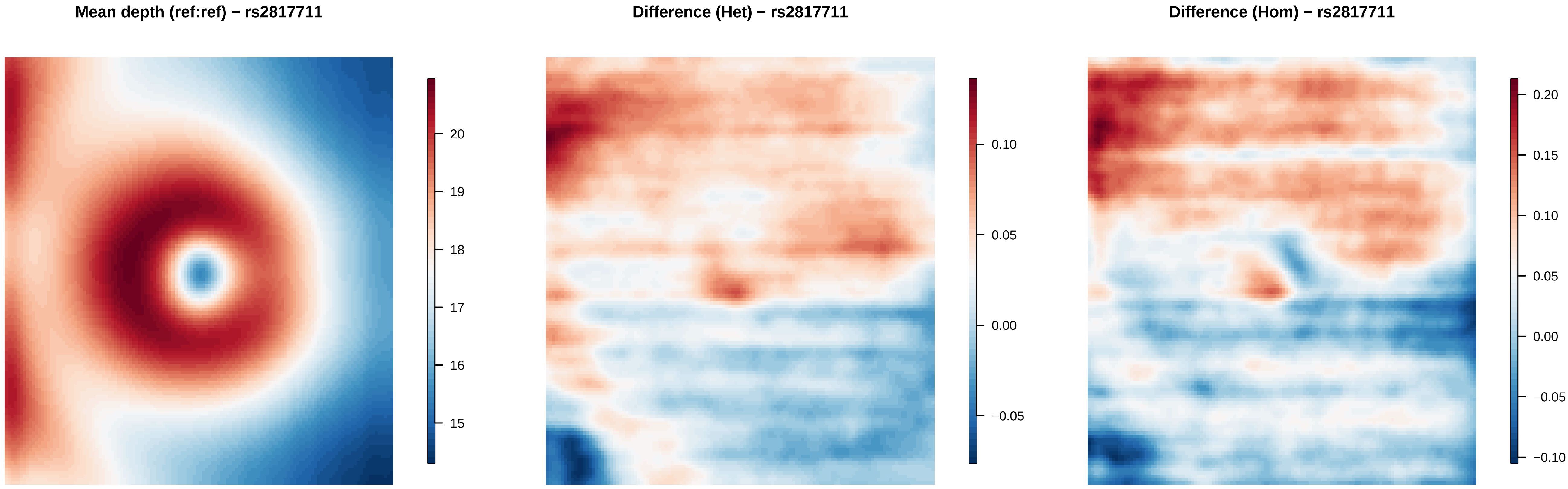

Mean depth (ref:ref) − rs2817711
20
19
18
17
16
15
Difference (Het) − rs2817711
0.10
0.05
0.00
−0.05
Difference (Hom) − rs2817711
0.20
0.15
0.10
0.05
0.00
−0.05
−0.10

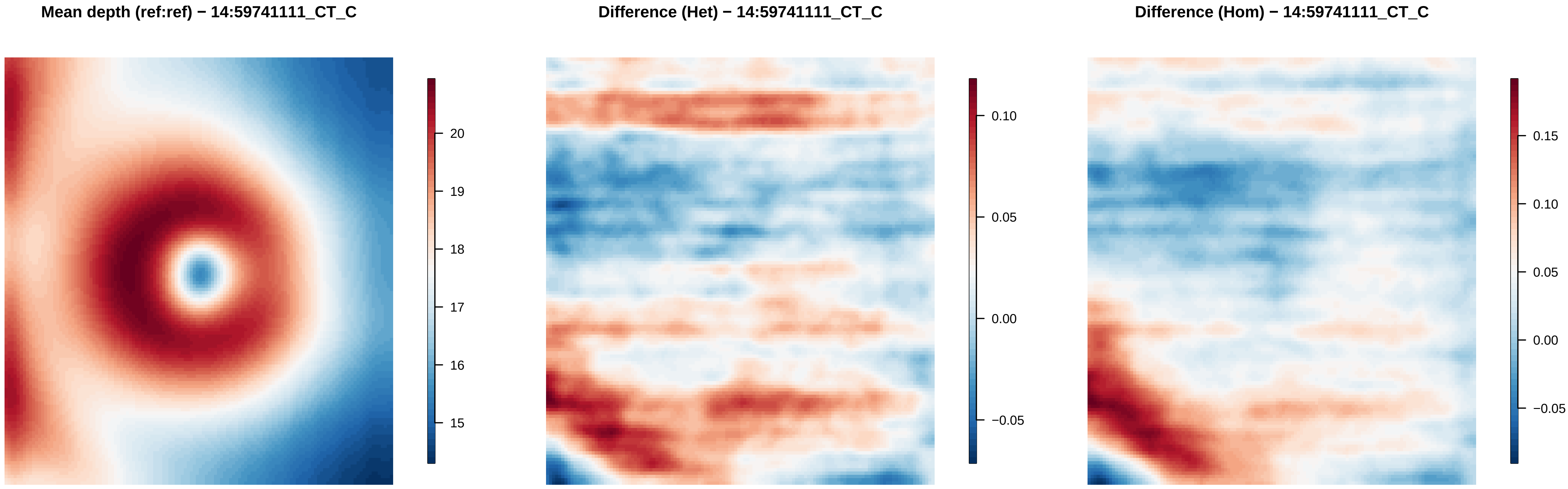
Mean depth (ref:ref) − 14:59741111_CT_C
20
19
18
17
16
15
Difference (Het) − 14:59741111_CT_C
0.10
0.05
0.00
−0.05
Difference (Hom) − 14:59741111_CT_C
0.15
0.10
0.05
0.00
−0.05

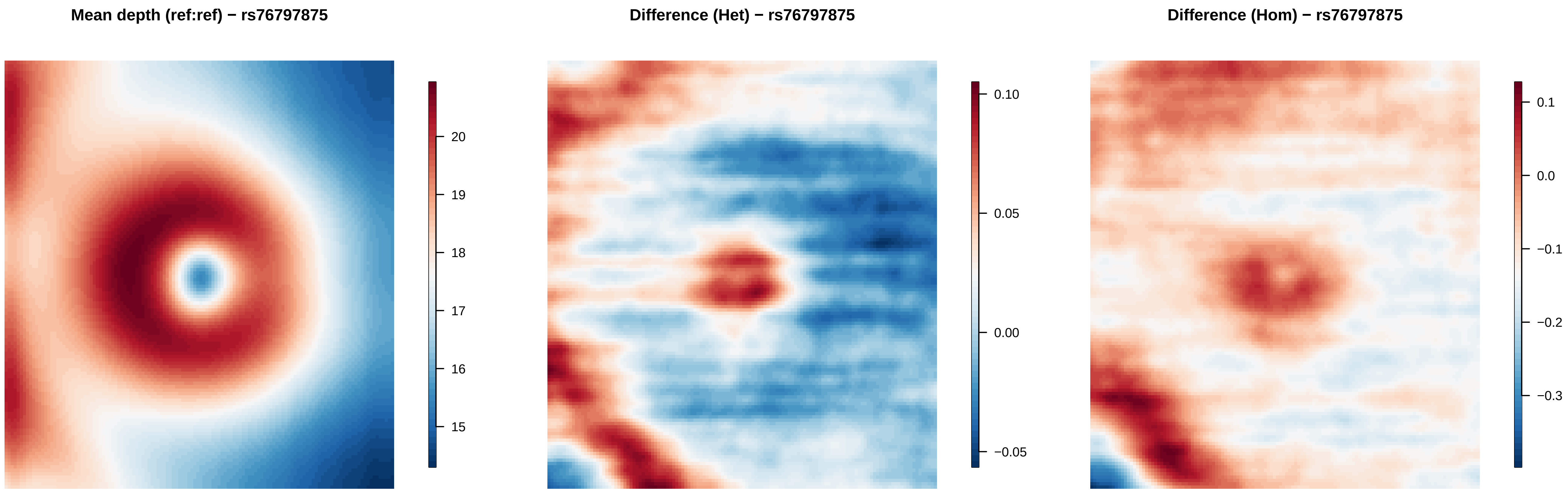
Mean depth (ref:ref) − rs76797875
20
19
18
17
16
15
Difference (Het) − rs76797875
0.10
0.05
0.00
−0.05
Difference (Hom) − rs76797875
0.1
0.0
−0.1
−0.2
−0.3

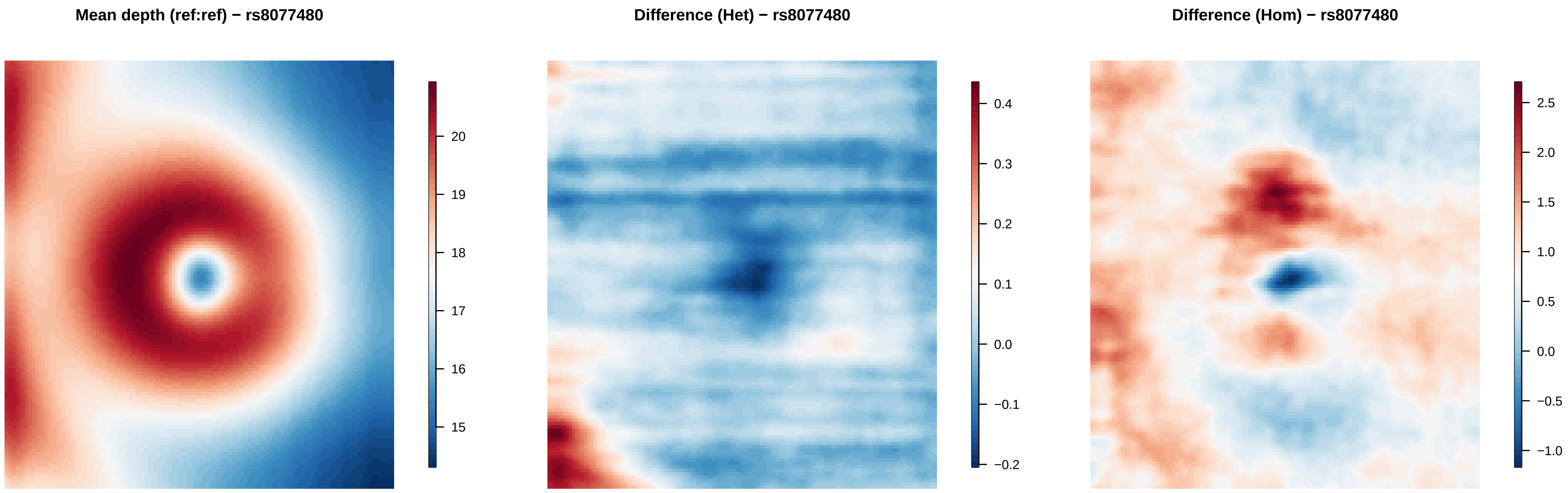

Mean depth (ref:ref) − rs8077480
20
19
18
17
16
15
Difference (Het) − rs8077480
0.4
0.3
0.2
0.1
0.0
−0.1
−0.2
Difference (Hom) − rs8077480
2.5
2.0
1.5
1.0
0.5
0.0
−0.5
−1.0

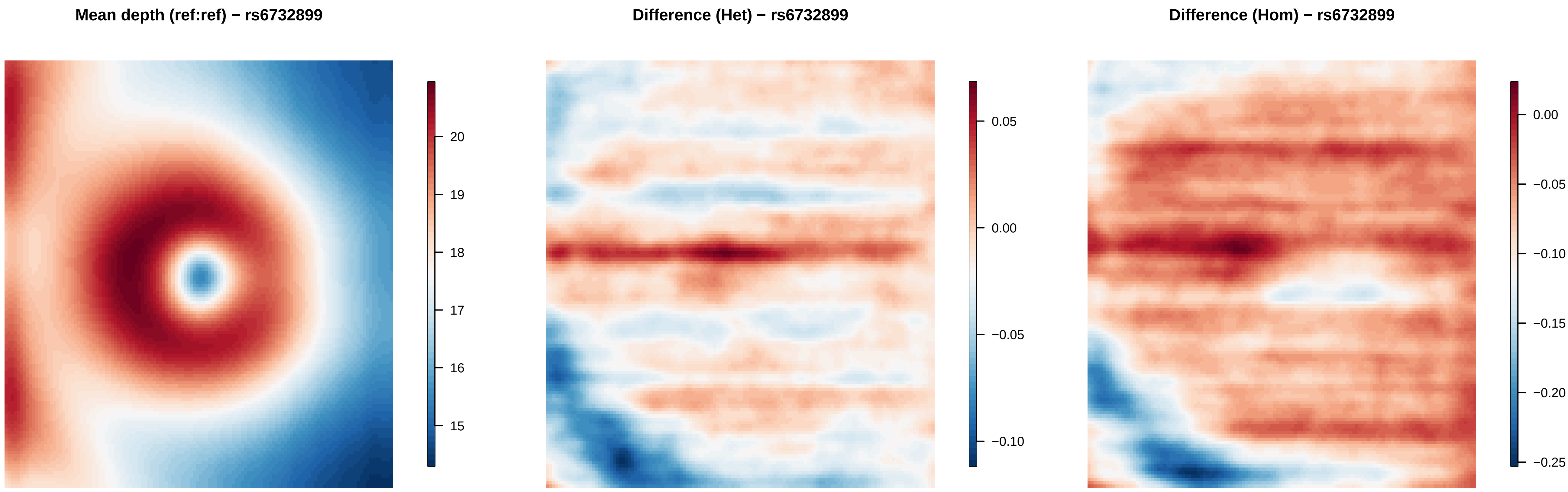

Mean depth (ref:ref) − rs6732899
20
19
18
17
16
15
Difference (Het) − rs6732899
0.05
0.00
−0.05
−0.10
Difference (Hom) − rs6732899
0.00
−0.05
−0.10
−0.15
−0.20
−0.25

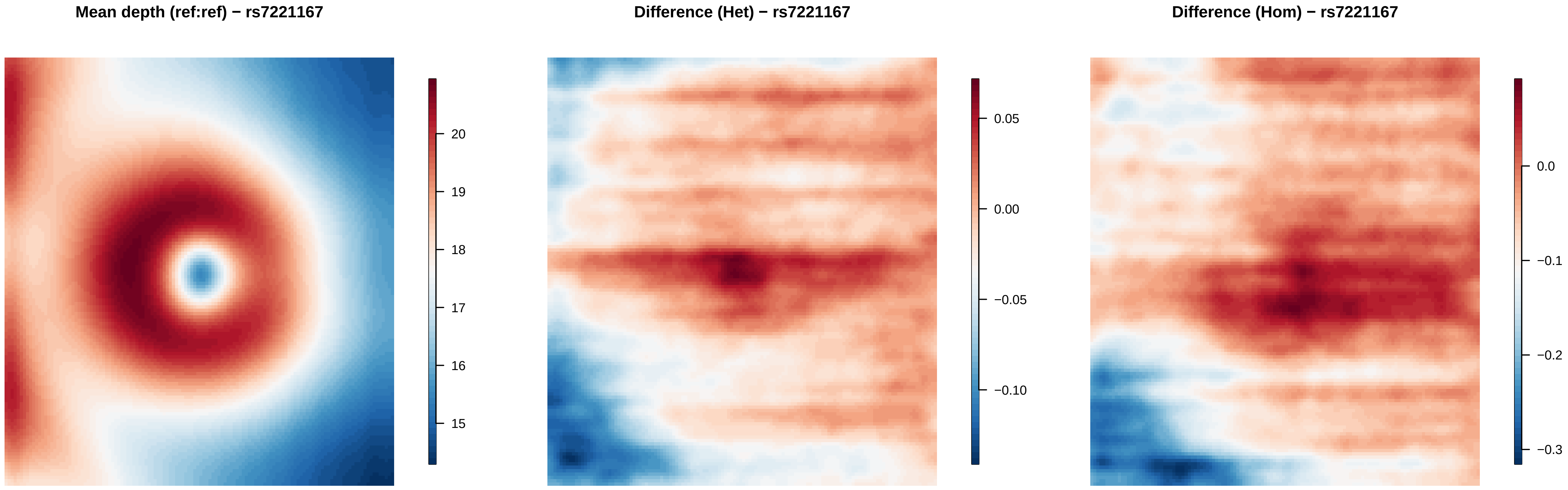
Mean depth (ref:ref) − rs7221167
20
19
18
17
16
15
Difference (Het) − rs7221167
0.05
0.00
−0.05
−0.10
Difference (Hom) − rs7221167
0.0
−0.1
−0.2
−0.3

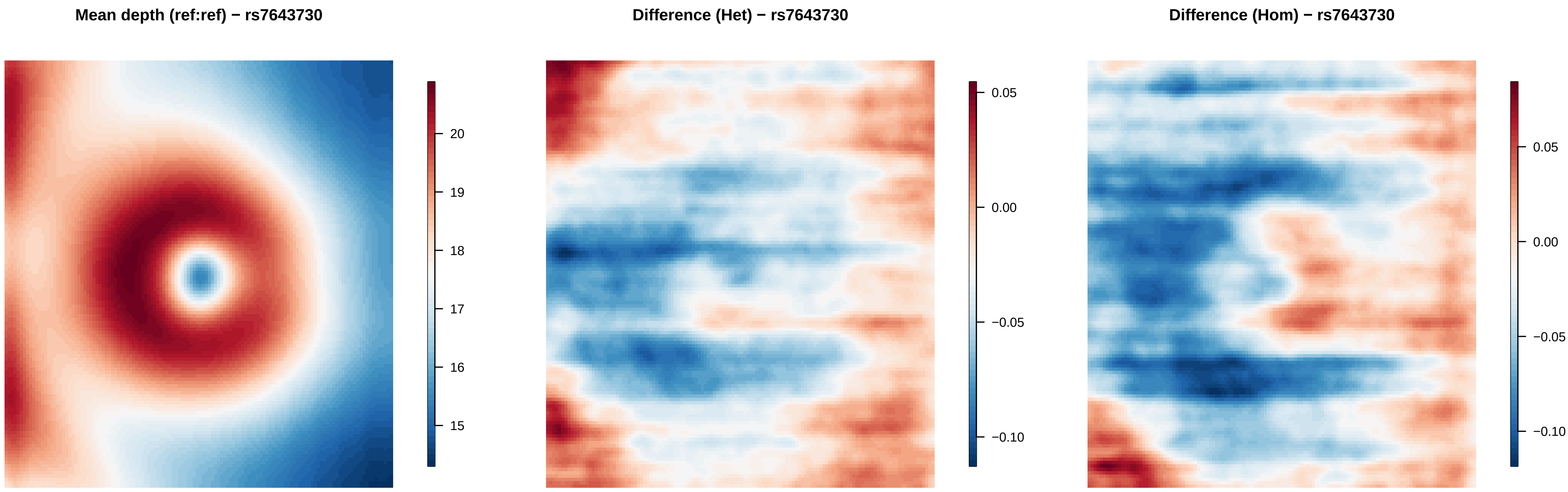
Mean depth (ref:ref) − rs7643730
20
19
18
17
16
15
Difference (Het) − rs7643730
0.05
0.00
−0.05
−0.10
Difference (Hom) − rs7643730
0.05
0.00
−0.05
−0.10

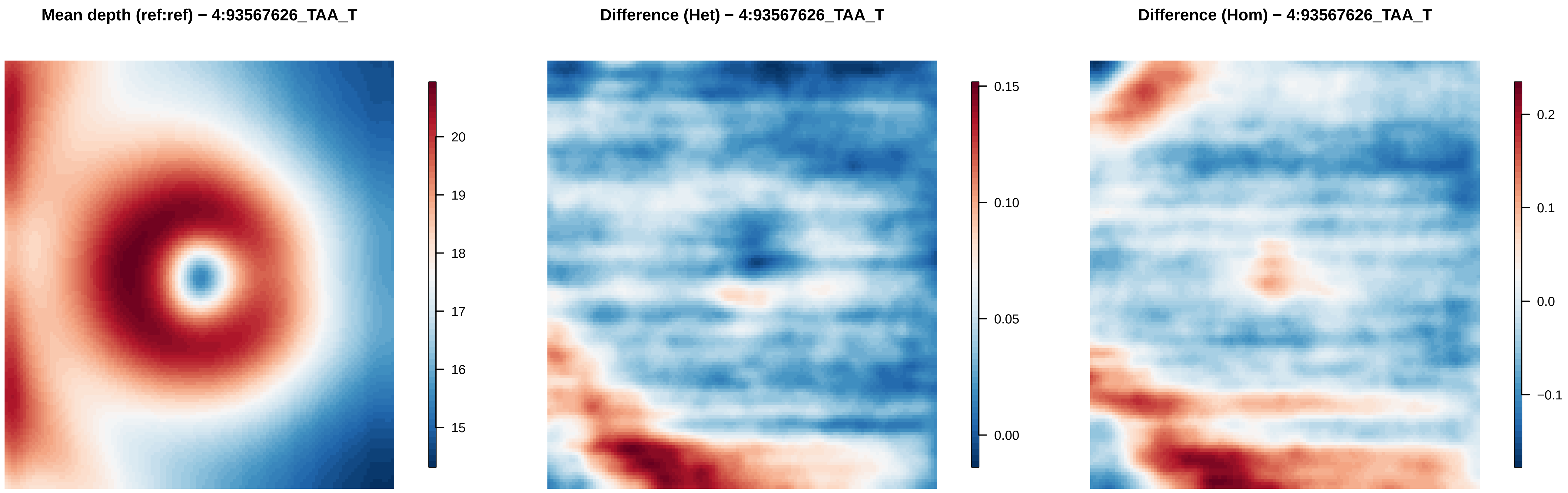

Mean depth (ref:ref) − 4:93567626_TAA_T
20
19
18
17
16
15
Difference (Het) − 4:93567626_TAA_T
0.15
0.10
0.05
0.00
Difference (Hom) − 4:93567626_TAA_T
0.2
0.1
0.0
−0.1

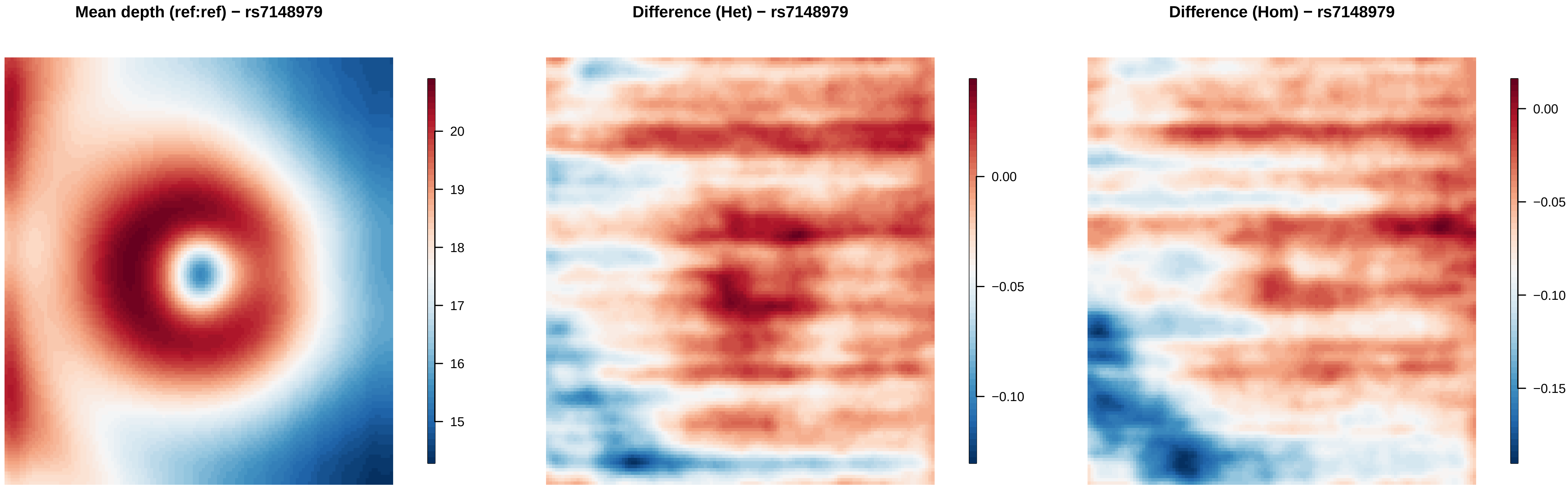

Mean depth (ref:ref) − rs7148979
20
19
18
17
16
15
Difference (Het) − rs7148979
0.00
−0.05
−0.10
Difference (Hom) − rs7148979
0.00
−0.05
−0.10
−0.15

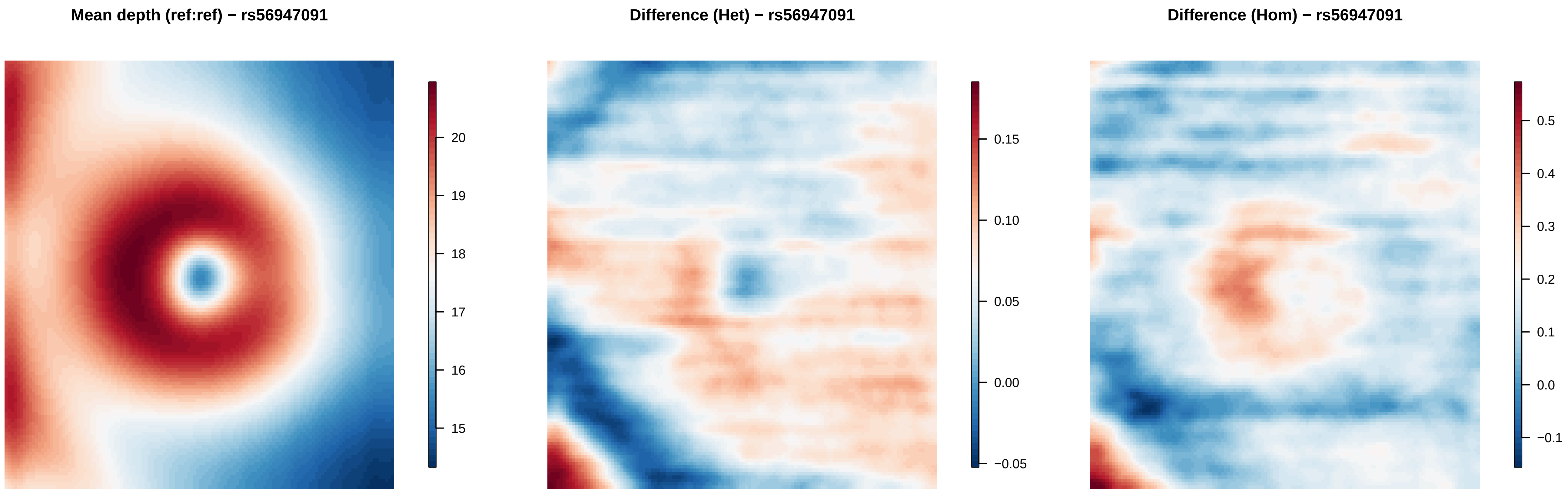

Mean depth (ref:ref) − rs56947091
20
19
18
17
16
15
Difference (Het) − rs56947091
0.15
0.10
0.05
0.00
−0.05
Difference (Hom) − rs56947091
0.5
0.4
0.3
0.2
0.1
0.0
−0.1

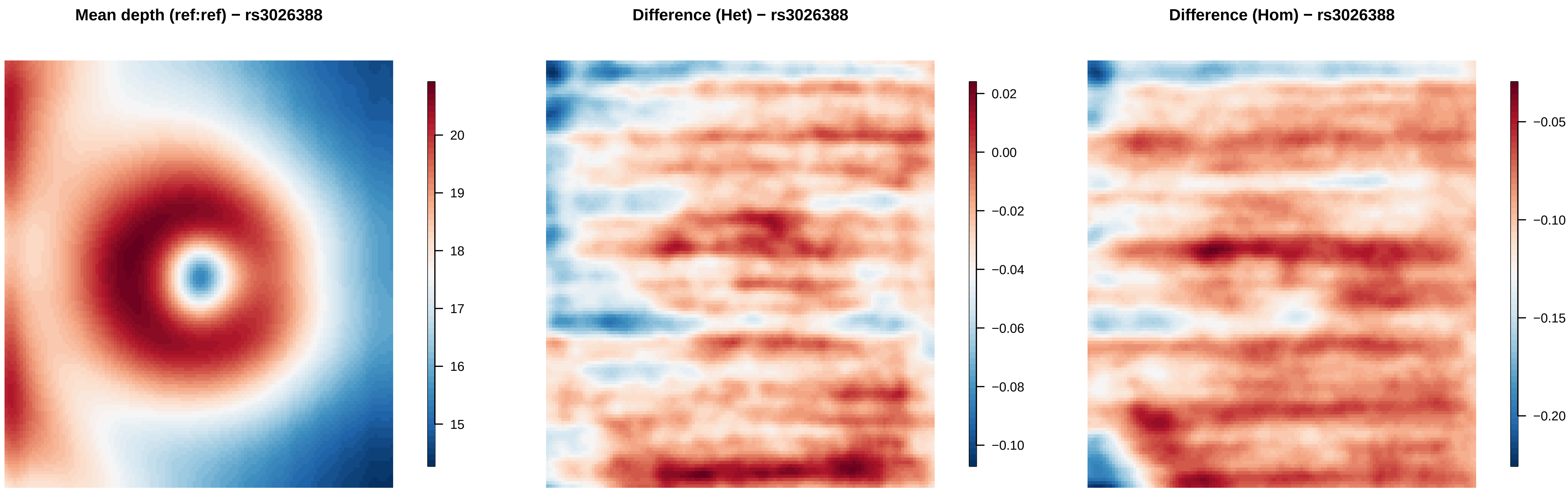
Mean depth (ref:ref) − rs3026388
20
19
18
17
16
15
Difference (Het) − rs3026388
0.02
0.00
−0.02
−0.04
−0.06
−0.08
−0.10
Difference (Hom) − rs3026388
−0.05
−0.10
−0.15
−0.20

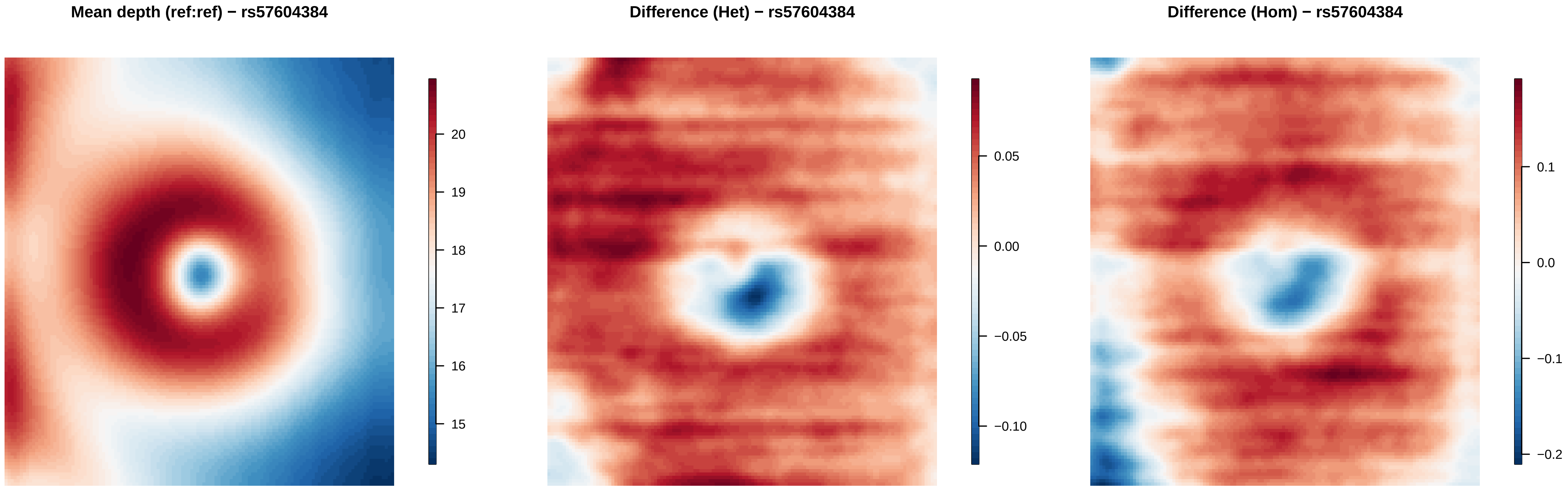
Mean depth (ref:ref) − rs57604384
20
19
18
17
16
15
Difference (Het) − rs57604384
0.05
0.00
−0.05
−0.10
Difference (Hom) − rs57604384
0.1
0.0
−0.1
−0.2

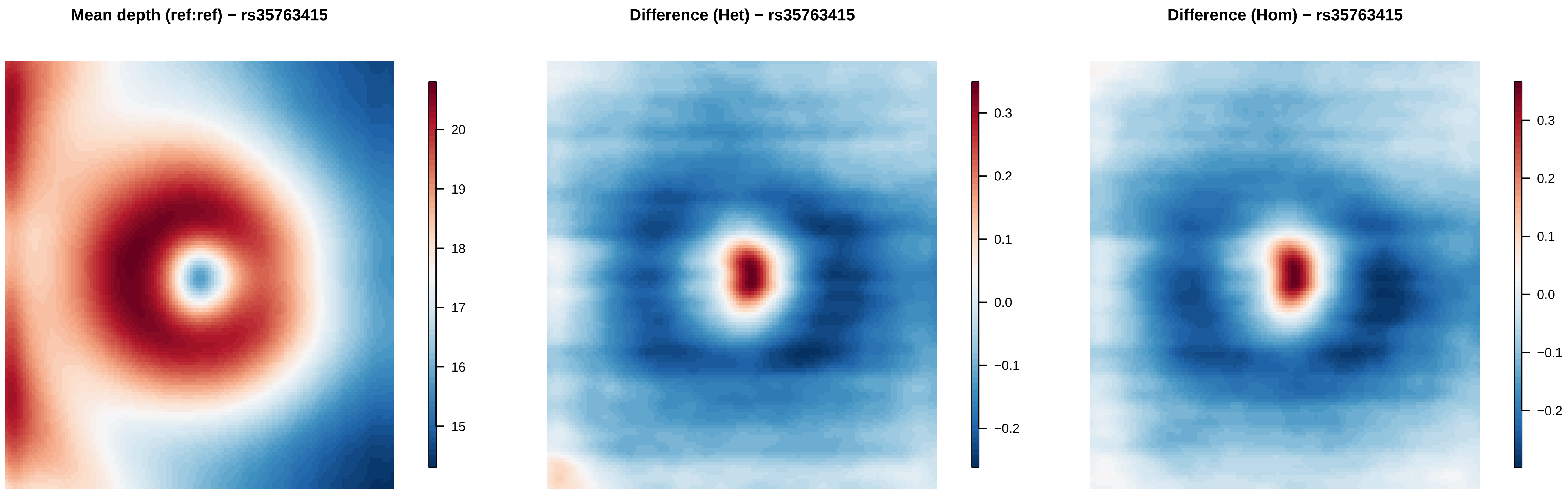
Mean depth (ref:ref) − rs35763415
20
19
18
17
16
15
Difference (Het) − rs35763415
0.3
0.2
0.1
0.0
−0.1
−0.2
Difference (Hom) − rs35763415
0.3
0.2
0.1
0.0
−0.1
−0.2

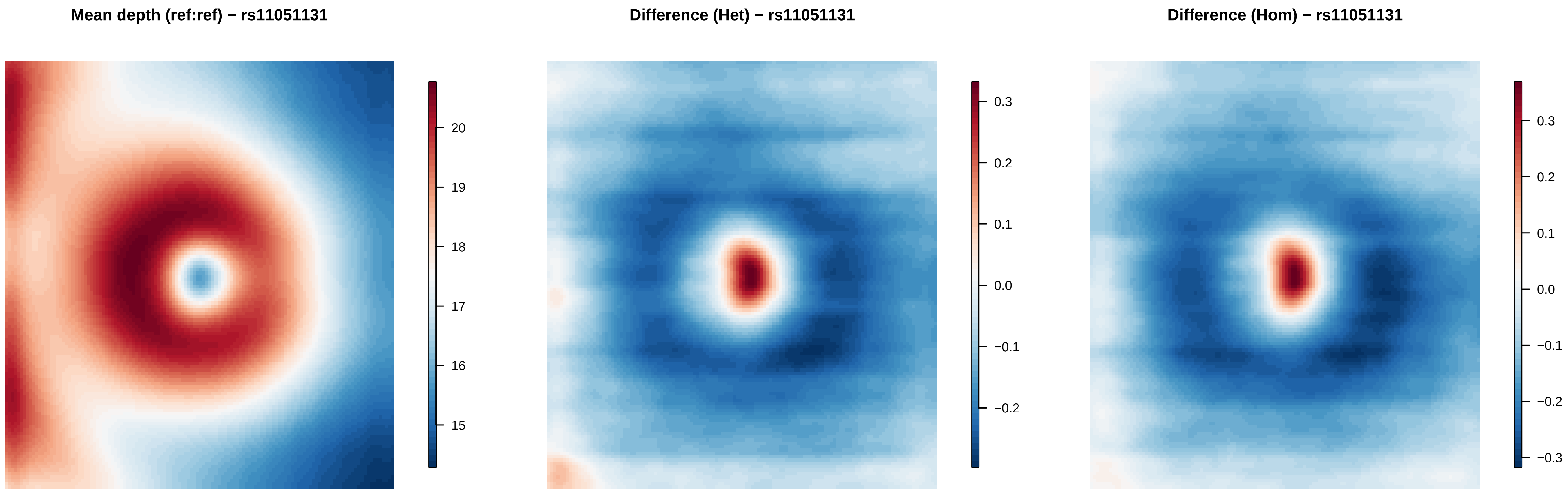
Mean depth (ref:ref) − rs11051131
20
19
18
17
16
15
Difference (Het) − rs11051131
0.3
0.2
0.1
0.0
−0.1
−0.2
Difference (Hom) − rs11051131
0.3
0.2
0.1
0.0
−0.1
−0.2
−0.3

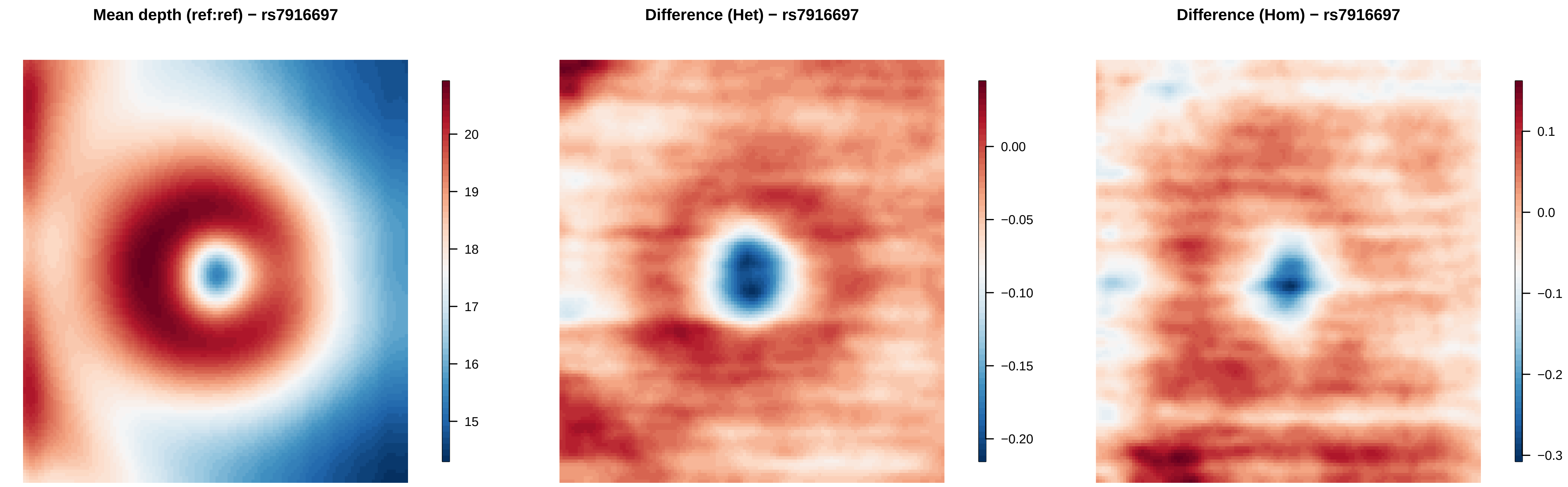

Mean depth (ref:ref) − rs7916697
20
19
18
17
16
15
Difference (Het) − rs7916697
0.00
−0.05
−0.10
−0.15
−0.20
Difference (Hom) − rs7916697
0.1
0.0
−0.1
−0.2
−0.3

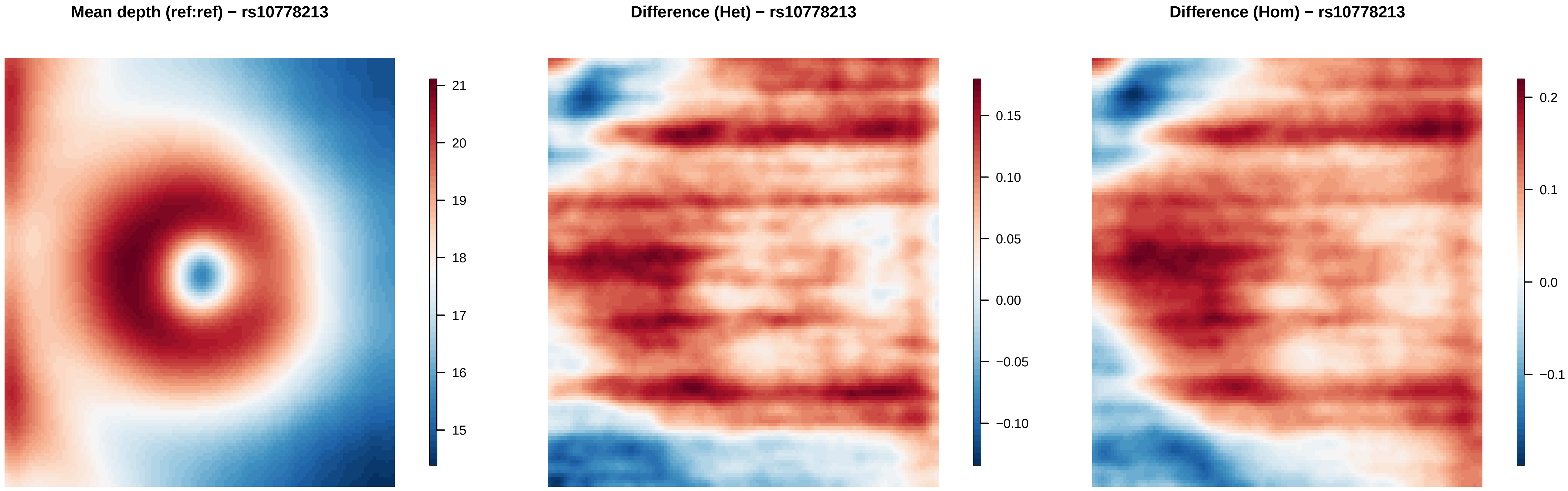
Mean depth (ref:ref) − rs10778213
21
20
19
18
17
16
15
Difference (Het) − rs10778213
0.15
0.10
0.05
0.00
−0.05
−0.10
Difference (Hom) − rs10778213
0.2
0.1
0.0
−0.1

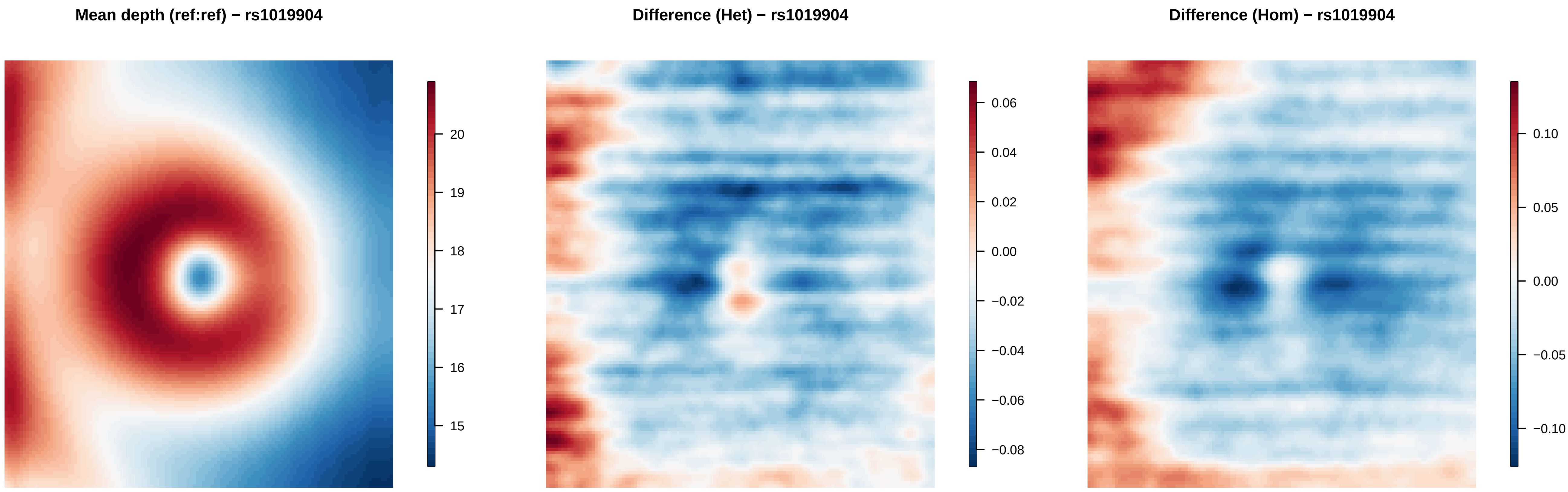

Mean depth (ref:ref) − rs1019904
20
19
18
17
16
15
Difference (Het) − rs1019904
0.06
0.04
0.02
0.00
−0.02
−0.04
−0.06
−0.08
Difference (Hom) − rs1019904
0.10
0.05
0.00
−0.05
−0.10

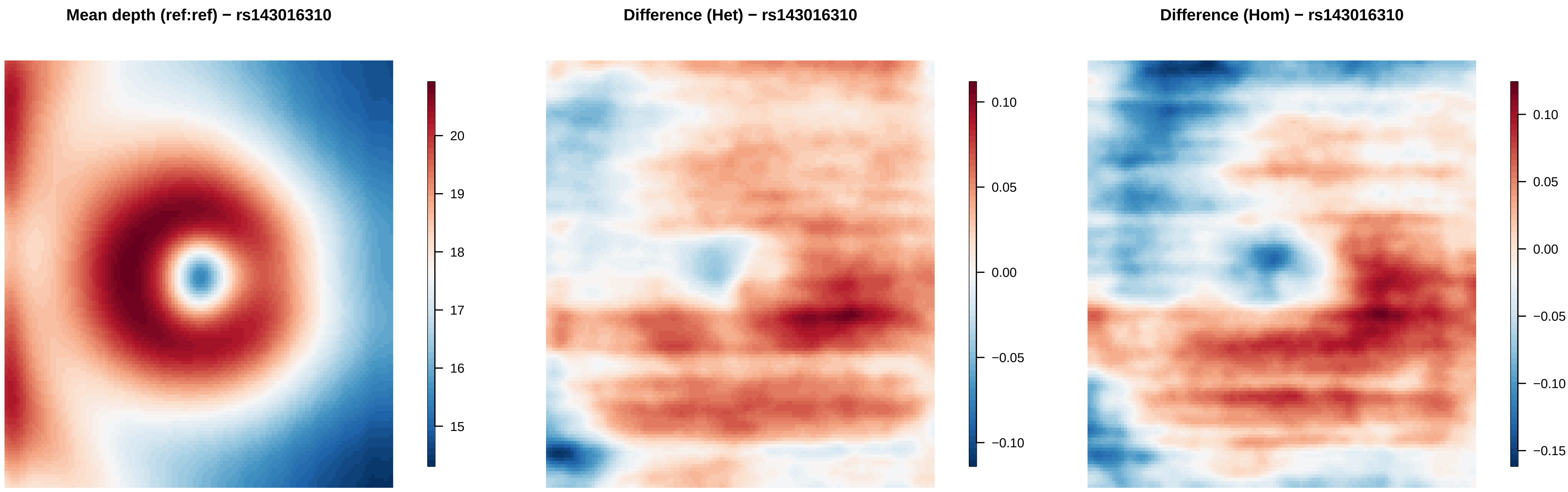
Mean depth (ref:ref) − rs143016310
20
19
18
17
16
15
Difference (Het) − rs143016310
0.10
0.05
0.00
−0.05
−0.10
Difference (Hom) − rs143016310
0.10
0.05
0.00
−0.05
−0.10
−0.15

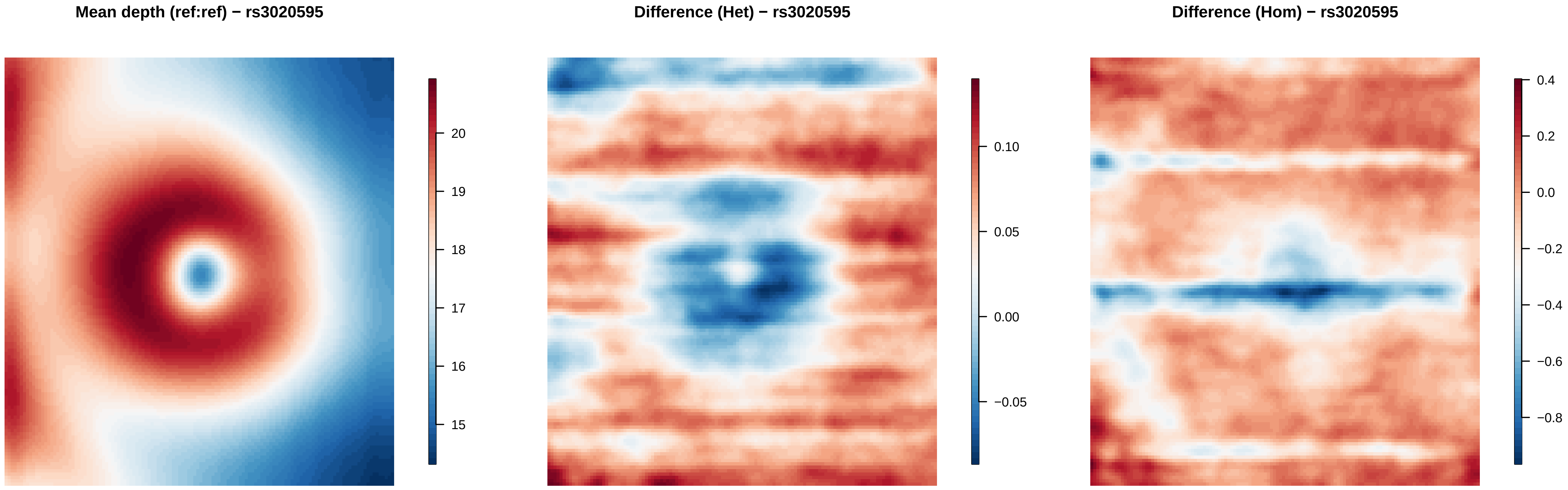
Mean depth (ref:ref) − rs3020595
20
19
18
17
16
15
Difference (Het) − rs3020595
0.10
0.05
0.00
−0.05
Difference (Hom) − rs3020595
0.4
0.2
0.0
−0.2
−0.4
−0.6
−0.8

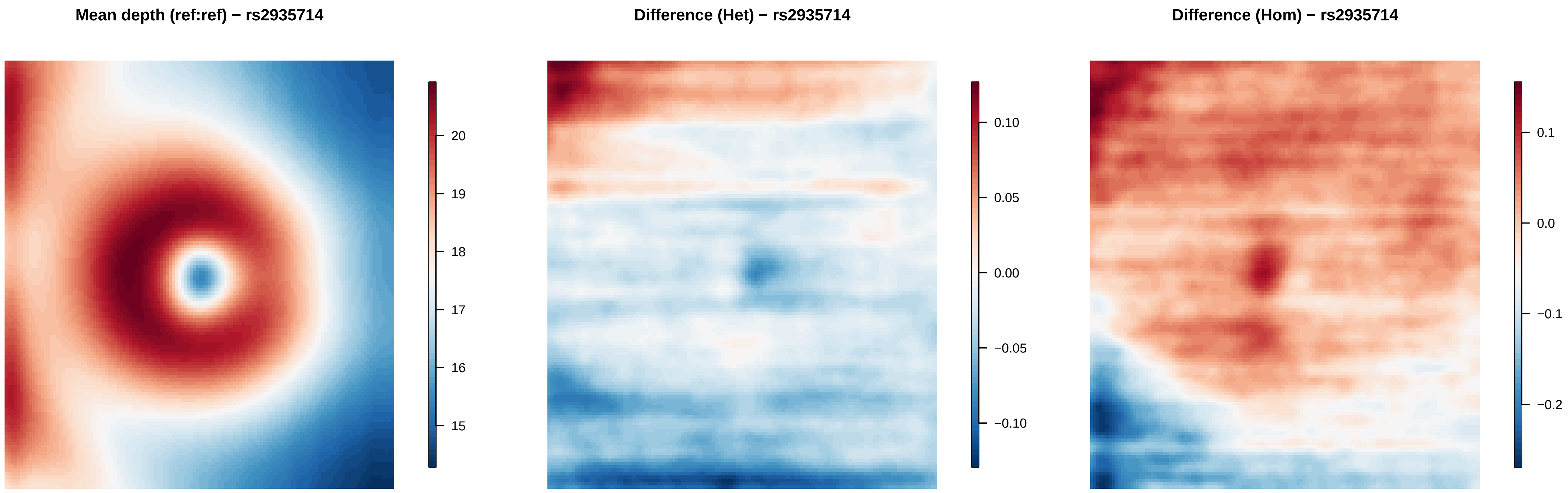

Mean depth (ref:ref) − rs2935714
20
19
18
17
16
15
Difference (Het) − rs2935714
0.10
0.05
0.00
−0.05
−0.10
Difference (Hom) − rs2935714
0.1
0.0
−0.1
−0.2

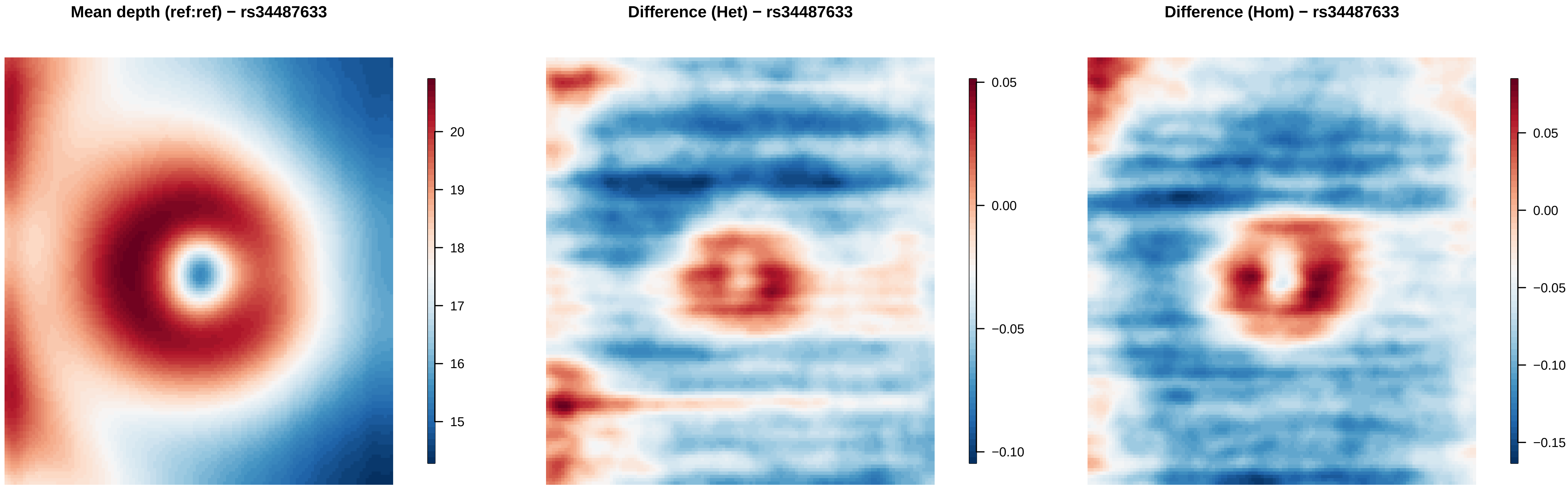
Mean depth (ref:ref) − rs34487633
20
19
18
17
16
15
Difference (Het) − rs34487633
0.05
0.00
−0.05
−0.10
Difference (Hom) − rs34487633
0.05
0.00
−0.05
−0.10
−0.15

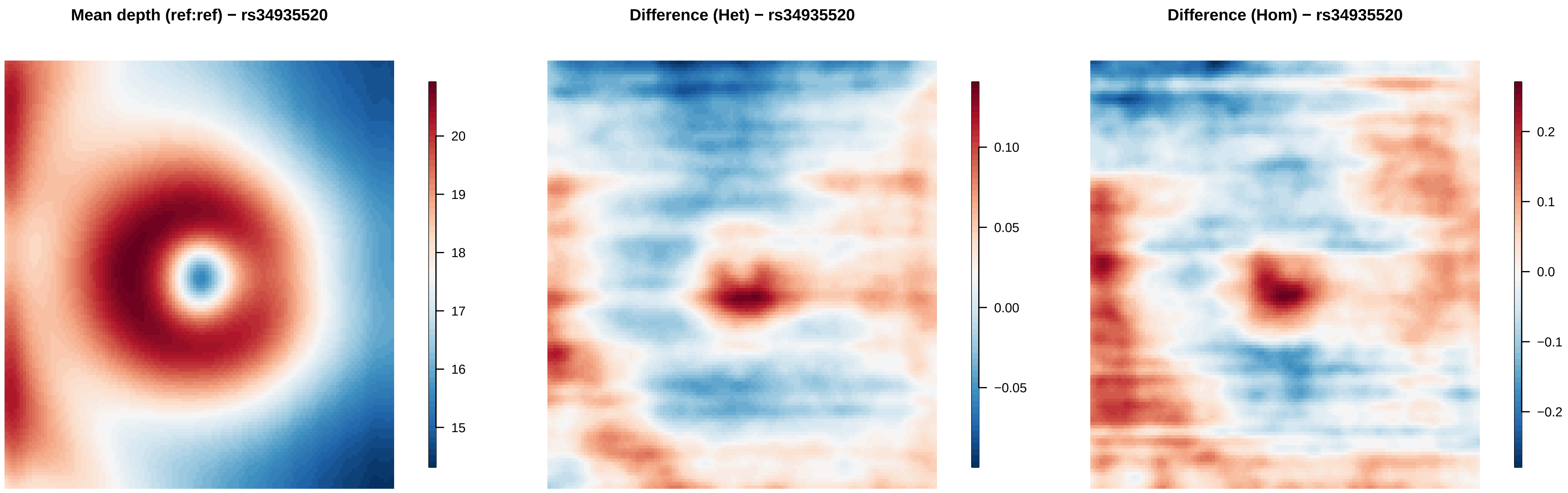
Mean depth (ref:ref) − rs34935520
20
19
18
17
16
15
Difference (Het) − rs34935520
0.10
0.05
0.00
−0.05
Difference (Hom) − rs34935520
0.2
0.1
0.0
−0.1
−0.2

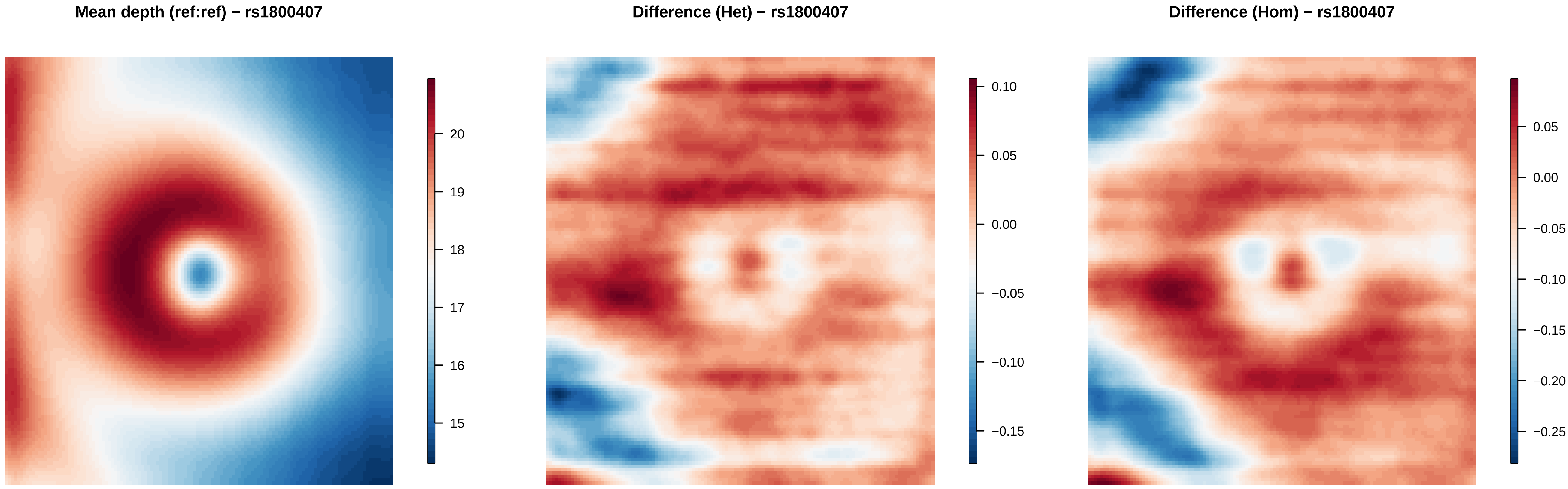
Mean depth (ref:ref) − rs1800407
20
19
18
17
16
15
Difference (Het) − rs1800407
0.10
0.05
0.00
−0.05
−0.10
−0.15
Difference (Hom) − rs1800407
0.05
0.00
−0.05
−0.10
−0.15
−0.20
−0.25

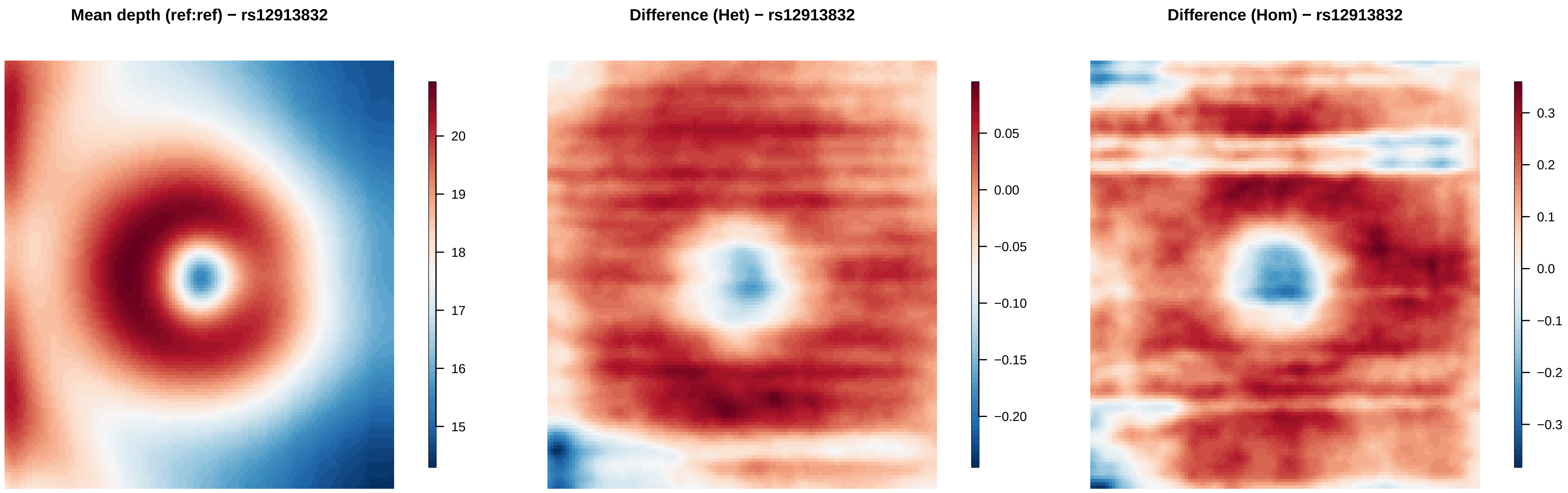
Mean depth (ref:ref) − rs12913832
20
19
18
17
16
15
Difference (Het) − rs12913832
0.05
0.00
−0.05
−0.10
−0.15
−0.20
Difference (Hom) − rs12913832
0.3
0.2
0.1
0.0
−0.1
−0.2
−0.3

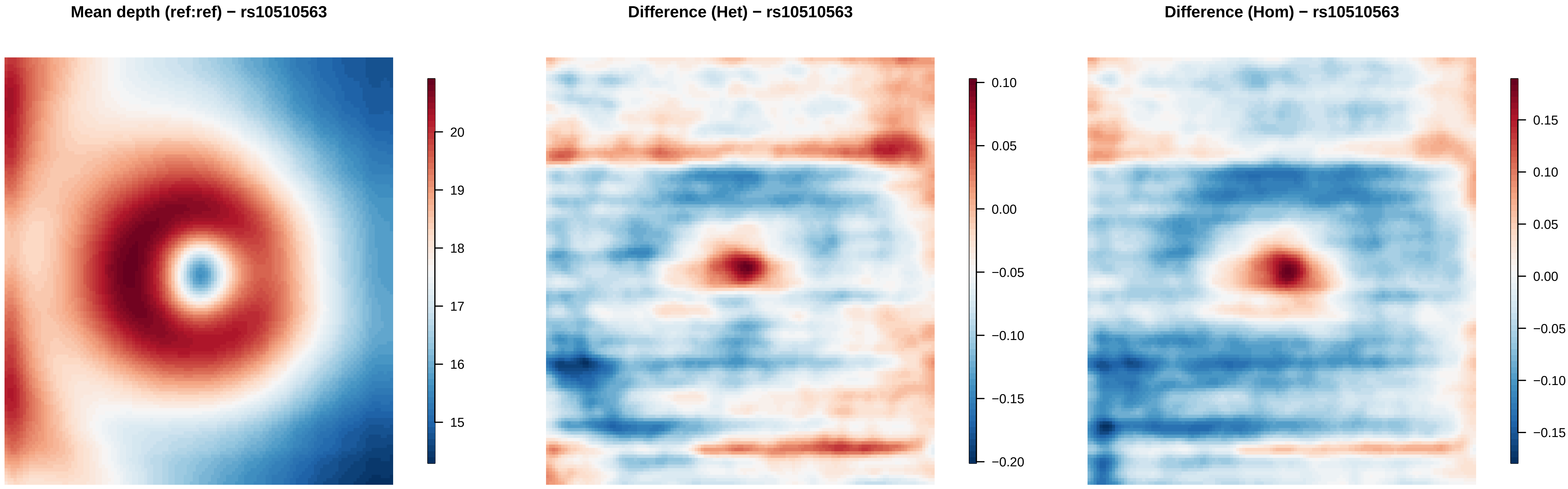
Mean depth (ref:ref) − rs10510563
20
19
18
17
16
15
Difference (Het) − rs10510563
0.10
0.05
0.00
−0.05
−0.10
−0.15
−0.20
Difference (Hom) − rs10510563
0.15
0.10
0.05
0.00
−0.05
−0.10
−0.15

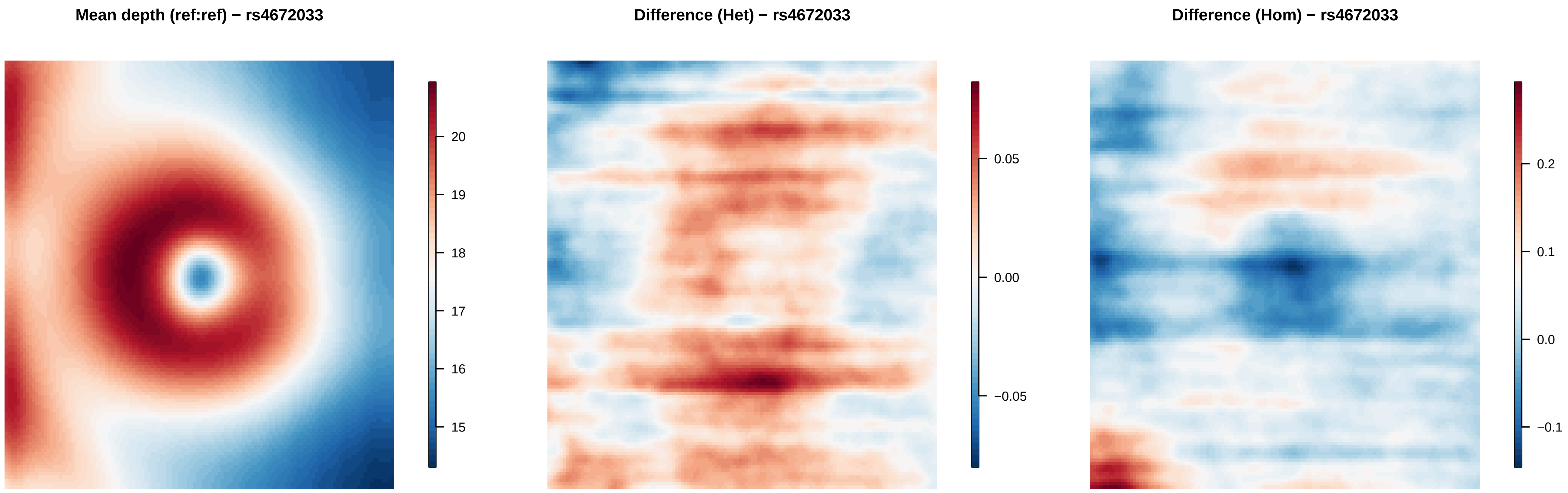

Mean depth (ref:ref) − rs4672033
20
19
18
17
16
15
Difference (Het) − rs4672033
0.05
0.00
−0.05
Difference (Hom) − rs4672033
0.2
0.1
0.0
−0.1

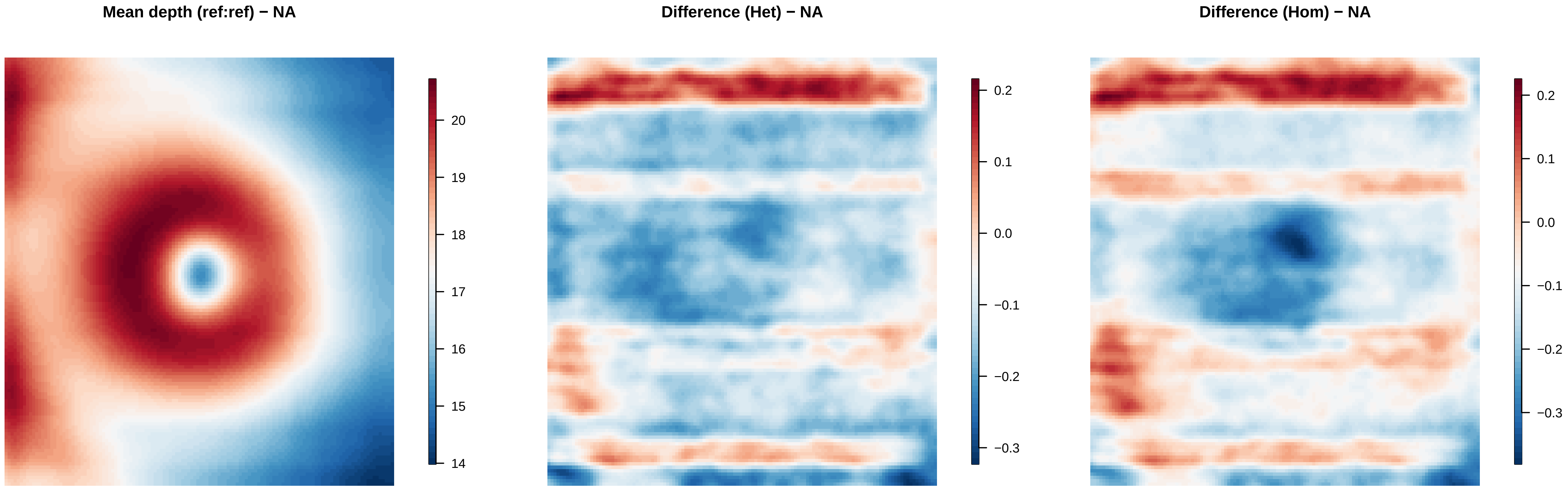
Mean depth (ref:ref) − NA
20
19
18
17
16
15
14
Difference (Het) − NA
0.2
0.1
0.0
−0.1
−0.2
−0.3
Difference (Hom) − NA
0.2
0.1
0.0
−0.1
−0.2
−0.3
